# In Vivo Studies on Radiofrequency (100 kHz–300 GHz) Electromagnetic Field Exposure and Cancer: A Systematic Review

**DOI:** 10.3390/ijerph20032071

**Published:** 2023-01-23

**Authors:** Rosanna Pinto, Lucia Ardoino, Paola Villani, Carmela Marino

**Affiliations:** Division Health Protection Technology at ENEA, Italian National Agency for New Technologies, Energy, Environment and Sustainable Economic Development, 00123 Rome, Italy

**Keywords:** radiofrequency-electromagnetic fields, health effect, carcinogenesis, tumor incidence, animal studies, systematic review

## Abstract

The increasing exposure of the human population to radiofrequency electromagnetic fields has increased concern about its possible health effects. The aim of this systematic review is to provide an update of the state of the research on this topic, through a quantitative analysis, to assess the increased risk of tumor incidence in laboratory animals (rodents) without limitations of species, strain, sex or genotype. The review was conducted according to the PRISMA guideline and individual studies were assessed by referring to the OHAT Risk of Bias Rating Tool for Human and Animal Studies. A total of 27 studies were considered eligible for the evaluation of tumor incidence; a meta-analysis was carried out on 23 studies to assess the possible increased risk of both malignant and benign tumors onset at the systemic level or in different organs/tissues. A significant association between exposure to RF and the increased/decreased risk of cancer does not result from the meta-analysis in most of considered tissues. A significant increased/decreased risk can be numerically observed only in heart, CNS/brain, and intestine for malignant tumors. Nevertheless, the assessment of the body of evidence attributes low or inadequate evidence for an association between RF exposure and the onset of neoplasm in all tissues.

## 1. Introduction

Over the past decades, exposure to radio frequency (100 kHz–300 GHz) electromagnetic fields (RF-EMF) has steadily increased, causing growing concerns for human health. Consequently, extensive research on the effects of EMF exposure on different biological targets (reproductive system, immune system, nervous system, etc.), through observational studies and experimental studies on different models, were carried out by laboratories all around the world.

The use of electromagnetic fields has focused on the creation of capillary networks for telecommunications and “wireless” connections: one of the major concerns regards the possible carcinogenic effects related to chronic EMF exposure at an intensity such as not to induce acute and/or perceptible effects [1].

In 2011, the International Agency for Research on Cancer (IARC) [2] classified RF-EMF as “possibly carcinogenic to humans”, allocating them to Group 2B of its classification system. The possible carcinogenic effects of RF-EMF have been investigated since the early 1980s on various animal (in vivo studies) and cellular (in vitro studies) models, evaluating both the direct onset of tumors and the alterations of tumor-related parameters. In this framework, in vivo studies have an important role in supporting the evidence derived from epidemiological studies aimed at evaluating the possible carcinogenic effects of RF exposure on the human population. The data of these studies, often contradictory, highlighted the need to carry out overall evaluations of the results, through international panels and reviews in order to perform a health risk assessment to support decision-makers and inform the general public.

To our knowledge this paper is the first attempt of a systematic review (with meta-analysis) on RF carcinogenic effects in in vivo studies.

RF-EMF animal studies on carcinogenesis cover a wide range of experimental situations, in terms of study design, exposure modality and biological endpoints. This peculiarity has ambivalent effect: on one hand, it is difficult to make a univocal classification of the studies and, consequently, to compare the results for a comprehensive analysis, on the other hand, the diversity of studies manages to cover a wide range of experiments, providing a reasonably good insight into the effects of RF-EMF exposure on carcinogenesis in laboratory animals.

Different exposure scenarios were employed in terms of frequency, dose of treatment, exposure modalities (i.e., full-body vs. localized exposure and restrained vs. free animals moving within large cages), duration and daily timing. Different frequencies were used, from a few hundred MHz up 3.7 GHz (for carcinogenesis studies), with different modulation schemes, (continuous wave (CW), pulsed, mobile signals). The most used frequencies were those of mobile communications and 2.45 GHz employed for Wireless Fidelity (Wi-Fi) systems and microwave ovens.

One of the main critical issues in the RF-EMF experimental in vivo studies is the assessment of the effective dose induced in the EMF exposed object/subject provided in terms of Specific Absorption Rate (SAR, W/kg) [3]. Moreover, the effect of RF-EMF exposure was studied both using RF-EMF alone and in synergy with other well-known carcinogens.

The aim of this review was to evaluate the effects of the RF-EMF in vivo exposure on tumor incidence at the systemic level or in different body organs/tissues.

## 2. Materials and Methods

The protocol of this systematic review is based on the guidance provided by the Cochrane Collaboration [4], the National Toxicology Program-Office of Health Assessment and Translation (NTP-OHAT) [5], and the guidelines “Preferred Reporting Items for Systematic Reviews and Meta-Analyzes” (PRISMA-P) [6]. This protocol is registered on PROSPERO [CRD42020191105] and published in a peer-review journal [7].

The protocol considers both carcinogenesis and co-carcinogenesis in in vivo studies; in this review, only the carcinogenesis analysis is discussed and presented, whereas the co-carcinogenesis analysis will be object of a next paper.

### 2.1. Eligibility Criteria

The review question was defined in terms of PECO (population, exposure, comparison, outcome):

- Population: rodents of both sexes, of all ages and species and of all genetic back-grounds (wild type, transgenic and tumor-prone animal models);
- Exposure: exposure to the electromagnetic field in the frequency range from 100 kHz to 300 GHz (all the modulations included), accurately characterized through dose assessment [8,9];
- Comparison: the “sham” sample, i.e., animals treated under conditions similar to those of exposed ones except for RF-EMF exposure, with particular reference to restraint conditions and stressing manipulations; papers describing experiments with cage control only or using the group exposed at the lowest dose level as a comparison were excluded;
- Outcome: the onset of neoplasms in laboratory animals exposed to RF, in terms of incidence of primary tumors; tumor incidence and survival were the main endpoints (outcome measures) on which this systematic review was focused;
- Articles reporting exclusively tumor-related parameters (i.e., genotoxicity, oxidative stress, etc.) were excluded from the analysis. Papers not written in English language and were not peer-reviewed and were not original (review, letters and comments) were excluded too.

### 2.2. Search Strategy

The search strategy of the primary works involved PubMed and EMF Portal as database sources, integrated with:

- the list of references of descriptive reviews on the same subject, published over the years or carried out by international panels of experts [2,10,11,12,13,14,15,16,17,18,19,20,21];
- the list of references of the selected papers;
- No limits were set on the year of publication;
- The query used for PubMed search and the criteria adopted on EMF Portal are attached to the protocol as supplement (suppl_1) [7].

Records identified from all the mentioned sources were imported into the EndNote X9 bibliographic management software. Its specific functions were used for both removing duplicates and the classification of works based on relevance and keywords.

The search strategy was peer-reviewed as part of the publication process of the protocol.

### 2.3. Selection Process

All potentially relevant articles were screened for eligibility in two stages: a first stage in which the articles were selected, on the basis of title and abstract, by three authors, and a second stage, in which the full texts of the remaining papers were independently reviewed by two groups of investigators, with each composed of one biologist and one expert in EMF dosimetry. Disagreements and technical uncertainties were discussed and resolved among review authors.

In order to define the dosimetry evaluation criteria and the possible problem of “publication bias” related to the historical period, an assessment of the years of publication was made before proceeding to the extraction data of the included articles.

### 2.4. Data extraction and Data Extraction Format

The data extraction form (Excel file) was defined and agreed upon before to start the analysis.

The extracted data included:

- Study design (number of experimental groups, control group(s), number of animals per group, randomization and blinding);
- Animal model: species, strain, sex and genotype of animals (wild type (WT)/transgenic);
- Exposure duration (LTE: long-term exposure, MTE: medium-term exposure, STE: short-term exposure);
- Timing of treatment (i.e., hours per day, days per week and total period);
- Exposure details (i.e., frequency, modulation, dose, exposure modalities in terms of whole body vs. localized exposure and restrained vs. freely moving animals type of exposure system);
- Primary outcome(s): all tumor-related outcome measures (incidence, tumor multiplicity, tumor volume, progression and survival) and numerical data were extracted from text, tables and figures (by using digital rulers) of each article, even if not all of this information was reported by all articles;
- Method to assess the endpoints;
- Data analysis and statistical evaluation;
- Authors, year of publication, title, journal;
- Information on animals died spontaneously of sacrificed for ethical reasons before the end of the exposure period.
  Three separate sheets were prepared in the file with the following data:
- General information on the experimental protocol: exposure characteristics, animal population, experimental protocol and endpoints (incidence, survival);
- Results;
- Risk of Bias (RoB).

We also extracted data on potential conflict of interest in all included studies.

The main purpose of this first data extraction scheme was to organize the information to carry out the RoB evaluations of the individual papers and to prepare a summary table (database for meta-analysis). In this table, each article was reported as many times as the number of treatment groups.

### 2.5. Classification of Tumors

A specific classification of the tumors was required because some of them can be both malignant and benign, and this is not always specified by the authors. The taxonomy adopted and the reasons behind it are reported in Table 1.

### 2.6. Risk of Bias (RoB) Evaluation

In order to evaluate the possible methodological limits, sources of error, which could influence the reliability of the summary result, a critical reading of all papers was carried out by two groups of reviewers independently to assess RoB following the criteria provided by the OHAT manual “Risk of Bias Rating Tool for Human and Animal Studies” [22]; for each paper, the following elements were evaluated:

1. Adequate randomization of administered dose or level of exposure, evaluating whether each animal had an equal chance of being assigned to a control or a treatment group;
2. Allocation of animals to treatment groups unknown to operators;
3. Evaluation of the experimental protocol or analysis of possible confounding variables not adequately identified and characterized;
4. Blinded treatment and analysis of groups of animals (blind or double-blind);
5. Evaluation of the exposure conditions, which had to be well defined and documented;
6. Use of standardized methods for determining the results (effects): specific and reliable tests and adequate statistical methodology;
7. Reporting of all expected outcomes;
8. Calculation of animal losses (attrition bias), due to death, during the experimental period, for reasons other than those foreseen by the experimental protocol;
9. Considering the relevance of the topic and knowing that many studies were funded by companies with significant commercial interests in the mobile telecommunications sector, it was decided to include in the Reporting Domain of the RoB, the possible Conflict of Interest as item 9.

Each of these 9 elements was evaluated according to the following scheme:

“**++** definitely low risk of bias” when there is evidence to exclude methodological errors in the study;

“**+** probably low risk of bias” when the evidence suggests that, even if methodological errors are present, their extent is such as not to influence the results of the study;

“**−** probably high risk of bias” there is evidence of possible errors or gaps in the definition of the element such as not to guarantee the quality of the results;

“**−−** definitely high risk of bias” there is evidence of methodological errors or serious gaps in the definition of the element that could have affected the results.

Three quality categories were defined (1: high quality, 2: intermediate quality, 3: low quality) where the papers were allocated on the basis of the evaluation of the 9 elements defined above; to define the category greater weight was given to items 3 (adequacy of the experimental protocol), 5 (adequate dosimetry) and 6 (reliability of the methods used to evaluate the outcome).

With regard to point 3 (“Evaluation of the experimental protocol or analysis of possible confounding variables not adequately identified and characterized”), it was decided to assign the judgment “**−** probably high risk of bias” or “**−−** definitely high risk of bias” to articles where the sham control group was shared by more than 3 treatment groups. In case of rare events, in fact, sharing one control group among more treated groups can be risky, because the presence of a “zero” or a “one” can be completely random and may “force” the overall result in one direction (increased risk) or another (reduced risk). In general, a control group larger than treated group (or case groups) is recommended.

Furthermore, it was decided to assign a “**−−**” to studies directly financed by companies (item 9).

### 2.7. Meta-Analysis: Strategy

All the data are reported as the number of events and non-events into the two groups of exposed and sham (2 × 2 table), so the meta-analysis was carried out by computing the Risk Difference (RD), the Odd Ratio (OR) and the Risk Ratio (RR), as effect size measure [23].

The meta-analysis performed is an Individual Participant Data (IPD) meta-analysis.

The Random Effects Model has been chosen to calculate the absolute and relative weight [24,25,26,27,28].

Parameters adopted to define homogeneity/heterogeneity and significance were I^2^, tau, z-value, *p*-value (significance *p* < 0.01).

From the summary table, a table for each organ/tumor was created with the most relevant information such as:

- the SAR value (without uncertainty);
- the type of exposure duration (LTE-longer than 52 weeks, MTE-longer than 9 weeks, STE);
- the number of exposed animals with tumor (treated incidence), the total number of animals in the exposed sample, the number of sham animals with tumor (sham incidence), the total number of the animals in the sham sample; in the papers employing both sexes, the incidence data in males and females were added together, so analysis by sex was not performed;
- the animal type and the genetic background (WT/prone).

Animal species and genetic background were used for the subgroup analysis: this assessment was performed in order to validate the hypothesis of homogeneity of the included studies; dose and “exposure time” were used for the regression analysis. The “exposure time” data is expressed in terms of total hours of exposure, and it was obtained as the simple product of the number of exposure hours per day by the number of actual exposure days.

Meta-Essentials tool (version 1.5) [29] was chosen and used to carry out the meta-analysis. The tool consists of a set of Excel workbooks (one for each type of independent variable), prepared by a team from the Rotterdam School of Management, Erasmus University, The Netherlands, under an ERIM Support Program and licensed under Creative Commons Attribution-Non-Commercial-ShareAlike 4.0 International ([29,30]). The tool (the folders of our interest, binary data) is complete and totally transparent of operations and algorithms providing the possibility of making changes and validation.

The results are shown reporting the summary effect size RR, with the relative variability limits, the significance, the forest plot and the funnel plot for publication bias.

If the funnel plot, upon visual inspection, showed that more imprecise studies with non-harmful effects were missing, this was considered an indication of publication bias.

### 2.8. Quality Assessment (Confidence Ratings and Evidence of Health Effects)

To evaluate the quality of evidence, that is, the confidence in the estimates of observed effect, we primarily applied the guidance from NTP-OHAT [5]. The assessment was performed for the entire body of evidence by each outcome; possible disagreements and uncertainties were discussed among review authors and the agreement was reached by consensus. We started from a “high quality” grade, a general feature for randomized in vivo studies [5], and six items were considered to degrade this quality of evidence: (i) experimental design, (ii) Risk of Bias, (iii) inconsistency, (iv), indirectness of evidence, (v) imprecision and (vi) publication bias. Within each of the relevant domains, concern for quality of evidence was assessed using the ratings: “none”, this evaluation leads to no lowering of the rating; “serious”, that results in a lowering of the quality by one level; and “very serious”, that results in a lowering of the quality by two levels. Two items—consistency between species and presence of a dose response—were considered to upgrade the quality of evidence. The quality was classified according to the OHAT categories as high, moderate, low or very low. Finally, the Evidence of Health Effects was evaluated according to the same tool.

## 3. Results

### 3.1. General Description of the Selected Carcinogenicity studies

A total of 294 primary articles (114 articles obtained from EMF Portal, 112 obtained from PubMed and 166 obtained from other sources) were selected and uploaded to EndNote after removing duplicate records. The databases were last consulted in April 2022.

The first screening by title and abstract was performed according to the defined exclusion criteria.

The technical reports of the National Toxicology Program (NTP) [31,32] on carcinogenesis effects of RF exposure on rats and mice, respectively, were included, even if not published yet; these reports, released in 2018, although controversial, are considered among the most complete studies currently available on the impact of RF exposure on carcinogenesis [21].

After the first screening, a total of 237 papers were excluded, and the remaining 57 were examined using full-text analysis. A further 11 papers were excluded for the following reasons:

- Missed or incomplete EMF dosimetry (*n* = 3; [33,34,35]);
- Absence of the sham control group (*n* = 3; [36,37,38]);
- Studies based on animals in which tumor cells are implanted (“implanted tumor”) before exposure to RF in order to evaluate the effects on the development of neoplasms. The aim of this review is to investigate the genesis of each type of tumors not their development (*n* = 3; [39,40,41]);
- Absence of specific data (*n* = 2; [42,43]). In particular, [42] examines the onset and growth of neoplasms exclusively by palpation and the data are only given in terms of cumulative tumor appearance without specifying the organ of onset and [43] provides data on various histological parameters not strictly related to the onset of neoplasms, where only the lack of onset of leucosis (a tumor process affecting the progenitor cells of leukocytes) is observed, and tumor incidence is not reported.

After this last selection, a total of 46 articles were eligible: 23 papers were carcinogenesis studies, 19 were co-carcinogenesis studies and 4 analyzed both carcinogenesis and co-carcinogenesis.

Most of these articles (35) have been published within the decade 2000–2010, when the European Community decided to fund many projects on this topic in the Framework Programs; this opportunity favored the standardization of the exposure protocols (and the exposure systems) and therefore the quality and the homogeneity of the studies.

**Figure 1:**
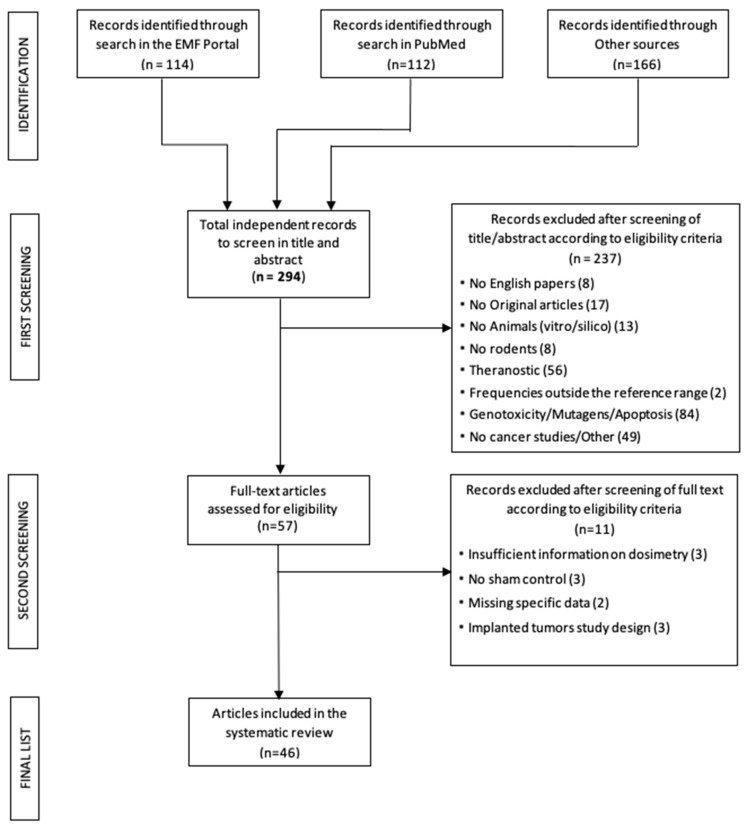
Flowchart of search strategy and selection process.

Given that the data extraction proceeded by separating the carcinogenesis articles from those of co-carcinogenesis, the papers dealing with both treatments were included in both groups composed of 27 and 23 articles, respectively. The flow chart with the results of the bibliography acquisition process is shown in Figure 1.

The papers concerning the co-carcinogenesis alone [44,45,46,47,48,49,50,51,52,53,54,55,56,57,58,59,60] have been successively excluded because they will be the subject of another article.

Regarding carcinogenesis, the selected 27 papers reported the results of 66 different treatment groups: most of the papers (14) analyzed only one treatment group, [61,62,63,64,65,66,67,68,69,70,71,72,73,74], five articles reported two treatment groups [75,76,77,78,79], two articles had three treatment groups [80,81], one article had four treatment groups [82], four articles had six treatment groups [31,32,83,84] and, finally, one article had eight treatment groups [85]. A summary of the most relevant information of the 66 treated-sham control comparisons in terms of populations, exposure and outcomes is reported in Appendix A.

**Figure 2:**
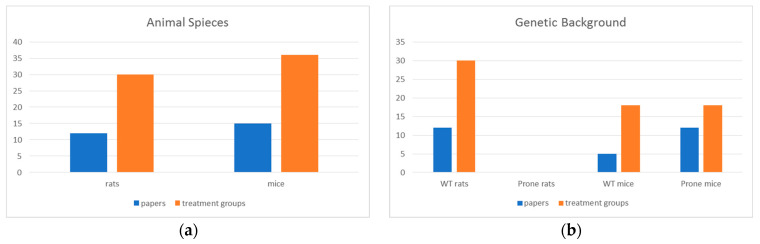
Distribution of papers and treatment groups for the (**a**) animal species and (**b**) genetic background.

Regarding the type of animals (POPULATION) employed in the selected papers, a total of 12 papers (30 treatment groups) described experiments performed on rats, and the remaining 15 papers (36 treatment groups) used mice (Figure 2a).

All studies using rats were carried out on ‘wild type’ strains (Fisher, Wistar or Sprague Dawley); whereas, with regards the experiments on mice, 10 papers (18 treatment groups) reported experiments performed on prone mice, 3 papers (18 treatment groups) showed experiments on ‘wild type’ mice and 2 papers (10 treatment groups) reported experiments on both ‘wild type’ and prone animals (Figure 2b).

A total of 14 papers (42 treatment groups) described experiments on animals of both sexes, 11 papers (22 treatment groups) only on female animals, whereas two papers (two treatment groups) only on males.

**Figure 3:**
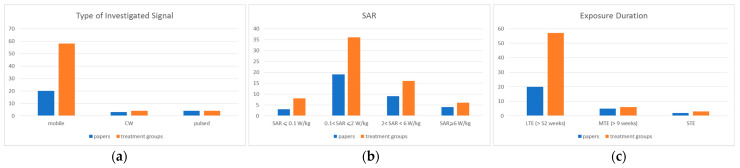
Distribution of papers and treatment groups for the (**a**) used frequency, (**b**) SAR dose and (**c**) duration.

Regarding the characteristics of the used electromagnetic signals (EXPOSURE), a total of 20 papers (58 treatment groups) reported experiments on exposure to cell phone frequencies (800–900 MHz GSM, 800–900 MHz CDMA, 1700–1900 MHz DCS, 1700–2000 MHz UMTS/CDMA); 3 papers (four treatment groups) reported experiments with exposures at 2450 MHz continuous wave (CW); and 4 papers presented exposures to pulsed signals with different characteristics. Chou et al. [63] performed experiments at 2450 MHz (pulse of 10 μs, 800 pps), de Seze et al. [64] carried out experiments at 3700 MHz (pulses of 2.5 ns, 100 pps), Jauchem et al. [67] reported exposures to an Ultra-Wide Band signal (pulses of 2.5 ns, 1 kHz) and Toler et al. [74] presented exposures at 435 MHz (1 μs, 1 kHz) (Figure 3a). Moreover only five papers (eight treatment groups) presented experiments with localized exposures of the animals’ head (all with SAR values lower than 2 W/kg); the remaining papers (22 articles and 58 treatment groups) concerned experiments with whole body exposures.

Regarding the dose, SAR values ≤ 0.1 W/kg were used in 3 papers (8 treatment groups), SAR values in the interval 0.1 < SAR ≤ 2 W/kg were used in 19 papers (36 treatment groups), SAR values in the interval 2 < SAR < 6 W/kg were used in 9 papers (16 treatment groups) and, finally, SAR values greater than 6 W/kg were used in 4 papers (6 treatment groups) (Figure 3b). 

Regarding the duration of exposure, 20 papers (57 treatment groups) reported LTE experiments, 5 papers (6 treatment groups) reported MTE experiments and only 2 papers (3 treatment groups) exhibited very short exposures (Figure 3c).

Moreover 15 papers (38 treatment groups) reported experiments with daily exposures less than 4 h, 11 papers (26 treatment groups) reported experiments with daily exposures greater than 12 h and only 1 paper (2 treatment groups) reported experiments with daily 6-h exposures.

Regarding the type of assessed OUTCOME measures, all papers reported the incidence data provided in terms of the number of animals developing cancer; 22 papers (50 treatment groups) also reported the survival data.

### 3.2. RoB of the Selected Papers

The results of the overall assessment of the RoB and the quality category of the carcinogenesis studies included in the analysis are reported in Table 2.

### 3.3. Incidence Analyses

A table for each organ/tumor was created from the summary table and, in agreement with most authors, some organs have been grouped according to the anatomical system: eye, harderian gland, ear, nose and mouth have been inserted into the sensorial system; prostate, testicles, glans and epididymis have been inserted into the male uro-genital system; uterus, ovaries and clitoris have been inserted into the female uro-genital system; brain and cranial nerves have been inserted into the central nervous system (CNS). These tables, containing “raw” data, are shown in S1.

Furthermore, considering the importance of the CNS and the brain, the latter was also analyzed separately; in addition, a detailed analysis of brain tumor type was carried out using data from the studies that detailed their typing.

After the definition of the groups for the meta-analysis on the basis of the organ/tumor, three more papers were excluded for the substantial difference in the treatments with respect to the other papers:

- de Seze et al. [64]: 3.7 GHz pulsed signal administered for two 8-min intervals per day, 5 times per week for a total of 8 weeks;
- Jauchem et al. [67]: UWB signal administered for 12 min/week for a total of 12 weeks;
- Saran et al. [77]: 900 MHz GSM modulation signal, administered for two 30 min/day for 5 days.
- Furthermore, the article by Jin et al. [68] was also excluded from the meta-analysis as it only reports inflammatory phenomena and does not detect the onset of tumors.
- A qualitative descriptive analysis of these papers is separately reported.

In S2 (one figure for each organ/tumor), all the data of the meta-analysis are shown: the “raw” incidence data, the effect size measure RR of each treated-sham comparison, with the relative variability limits, and the relative significance; the forest plot and the funnel plot for publication bias.

All the summary information on the possible increase in the risk of the onset of malignant and benign tumors, consequent to RF exposure, is reported in Table 3 and Table 4, and in terms of RR and RD, evaluated organ by organ.

In addition to the results, the following data, for each sample (organ/tissue), is reported: number of treated-sham comparisons (number of elements in the sample, column 2 of Table 3 and Table 4), number of papers from which the studies were extracted (column 3) and the ratio between the total number of exposed animals and the total number of sham animals (column 5). It was decided to insert in column 4 the information, supplementary to column 5, regarding the number of papers where more than two treatment groups share the same sham; moreover, the maximum number of treatment groups with the same sham was reported. For example, in the first line of Table 3 (Adrenals), out of 24 elements (treated-sham comparisons) extracted from 8 papers, four papers present different treatment groups (up to six) compared with a single sham: for 24 treatment groups (for a total of 3538 animals) there are only eight sham groups (for a total of 1166 animals). As can be seen in Table 3 and Table 4, the number of sham animals was always much lower than the number of exposed animals.

There was a substantial agreement of the results obtained with both RR and RD summary effects. The non-significance of almost all results was observed with the exception of: CNS, brain, heart and intestine for malignant tumors, CNS, brain, male uro-genital system and kidney for benign tumors.

It should also be noted the apparent discrepancy in the bone marrow results was that despite both RR and RD agree on a risk decrease, RD would be statistically significant, unlike RR. Another apparent discrepancy is the result of the leukemia sample where RR and RD disagree and moreover the significance results only for RD (this disagreement is due to the algorithm for the calculation of RD, when one “zero” value is present in the comparison).

The data relating to malignant tumors of heart and brain, which showed significance in the results of the meta-analysis, are analyzed in detail in the Section 3.3.1.

A definitive synthesis of the incidence of (all) tumors in relation to RF exposure, providing a “sum” data for malignant and a “sum” data for benign tumors was not possible. Despite a fair homogeneity in the exposure conditions (duration longer than one year, total-body exposure and SAR levels below 4 W/kg) indeed, most studies have considered only a few organs, and this has determined a lack of homogeneity regarding the endpoint evaluation. The “sum” data obtained from the only studies presenting data in all organs, would be more conclusive but affected by a high “attrition bias”, so the incomplete input of the data would make them incorrect.

#### 3.3.1. Heart and CNS/Brain Analysis

**Figure 4:**
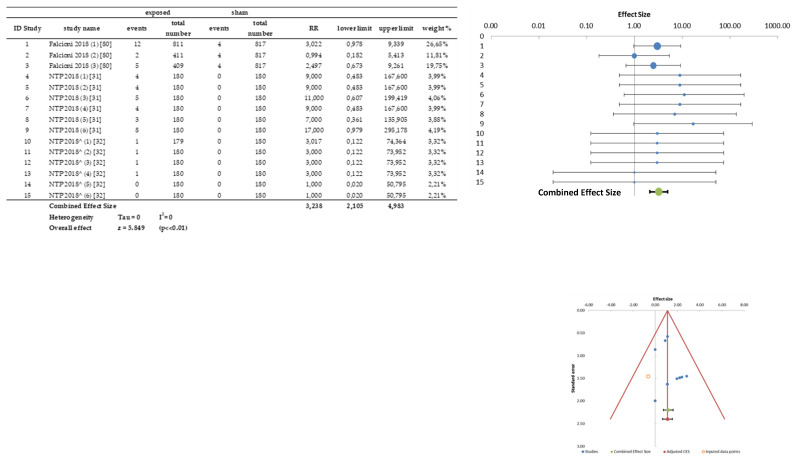
Raw data, forest plot and funnel plot of the heart malignant tumors (NTP 2018^ identifies the NTP mice study).

**Figure 5:**
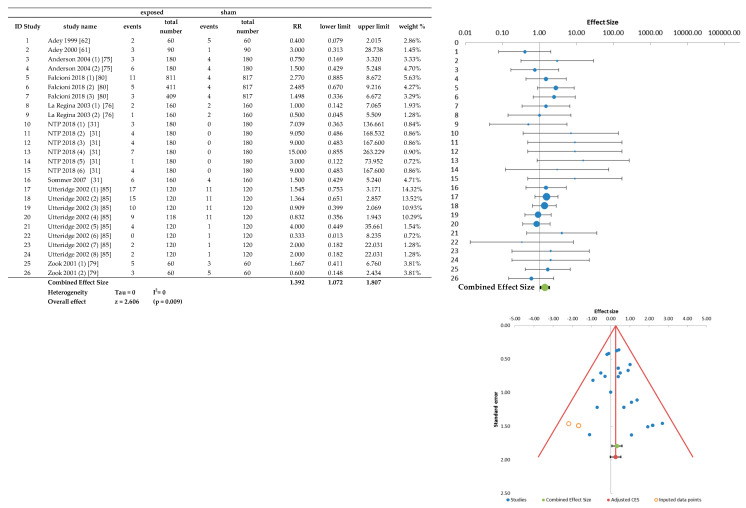
Forest plot and raw data of the brain malignant tumors.

The results of meta-analysis on tumor incidence in the samples of heart and brain (CNS results are similar and showed in Figure S2.5 in File S2), complete with forest plot and funnel plot, are shown in Figure 4 and Figure 5, respectively.

Considering the importance of the brain as a target organ and the significance of the increased risk, it was decided to perform the analysis by tumor type according to the classification provided by most of the authors. Two papers ([72,85]) did not provide information on the type of tumors; the other ones (7 out of 9 papers, for a total of 17 comparisons out of a total of 26) provided classification criteria that allowed the grouping of tumors (both malignant and benign) into tumors of the glia and meninges. These samples were analyzed by considering separately the incidences of malignant glia and meninges tumors (including the granular cell tumor), and benign meninges tumors (this latter sample coincides with all the benign tumors, Figure S2.27 in in File S2). The results of this detailed analysis are shown in Table 5.

**Figure 6:**
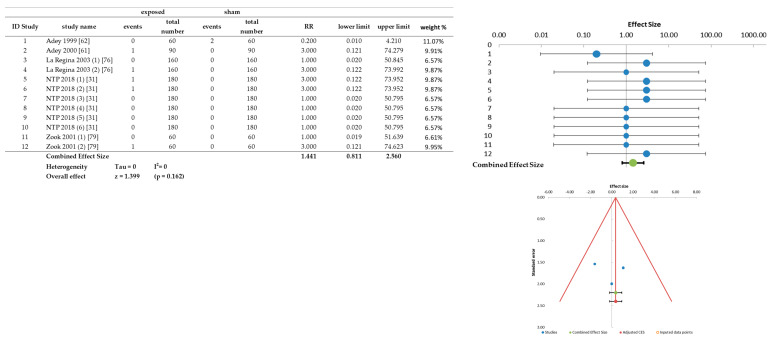
Forest plot and raw data of the spinal cord malignant tumors.

As a further study, an analysis of only malignant tumors of the spinal cord (no benign tumors were evidenced in this tissue) was also performed. The results are shown in the Figure 6. In this tissue the combined RR is 1.441 with no significance (*p* = 0.162).

#### 3.3.2. Subgroup Analysis

The subgroup analysis was performed for the covariates, species and genetic background, because the samples had a fair degree of homogeneity in all the other elements. The results are presented in Table 6 and Table 7, for malignant and benign tumors, respectively. For each sample, the “combined” RR with the relative p-between are reported. The subgroup analysis was carried out only for samples in which treated-sham comparisons derived from more than two articles.

There are no significant differences in the genetic background comparison for either malignant or benign tumors, whereas in the comparison between species, breast and spleen malignant tumors and skin benign tumors show significant differences.

#### 3.3.3. Regression Analysis

In Table 8, the results of the regression analysis for the covariates dose and exposure time are reported for malignant tumors, whereas the results for benign tumors are shown in Table 9. The results are related to the RR variable and include: the coefficient of the regression line, the *p*-value relative and the R^2^ (%). Samples with significant regression are in bold (Leukemia and Mammary).

The regression analysis results do not provide useful elements to define a dose–effect or duration–effect relationship in any of the analyzed samples. The exposure times significances reported in the Table 8 and Table 9, (see malignant breast cancer, leukemia and benign adrenal glands tumors (adrenals and thyroid) are attributable to the high range of variability of the variable “duration of exposure” (546–25.000 h). Within this range, however, the occurrences are concentrated in a few repeated values. These results, indeed, do not show any correlation with the data of Summary Effect Size (and the relative significance).

**Figure 7:**
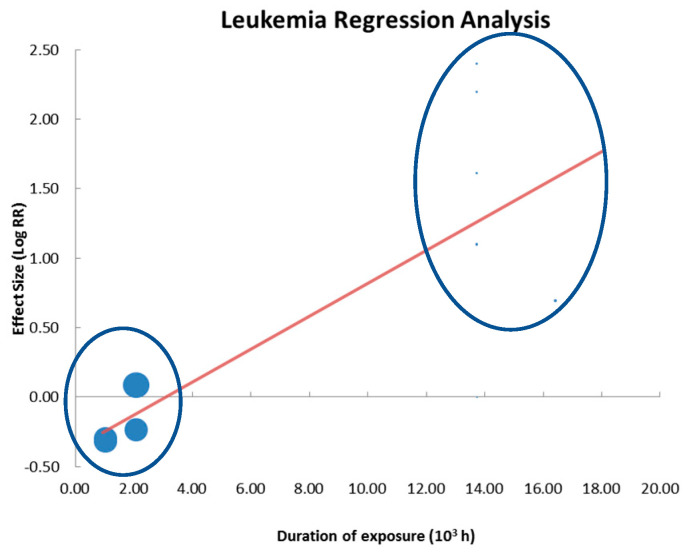
Regression analysis graph for the Leukemia sample.

To clarify this concept, the regression line of Leukemia sample is shown (Figure 7): four elements have a maximum duration of a little more than 2000 h, the other 13 elements have durations greater than 13,700 h, there are no intermediate “duration values”. These data confirm the high discontinuity of exposure times and significantly reduce confidence in the obtained result.

### 3.4. Survival Analysis

It was not possible to carry out the survival analysis by periods, or cumulative survival analysis, due to the lack of time intervals common to all studies. The number of live animals at the end of the exposure period was defined as variable for the survival; such variable refers to different periods due to the different exposure durations (from a few weeks up to 2 years). Studies that observed animals to death without reporting the survival data at the end of exposure were excluded from the analysis. An exception was [64], whose experimental protocol provided for an exposure time of a few minutes/day for 8 weeks (to ultra-broadband signals of high intensity), followed by an observation period of 2 years; in this case, the survival variable refers to the end of the observation period.

**Figure 8:**
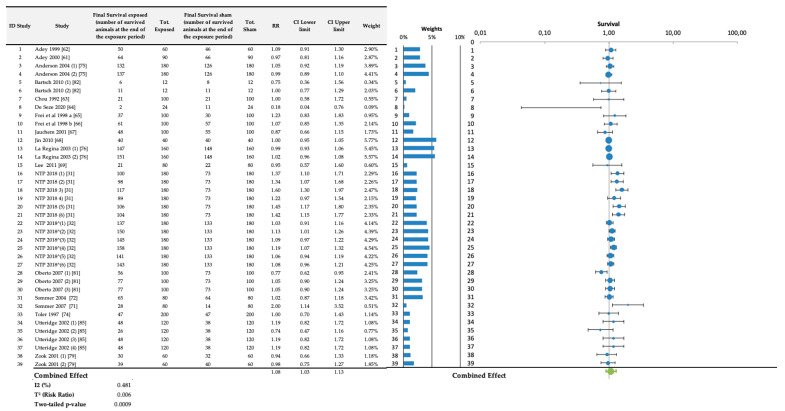
Survival analysis results deriving from the data extracted from the selected carcinogenesis studies (NTP 2018^ identifies the NTP mice study).

The overall meta-analysis was performed on 39 treated-sham comparisons and the RR was obtained with the same procedure used for the incidence analysis. The table of the meta-analysis results and the related forest plot are shown in Figure 8. In this case, the overall RR value is 1.08 (1.03–1.14).

### 3.5. Qualitative Summary of the Excluded Works from the Meta-Analysis

Jin et al. [68] exposed male and female Sprague Dawley (SD) rats to combined CDMA 848.5 MHz and WCDMA signals at 1950 MHz with a combined SAR of 4 W/kg. The histological examination of most of the organs revealed no neoplasms, with the exception of a couple of benign tumors. The authors argue that there are no exposure effects on carcinogenesis and survival.

Saran et al. [77] exposed sensitive to X-ray transgenic mice (Patched1 heterozygous knockout mice) at 900 MHz at 0.4 W/kg. Previous results from the same group [86] demonstrated that these animals, when exposed to X-ray, in the first days of life, have a significant incidence of medulloblastoma and rhabdomyosarcoma. Although exposure to RF occurred during the aforementioned sensitivity time window, the authors found no effects on carcinogenesis and survival.

Jauchem et al. [67] exposed C3H/HeJ female mice, a susceptible strain developing mammary tumors, to an ultra-broadband signal at a SAR of 0.0098 W/kg. The authors found no effects on the onset of breast tumors and on survival.

De Seze et al. [64] exposed male SD rats to ultra-wideband signals to 3.7 GHz at a SAR of 0.83 W/kg. The protocol included two exposures of 8 min/day, 5 days/week, for a total of 8 weeks; animals were observed up to 2 years of life. The authors observed a reduction in the survival of exposed animals (4 months over 2 years) and a significantly higher incidence of subcutaneous tumors in exposed animals compared to sham. This effect could be related to the peak high SAR value (> 3 MW/kg) administered via a continuous pulse train of 2.5 ns for 2 intervals per day of 8 min each.

### 3.6. Quality Assessment (Confidence Ratings and Evidence of Health Effects)

According to the protocol [7], the evaluation of the quality of evidence was performed starting from a “high quality” grade and the eight items as defined in the Methods paragraph were considered; the following Table 10 and Table 11 were obtained for malignant and benign tumors, respectively.

## 4. Discussion

In this systematic review, we summarized the current knowledge on carcinogenesis in laboratory animals exposed to electromagnetic fields, in the frequency range 100 kHz–300 GHz. For this purpose, we carried out a qualitative descriptive analysis of the 27 articles that were considered eligible on the basis of the exclusion criteria defined in the protocol [7]. It was feasible to carry out a meta-analysis of the possible increase in the risk of the onset of tumors on 23 of the eligible papers.

The in-depth reading of the papers with more than one treatment group highlighted that the number of sham animals is always lower than the number of exposed animals; therefore, in our analysis, sham control was shared with multiple treatment groups. As already pointed out, this practice, although very common in in vivo studies, determines an over estimation of events/non-events in sham controls which can lead to unreliable results in a meta-analysis aimed at assessing the risk of rare events, as in this review. In any case, a substantial agreement of the results obtained with both RR and RD variables was found with the exception of leukemia for malignant tumors. Almost all results were non-significant with the exception of: CNS, brain, heart and intestine for malignant tumors, and CNS, brain, male uro-genital system and kidney for benign tumors.

Based on these considerations, the samples that showed significant results in the meta-analysis deserve a detailed investigation. The significant results for benign brain tumors derive from nine treated-sham comparisons extracted from only two papers ([31,80]), with each showing two brain benign tumors in the sham groups out of 817 animals and 180 animals, respectively. In the treated-sham comparisons, these values are repeated three and six times, respectively, against the incidence values in the treated groups that ranged from three to eight in [80] and from one to five in [31] (Table S1.27 in File S1).

Regarding the significance found in the onset of benign male uro-genital system and kidneys tumors, the presence of “zeros” in some exposed groups [31,32] is compared with the presence of three and four tumors in the sham groups for the male uro-genital system and with the presence of two and six tumors in the sham groups for the kidney (Tables S1.29 and S1.32 in File S1), leading to factitious decreases in risk. The same consideration can be applied to the reduction in the risk of developing malignant tumors in the intestine where the combined RR is 0.585 (0.399–0.857) with *p* < 0.01 (Table S1.11 and Figure S2.11 in File S1).

The increased incidence of malignant heart tumors risk (Figure 4) was an expected result due to the data on heart schwannoma in [31,32,80], but not reported by any of the selected papers. In this meta-analysis, in each organ/tumor sample, only included were the papers reporting the presence of one tumor in at least one of the comparison groups: many papers examined heart histologically without finding any primary tumor ([63,65,66,67,74,75,76,79,81,83,84,85]) and their results were not reported in the heart sample. As a consequence, the significance of the results of the heart sample can be attributed only to the data of [31] study (six treated-sham comparisons with a single sham) and of [80] (three treated-sham comparisons with only one sham of higher number); whereas, the incidences found in [32] (values of zero and one in all groups) can be attributed to randomness. The summary effect size measure is strongly affected by the presence of the “zero” in the two sham groups of [31,32] (repeated 12 times), although the authors consider it in line with the incidences found in the Historical Controls (0–2%). Moreover, [80] shows a relevant incidence data (12 vs. 4) only in animals exposed to the lowest SAR level (0.001 W/kg) among all the considered exposure doses. In addition, it should be considered that data of this sample derive from only two studies and, therefore, the hypothesis of independence of the elements is much more labile than other samples (organs/tumor).

It should also be highlighted that:

- The studies of [31,32] are not peer-reviewed and [80], by admission of the authors themselves, published only the data relating to heart to support the results of [31], stating to publish the complete data (on the other organs) at a later time. For these reasons the heart sample results affected by publication bias;
- In this sample, a dose-effect response is not demonstrated, despite a very wide range of variability in SAR levels (from 0.001 W/kg to 6 W/kg), (see Table 8);
- The authors of [31,80] report no statistical significance of their results in the overall assessment (collecting both sexes data, i.e., excluding the differences between the sexes).
- The Authors of [31], regarding the increase of malignant tumor onset in the heart of male rats, declares: “In many cases isolated non-neoplastic or neoplastic lesion increases occurred in single or lower exposure groups, lacked a clear exposure response, or incidences were similar to incidences seen in control groups in past NTP studies. This reduced the confidence that these lesion increases were attributable to the cell phone RFR exposure.”

All these considerations contribute to considerably reducing the suspicion of a direct correlation between exposure to RF and the increased risk of developing cardiac neoplasms.

The CNS and brain samples represent the target of greatest interest in all the carcinogenesis papers of this review (as many as 20 articles out of 27 concern the effects of mobile telephony). The slight increased incidence risk of malignant tumor in the CNS (RR = 1.405) and in the brain alone (RR = 1.392) was an unexpected result, as no in vivo carcinogenicity study has ever found a statistically significant incidence data for brain and CNS tumors.

The significance of the data of this sample (9 papers, 26 treated-sham comparisons) is due to the weak positivity of most of the comparisons (Figure 5): 18 comparisons present a relative risk in a positive direction (RR > 1, corresponding to an increase in risk), whereas only eight have a RR ≤ 1.

Moreover, in this case, the presence of the “zero” in the sham group of the [31] study (repeated six times) strongly influences the overall result so that, by removing the six treated-sham comparisons of [31] from the samples (both brain and CNS), the value of the summary RR decreases and loses the statistical significance. In this new condition, the brain sample, for example, consists of 20 comparisons, coming from eight papers, and has an RR = 1.267 [1.003–1.603] with *p* = 0.034.

## 5. Conclusions

This systematic review analyzed the experimental data extracted from 27 eligible articles regarding the onset of neoplasms in laboratory rodents exposed to EMF-RF; a quantitative analysis (meta-analysis) was conducted on 23 papers. Each study was examined for possible methodological limits and the RoB was evaluated.

A total of 25 organs/tumors were analyzed for malignant tumors and 16 for benign tumors to assess the confidence in the body of evidence of the carcinogenic effects. Starting from a “high quality” grade, a general feature for randomized in vivo studies [5], all items underwent a quality downgrade due to “serious” or “very serious” limitations in the experimental design, mainly caused by a low number of animals in sham groups. A further downgrade was determined by the classification of all studies as “some concerns” for bias, even without taking into account the conflict of interest.

The results obtained after subgrouping analysis by species (rats vs. mice) allowed an upgrade of the certainty of the evidence for many types of malignant and benign tumors. The lack of a dose–response relationship in all the analyzed samples did not allow for further upgrades.

Overall, these evaluations have determined a confidence rating from very low (heart sample for malignant tumors and CNS sample for benign ones) to moderate, resulting in inadequate or insufficient health evidence for a definitive assessment of the association between EMF-RF exposure and carcinogenesis in vivo.

This lack of certainty in the conclusions mainly derives from a very cautious GRADE approach, which does not appear entirely justified in this case given that the considered articles present a good homogeneity, both in the methods and in the results, providing adequate answers for the aims of this study. In this regard, it should be considered that, although in recent years the use of systematic reviews has been extended to experimental laboratory studies, the main guidelines [4,5] were developed considering the clinical trials. The different approach between clinical and laboratory works has highlighted some methodological difficulties for the application of grade procedures, which could be better analyzed in order to improve the guidelines for the future systematic reviews on animal studies.

Furthermore, it should be considered that the inclusion of only English-language papers may have represented a limitation of this systematic review.

In conclusion, the inadequate/insufficient health evidence found does not allow this systematic review to give additional information for the integration of present regulatory frameworks. Otherwise, this review updates the state of the art of research on in vivo RF-EMF experiments related to carcinogenesis and, for future research in this field, it emphasizes the need of an appropriate experimental design that takes into account the animal number and the sample number used for the sham control groups.

Future work will be the update of this review as required in [4]; in fact, the question of this review is of continuing importance to decision makers and the availability of new data or new methods would have a meaningful impact on the review findings. Moreover, a review update provides an opportunity for the scope, eligibility criteria and methods used in the review to be revised.

## Figures and Tables

**Table 1:** Tumor classification (malignant tumors are omitted).

**Tumors Always Classified as Benign**
Teratoma (ovarian), Stromal Polyp, Neurilemmoma, Cholangioma, Keratoacanthoma, Squamous Cell Papilloma, Pilomatrixoma, Cystadenoma, Leiomyoma, Hibernoma, Fibroma, Fibroadenoma, Lipoma.
**Tumors (Malignant or Benign) Classified as Malignant by Precautionary Approach, Unless the Authors Have not Indicated Otherwise**
Hemangioma, Granular Cell Tumor, Renal Mesenchymal Tumor, Hepatoblastoma, Nephroblastoma.
**Tumors (Rare with Low Percentage of Cases of Malignancy) Classified as Benign, Unless the Authors Have not Indicated Otherwise**
Pheochromocytoma, Interstitial Cell Tumor.

**Table 2:** RoB of the carcinogenesis studies and their related quality category.

Paper	Item Score (−, −−, +, ++)									Quality Assessment (1–3)
	1	2	3	4	5	6	7	8	9	
Adey 1999 [62]	+	+	+	+	−	++	++	++	−	2
Adey 2000 [61]	+	+	+	+	−	++	++	++	−	2
Anderson 2004 [75]	++	++	++	++	++	++	++	++	−−	2
Bartsch 2010 [45]	+	+	+	++	++	+	++	++	−−	2
Chou 1992 [63]	++	++	+	++	++	++	++	++	++	1
De Seze 2020 [64]	−	−	+	−	+	++	++	−−	−	3
Falcioni 2018 [80]	+	++	++	++	+	++	++	+	++	1
Frei 1998 a [65]	++	++	++	+	++	++	++	+	+	1
Frei 1998 b [66]	++	++	++	+	++	++	++	+	+	1
Jauchem 2001 [67]	+	++	++	++	++	++	++	++	+	1
Jin 2010 [68]	++	++	+	++	++	++	++	++	++	1
La Regina 2003 [76]	+	+	+	++	++	++	++	++	−−	2
Lee 2011 [69]	+	++	++	+	++	++	++	++	−	1
NTP Rats 2018 [31]	+	++	−−	++	++	++	++	++	++	2
NTP Topi 2018 [32]	+	++	−−	++	++	++	++	++	++	2
Oberto 2007 [81]	++	++	+	++	++	++	++	++	++	1
Repacholi 1997 [70]	+	−	−−	++	+	++	++	++	+	2
Saran 2007 [77]	++	++	+	++	++	++	+	−	++	1
Smith 2007 [83]	++	++	+	++	++	++	++	++	−	1
Sommer 2004 [72]	++	+	+	++	++	++	++	++	++	1
Sommer 2007 [71]	++	−	++	++	++	++	++	++	++	1
Szimigielski 1982 [78]	−−	−−	++	−−	+	++	++	−	++	3
Tillmann 2007 [84]	++	++	+	++	++	++	++	++	−	1
Tillmann 2010 [73]	++	++	++	++	++	++	++	++	++	1
Toler 1993 [74]	++	−	+	++	++	++	++	+	++	1
Utteridge 2002 [85]	++	++	−	++	++	++	+	++	++	1
Zook 2001 [79]	+	−−	+	++	++	++	++	++	−−	2

1. Randomized exposure level; 2. Allocation concealment of study groups; 3. Evaluation in the study design or analysis of possible important confounding and modifying variables; 4. Blinding of research personnel; 5. Confidence in the exposure characterization (dosimetry); 6. Confidence in the outcome assessment; 7. All measured outcomes reported; 8. Attrition/exclusion rate; 9. Possible conflicts of interest. The meaning of + and – is specified M&M section

**Table 3:** Summary table of meta-analysis results carried out by “organ/malignant tumor”: for each sample both RR and RD with the relative limits of variability (CI 95%) and *p*-value are reported. Statistically significant results are highlighted in bold.

Malignant Tumors	Number of Included ’Treated-Sham Comparisons’	Number of Papers	(*1)	Number of Exposed Animals/Number of sham Animals	Risk Ratio (RR)				Risk Difference (RD)			
Organ or Tumor					Summary Effect Size (RR)	Lower Limit RR	Upper Limit RR	Two tailed *p* Value RR	Summary Effect Size (RD)	Lower Limit RD	Upper Limit RD	Two tailed *p* Value RD
Adrenal Glands	24	8	4/6	3538/1166	1.016	0.684	1.510	0.93200	−0.004	−0.010	0.001	0.07940
Bladder	15	4	2/6	2495/770	0.904	0.540	1.512	0.67200	−0.001	−0.004	0.002	0.36310
Histiocytic Sarcoma	26	8	4/6	3724/1155	0.979	0.735	1.305	0.88000	0.003	−0.001	0.010	0.17330
Bone Marrow	7	2	1/6	1175/279	0.558	0.304	1.024	0.01900	−0.007	−0.012	−0.003	0.00004
**CNS (brain and spinal cord)**	**26**	**9**	**4/6**	**4779/2007**	**1.405**	**1.070**	**1.840**	**0.00900**	**0.009**	**0.004**	**0.013**	**0.00020**
**Brain**	**26**	**9**	**4/6**	**4779/2007**	**1.392**	**1.072**	**1.807**	**0.00900**	**0.008**	**0.003**	**0.012**	**0.00020**
Sensorial System	20	4	4/6	3034/712	1.028	0.681	1.552	0.89000	0.001	−0.002	0.010	0.46240
Male Uro-Genital System	10	3	1/6	880/260	1.756	1.034	2.982	0.01600	0.006	−0.020	0.010	0.08390
Female Uro-Genital System	32	9	5/6	2354/822	0.882	0.720	1.070	0.20000	0.001	−0.010	0.010	0.84000
**Heart**	**15**	**3**	**3/6**	**3790/1117**	**3.238**	**2.105**	**4.983**	**0.00000**	**0.008**	**0.003**	**0.010**	**0.00080**
**Intestine**	**14**	**3**	**2/6**	**2376/505**	**0.585**	**0.399**	**0.857**	**0.00200**	**−0.008**	**−0.012**	**−0.004**	**0.00000**
Kidneys (Renal System)	14	3	2/6	2460/519	0.949	0.572	1.573	0.82100	−0.004	−0.010	0.000	0.02050
Leukemia	17	5	2/6	2939/800	*0.884*	0.738	1.059	0.14700	0.006	**0.002**	0.010	**0.00270**
Liver	25	8	5/6	3497/1086	0.958	0.861	1.066	0.40300	0.002	−0.002	0.010	0.39480
Lung	23	7	4/6	3394/1031	0.886	0.769	1.021	0.07700	0.000	−0.003	0.004	0.88440
Lymphoma	41	15	7/6	5645/2184	1.003	0.969	1.038	0.86800	−0.002	−0.008	0.003	0.35600
Mammary Tumors	29	10	4/6	3362/1274	1.034	0.839	1.275	0.74200	−0.001	−0.010	0.002	0.92080
Mesenteric Lymph Nodes	16	4	4/6	2241/631	0.822	0.436	1.551	0.51000	−0.002	−0.010	−0.006	0.31490
Pancreas	18	3	4/6	2713/555	1.167	0.941	1.448	0.13100	−0.001	−0.010	−0.006	0.48810
Pituitary Gland	25	7	4/6	3193/887	0.981	0.774	1.243	0.86600	0.002	−0.001	0.005	0.08840
Skin	15	5	2/6	2456/658	0.755	0.566	1.008	0.03700	−0.009	−0.020	0.002	0.07520
Spleen	14	4	2/6	2282/506	1.067	0.354	3.223	0.89900	0.001	−0.012	0.013	0.91300
Stomach	7	2	1/6	1179/280	0.777	0.321	1.881	0.48500	−0.003	−0.008	0.001	0.07100
Thymus	14	3	3/6	1938/527	0.912	0.582	1.429	0.65800	0.000	−0.003	0.004	0.97940
Thyroid	26	7	5/6	3790/1094	1.229	0.963	1.567	0.08100	−0.001	−0.005	0.002	0.45800

(*1) Number of papers with single sham shared with more than two studies/Max number of treated groups sharing the same sham. The number of independent sham groups is generally equal to the number of papers; only 3 papers use 2 sham groups vs more than six exposed groups.

**Table 4:** Summary table of meta-analysis results carried out by “organ/benign tumor”: for each sample both RR and RD with the relative limits of variability (CI 95%) and *p*-value are reported. Statistically significant results are highlighted in bold.

Benign Tumors	Number of Included ’Treated-Sham Comparisons’	Number of Papers	(*1)	Number of Exposed Animals/Number of Sham Animals	Risk Ratio (RR)				Risk Difference (RD)			
Organ or Tumor					Summary Effect Size (RR)	Lower Limit RR	Upper Limit RR	Two Tailed *p* Value RR	Summary Effect Size (RD)	Lower Limit RD	Upper Limit RD	Two Tailed *p* Value RD
Adrenal Glands	26	8	5/6	3656/1107	1.433	1.088	1.888	0.00700	0.010	−0.004	0.025	0.14500
**Brain**	**9**	**2**	**2/6**	**2711/997**	**2.163**	**1.371**	**3.411**	**0.00010**	**0.006**	**0.003**	**0.009**	**0.00001**
Sensorial System	15	3	4/6	1976/479	1.007	0.754	1.345	0.95900	0.010	−0.007	0.026	0.19600
**Male Uro-Genital System**	**17**	**5**	**2/6**	**1523/451**	**0.951**	**0.915**	**0.987**	**0.00500**	**−0.018**	**−0.030**	**−0.004**	**0.00770**
Female Uro-Genital System	23	8	6/6	2219/822	1.021	0.888	1.174	0.76300	0.011	−0.010	0.030	0.17790
Intestine	12	2	2/6	2055/345	1.137	0.488	2.651	0.73900	0.000	−0.010	0.050	0.91840
**Kidneys (Renal System)**	**14**	**4**	**2/6**	**2296/513**	**0.515**	**0.374**	**0.708**	**0.00001**	**−0.010**	**−0.015**	**−0.005**	**0.00001**
Liver	28	10	5/6	3915/1346	1.044	0.945	1.153	0.37500	−0.004	−0.010	0.010	0.38470
Lung	24	7	5/6	3314/911	0.994	0.870	1.135	0.92200	−0.004	−0.008	0.000	0.03000
Mammary Tumors	26	8	4/6	2975/929	0.993	0.930	1.060	0.82800	0.015	−0.010	0.040	0.30250
Pancreas	23	7	4/6	3333/1007	1.082	0.906	1.293	0.35900	0.004	−0.001	0.010	0.13170
Pituitary Gland	31	8	7/6	4105/1195	1.033	0.972	1.097	0.27800	0.005	−0.004	0.013	0.26500
Skin	13	3	2/6	2260/460	1.140	0.797	1.630	0.42600	0.003	−0.005	0.012	0.42600
Stomach	14	4	2/6	2330/548	0.719	0.525	0.985	0.02300	−0.003	−0.005	−0.001	0.00030
Thymus	15	3	2/6	2104/527	0.830	0.645	1.067	0.11100	−0.002	−0.005	0.002	0.29070
Thyroid	24	7	4/6	3574/1080	1.139	0.979	1.327	0.07600	0.005	0.000	0.010	0.02680

(*1) Number of papers with single sham shared with more than two studies/Max number of treated groups sharing the same sham. The number of independent sham groups is generally equal to the number of papers; only 3 papers use 2 sham groups vs more than six exposed groups.

**Table 5:** Summary table of the meta-analysis results carried out on glia and meninges malignant and benign tumors. RR values with the relative limits of variability (CI 95%) and *p*-value are reported for each sample.

Brain Malignant Tumors	Risk Ratio								
Organ or Tumor	Number of Included Studies/Number of papers	(*1)	Number of Exposed Animals/Number of Sham Animals	Summary Effect Size (RR)	Two Tailed *p* Value RR	Lower Limit RR	Upper Limit RR	Tau_square_value	I_square (%)
Glia tumors	16/6	2/6	3601/1487	2.63	0.000004	1.69	4.11	0	0
Meninges tumors	15/6	2/6	3541/1487	1.60	0.018000	1.05	2.45	0	0
Meninges benign tumors	15/6	2/6	2711/997	2.16	0.000100	1.37	3.41	0	0

(*1) Number of papers with single sham shared with more than two studies/Max number of treated groups sharing the same sham. The number of independent sham groups is generally equal to the number of papers; only 3 papers use 2 sham groups vs more than six exposed groups.

**Table 6:** Summary table of the subgroup analysis on malignant tumors: the selected covariates for relevance are the species (mice vs. rats) and the genetic background (prone vs. WT). Bold items represent the results with statistical significance.

Malignant Tumors	Mice vs Rats			Prone vs WT		
Organ or Tumor	Number of Studies with Mice/Number of Studies with Rats	Combined Summary Effect RR	*p* between	Number of Studies with Prone/Number of Studies with WT	Combined Summary Effect RR	*p* between
Adrenal Glands	15/9	0.90	0.1130	3/21	1.02	0.9270
Bladder	6/9	1.03	0.4260	WT only	-	-
Histiocytic Sarcoma	17/9	1.48	0.0940	4/22	0.98	0.8370
Bone Marrow	mice only	-	-	WT only	-	-
CNS (brain and spinal cord)	9/17	1.37	0.2780	5/21	1.45	0.6720
Brain	9/17	1.35	0.3150	5/21	1.42	0.6420
Sensorial System	12/8	1.44	0.0810	WT only	-	-
Male Uro-Genital System	rats only	-	-	WT only	-	-
Female Uro-Genital System	18/14	0.85	0.0920	WT only	-	-
Heart	6/9	2.72	0.5850	WT only	-	-
Intestine	6/8	0.56	0.5940	WT only	-	-
Kidneys (Renal System)	6/8	0.66	0.0720	WT only	-	-
Leukemia	6/11	1.59	0.0810	WT only	-	-
Liver	18/7	1.20	0.3000	5/20	0.96	0.9280
Lung	15/8	0.89	0.9320	2/21	0.95	0.6960
Lymphoma	30/11	0.95	0.4010	13/28	1.03	0.7100
**Mammary Tumors**	**11/18**	**1.01**	**0.0080**	5/24	0.99	0.0270
Mesenteric Lymph Nodes	7/9	0.80	0.7900	1/15	-	-
Pancreas	6/12	1.16	0.8090	WT only	-	-
Pituitary Gland	9/16	1.43	0.1120	WT only	-	-
Skin	8/7	0.80	0.2310	2/13	0.69	0.7050
**Spleen**	**8/6**	**0.96**	**0.0000**	1/13	-	-
Stomach	rats only	-	-	WT only	-	-
Thymus	rats only	-	-	WT only	-	-
Thyroid	9/17	0.93	0.1750	3/23	1.30	0.9280

**Table 7:** Summary table of the subgroup analysis on benign tumors: the selected covariates for relevance are the species (mice vs. rats) and the genetic background (prone vs. WT) Bold items represent the results with statistical significance.

Benign Tumors	Mice vs Rats			Prone vs WT		
Organ or Tumor	Number of Studies with Mice/Number of Studies with Rats	Combined Summary Effect RR	*p* between	Number of Studies with Mice/Number of Studies with Rats	Combined Summary Effect RR	*p* between
Adrenal Glands	17/9	1.33	0.0550	5/21	1.07	0.0960
Brain	Only Rats	-	-	Only WT	-	-
Sensorial System	Only mice	-	-	3/12	2.43	0.0380
Male Uro-Genital System	6/11	0.65	0.0590	Only WT	-	-
Female Uro-Genital System	15/14	1.06	0.0800	3/26	1.02	0.9270
Intestine	6/6	1.12	0.0100	Only WT	-	
Kidneys (Renal System)	7/7	0.5	0.7370	Only WT	-	-
Liver	19/9	0.9	0.0650	6/22	1.02	0.9960
Lung	18/6	0.99	0.6470	5/19	0.95	0.3330
Mammary Tumors	7/17	1.26	0.3530	1/23	-	-
Pancreas	8/15	1.68	0.0750	2/21	1.54	0.4420
Pituitary Gland	16/15	1.12	0.0830	4/27	1.29	0.0730
**Skin**	**6/7**	**0.66**	**0.0010**	Only WT	-	-
Stomach	Only Rats	-	-	Only WT	-	-
Thymus	3/12	1.03	0.5390	Only WT	-	-
Thyroid	7/17	1.74	0.1330	1/23	-	-

**Table 8:** Summary table of linear regression analysis for malignant tumors: the selected covariates are the dose (wbSAR) and the overall exposure time. Bold items represent the results with statistical significance.

Malignant	SAR			Time of Exposure		
Organ or Tumor	β-Moderator	*p* Value	R^2^ %	β-Moderator	*p* Value	R^2^ %
Adrenal Glands	−0.45	0.0700	20.65	0.18	0.4600	3.36
Bladder	−0.29	0.2880	8.62	−0.24	0.6300	5.80
Histiocytic Sarcoma	−0.09	0.7050	0.76	−0.28	0.2300	7.60
Bone Marrow	0.05	0.9500	0.20	-	-	-
CNS (brain and spinal cord)	−0.05	0.8220	0.22	0.53	0.0110	28.60
Brain	−0.26	0.2250	6.97	0.55	0.0120	29.80
Sensorial System	−0.09	0.7900	0.74	0.03	0.9200	0.10
Male Uro-Genital System	0.06	0.9200	0.35	−0.13	0.8200	1.75
Female Uro-Genital System	−0.16	0.4400	2.49	−0.40	0.0600	14.90
Heart	0.29	0.4670	8.27	−0.56	0.1530	31.90
Intestine	−0.08	0.8630	0.57	−0.03	0.9500	0.08
Kidneys (Renal System)	−0.22	0.5710	5.00	−0.36	0.3700	12.60
Leukemia	0.52	0.0350	26.79	**0.84**	**0.0010**	**70.90**
Liver	−0.04	0.8720	0.18	0.13	0.6300	1.67
Lung	0.39	0.2490	15.03	0.54	0.1000	29.20
Lymphoma	0.25	0.1710	6.07	−0.14	0.4400	1.90
Mammary Tumors	0.36	0.0600	13.00	**−0.68**	**0.0000**	**45.90**
Mesenteric Lymph Nodes	−0.07	0.8290	0.54	0.08	0.8000	0.70
Pancreas	−0.31	0.3960	9.56	0.01	0.9700	0.01
Pituitary Gland	0.39	0.2150	15.18	−0.50	0.1000	25.80
Skin	−0.20	0.5040	3.95	0.21	0.4700	4.50
Spleen	0.24	0.3950	5.59	−0.16	0.5560	2.70
Stomach	0.30	0.6400	9.00	0.57	0.3700	32.80
Thymus	−0.28	0.3000	8.00	−0.20	0.7000	4.20
Thyroid	−0.10	0.7400	0.90	−0.07	0.8000	0.50

**Table 9:** Summary table of linear regression analysis for benign tumors: the selected covariates are the dose (wbSAR) and the overall exposure time. Bold items represent the results with statistical significance.

Benign	SAR			Time of Exposure		
Organ or Tumor	β-Moderator	*p* Value	R^2^ %	β-Moderator	*p* Value	R^2^ %
Adrenal Glands	0.26	0.1950	6.60	0.64	0.0002	40.33
Brain	−0.46	0.3930	20.81	0.73	0.1720	53.31
Sensorial System	0.17	0.5210	2.98	0.16	0.5470	2.63
Male Uro-Genital System	−0.34	0.2200	11.39	−0.31	0.2660	9.37
Female Uro-Genital System	0.32	0.1500	10.10	0.10	0.6520	0.99
Intestine	0.39	0.2900	15.16	-	-	-
Kidneys (Renal System)	−0.45	0.3460	20.03	0.36	0.4540	12.60
Liver	0.02	0.9220	0.04	0.40	0.0260	15.94
Lung	0.27	0.3940	7.07	0.45	0.1460	20.57
Mammary Tumors	−0.89	0.1000	23.70	−0.45	0.1340	19.90
Pancreas	−0.24	0.2920	5.69	0.48	0.0350	22.80
Pituitary Gland	−0.25	0.3090	6.40	−0.17	0.4870	3.00
Skin	−0.44	0.0940	19.51	0.37	0.1670	13.70
Stomach	0.13	0.8420	1.80	0.16	0.8070	2.70
Thymus	−0.08	0.8630	0.66	0.12	0.7110	1.50
Thyroid	0.52	0.0290	27.11	**0.64**	**0.0080**	**40.30**

**Table 10:** Quality Assessment for the results on malignant tumors in selected organs.

	N. of studies (groups/papers)	Design	RoB	Inconsistency	Indirectness of Evidence	Imprecision	Publication Bias	Exposed	Comparison	Relative Effect RR (CI 95%)	Consistency between Species	Dose Response	Quality of Evidence	Health Evidence
Adrenal Glands	24/8	Serious (−1)	Some corcern (−1)	No (I^2^ = 0%)	No	No	No	3538	1166	1.01 (0.68–1.51)	Yes (+1)	No	Moderate	Inadequate
Bladder	15/4	Serious (−1)	Some corcern (−1)	No (I^2^ = 0%)	No	No	No	2495	770	0.9 (0.54–1.512)	Yes (+1)	No	Moderate	Inadequate
Histiocytic Sarcoma	26/8	Serious (−1)	Some corcern (−1)	No (I^2^ = 0%)	No	No	No	3724	1155	0.79 (0.41–1.53)	Yes (+1)	No	Moderate	Inadequate
Bone Marrow	7/2	Serious (−1)	Some corcern (−1)	No (I^2^ = 0%)	No	No	No	1175	279	0.56 (0.3–1.02)	No	No	Low	Inadequate
CNS (brain and spinal cord)	26/9	Very Serious (−2)	Some corcern (−1)	No (I^2^ = 0%)	No	No	No	4779	2007	1.40 (1.07–1.84)	Yes (+1)	No	Low	Low
Brain	26/9	Very Serious (−2)	Some corcern (−1)	No (I^2^ = 0%)	No	No	No	4779	2007	1.39 (1.07–1.81)	Yes (+1)	No	Low	Low
Sensorial system	20/4	Serious (−1)	Some corcern (−1)	No (I^2^ = 0%)	No	No	No	3034	712	1.03 (0.68–1.55)	Yes (+1)	No	Moderate	Inadequate
Male Uro-Genital System	10/3	Serious (−1)	Some corcern (−1)	No (I^2^ = 0%)	No	No	No	880	260	1.76 (1.03-2.98)	No	No	Low	Inadequate
Female Uro-Genital System	32/9	Serious (−1)	Some corcern (−1)	No (I^2^ = 0%)	No	No	No	2354	822	0.92 (0.72–1.17)	Yes (+1)	No	Moderate	Inadequate
Heart	15/3	Serious (−1)	Some corcern (−1)	No (I^2^ = 0%)	No	No	Yes (−2)	3790	1117	3.24 (2.11–4.98)	Yes (+1)	No	Very Low	Inadequate
Intestine	14/3	Very Serious (−2)	Some corcern (−1)	No (I^2^ = 0%)	No	No	No	2376	505	0.59 (0.4–0.86)	Yes (+1)	No	Low	Low
Kidneys (Renal System)	14/3	Serious (−1)	Some corcern (−1)	No (I^2^ = 0%)	No	No	No	2460	519	0.95 (0.57–1.57)	Yes (+1)	No	Moderate	Inadequate
Leukemia	17/5	Serious (−1)	Some corcern (−1)	No (I^2^ = 3.9%)	No	No	No	2939	800	0.88 (0.74–1.06)	Yes (+1)	No	Moderate	Inadequate
Liver	25/8	Serious (−1)	Some corcern (−1)	No (I^2^ = 0%)	No	No	No	3497	1086	0.96 (0.86–1.07)	Yes (+1)	No	Moderate	Inadequate
Lung	23/7	Serious (−1)	Some corcern (−1)	No (I^2^ = 0%)	No	No	No	3394	1031	0.89 (0.77–1.02)	Yes (+1)	No	Moderate	Inadequate
Lymphoma	41/15	Serious (−1)	Some corcern (−1)	No (I^2^ = 0%)	No	No	No	5645	2184	1.04 (1.03–1.05)	Yes (+1)	No	Moderate	Inadequate
Mammary Tumors	29/10	Serious (−1)	Some corcern (−1)	No (I^2^ = 43.9%)	No	No	No	3362	1274	0.99 (0.55–1.80)	No	No	Low	Inadequate
Mesenteric Lymph Nodes	16/4	Serious (−1)	Some corcern (−1)	No (I^2^ = 0%)	No	No	No	2241	631	0.82 (0.44–1.55)	Yes (+1)	No	Moderate	Inadequate
Pancreas	18/3	Serious (−1)	Some corcern (−1)	No (I^2^ = 0%)	No	No	No	2713	555	1.17 (0.94–1.45)	Yes (+1)	No	Moderate	Inadequate
Pituitary Gland	25/7	Serious (−1)	Some corcern (−1)	No (I^2^ = 0%)	No	No	No	3193	887	0.99 (0.77–1.24)	Yes (+1)	No	Moderate	Inadequate
Skin	15/5	Serious (−1)	Some corcern (−1)	No (I^2^ = 0%)	No	No	No	2456	658	0.75 (0.57–1.01)	Yes (+1)	No	Moderate	Inadequate
Spleen	14/4	Serious (−1)	Some corcern (−1)	No (I^2^ = 44.5%)	No	Yes (−1)	No	2282	506	1.07 (0.35–3.22)	Yes (+1)	No	Low	Inadequate
Stomach	7/2	Serious (−1)	Some corcern (−1)	No (I^2^ = 0%)	No	No	No	1179	280	0.78 (0.32–1.88)	No	No	Low	Inadequate
Thymus	14/3	Serious (−1)	Some corcern (−1)	No (I^2^ = 0%)	No	No	No	1938	527	0.91 (0.58–1.43)	No	No	Low	Inadequate
Thyroid	26/7	Serious (−1)	Some corcern (−1)	No (I^2^ = 0%)	No	No	No	3790	1094	1.23 (0.96–1.58)	Yes (+1)	No	Moderate	Inadequate

Design: Serious: most information is from ++ and +, but there are a few − (because in each sample there is a high number of shared sham groups); Very Serious: the shared sham group (from NTP study) introduce an anomal data repeated as many times as the number of treated groups. RoB: Some Corcern: some studies show "−" in some relevant item; Conflict of interest item is not considered. Inconsistency: No if I^2^ < 50%, Serious (−1) I^2^ > 50% (up to 75%). Indirectness of Evidence: No: (most information is from wild type rodents). Imprecision: No, because of the high number of animals and because the boundaries of the CI of the pooled effect size are on the same side of the null value or the ratio; between the CI interval and the null value (RR) is less than 110%. Publication Bias: Yes: the authors declare the publication of incomplete data due to the fact that their data support the original data of the NTP study; No other study publishes data on the heart despite having analyzed it.

**Table 11:** Quality Assessment for the results on benign tumors in selected organs.

	N. of Studies (Groups/Papers)	Design	RoB	Inconsistency	Indirectness of Evidence	Imprecision	Publication Bias	Exposed	Comparison	Relative Effect RR (CI 95%)	Consistency between Species	Dose Response	Quality of Evidence	Health Evidence
Adrenal Glands	26/8	Serious (−1)	Some corcern (−1)	I^2^ = 28.08	No	No	No	3656	1107	1.43 (1.09–1.89)	Yes (+1)	No	Moderate	Inadequate
CNS/Brain	9/2	Very Serious (−2)	Some corcern (−1)	I^2^ = 0	No	No	Yes (−1)	2711	997	2.16 (1.37–3.41)	Yes (+1)	No	Very Low	Inadequate
Sensorial system	15/3	Serious (−1)	Some corcern (−1)	I^2^ = 0	No	No	No	1976	479	1.01 (0.75–1.35)	Yes (+1)	No	Moderate	Inadequate
Male Uro-Genital System	17/5	Serious (−1)	Some corcern (−1)	I^2^ = 0	No	No	No	1523	451	0.95 (0.92–0.99)	No	No	Low	Inadequate
Female Uro-Genital System	23/8	Serious (−1)	Some corcern (−1)	I^2^ = 0	No	No	No	2219	822	1.06 (0.98–1.15)	Yes (+1)	No	Moderate	Inadequate
Intestine	12/2	Very Serious (−2)	Some corcern (−1)	I^2^ = 0	No	Yes (−1)	No	2055	345	1.14 (0.48–2.65)	Yes (+1)	No	Low	Low
Kidneys (Renal System)	14/4	Serious (−1)	Some corcern (−1)	I^2^ = 0	No	No	No	2296	513	0.52 (0.37–0.71)	Yes (+1)	No	Moderate	Inadequate
Liver	28/10	Serious (−1)	Some corcern (−1)	I^2^ = 13.02	No	No	No	3915	1346	1.04 (0.95–1.15)	Yes (+1)	No	Moderate	Inadequate
Lung	24/7	Serious (−1)	Some corcern (−1)	I^2^ = 0	No	No	No	3314	911	0.95 (0.69–1.30)	Yes (+1)	No	Moderate	Inadequate
Mammary Tumors	26/8	Serious (−1)	Some corcern (−1)	I^2^ = 40	No	No	No	2975	929	1.13 (0.98–1.29)	No	No	Low	Inadequate
Pancreas	23/7	Serious (−1)	Some corcern (−1)	I^2^ = 0	No	Yes (−1)	No	3333	1007	1.54 (0.71–3.35)	Yes (+1)	No	Moderate	Inadequate
Pituitary Gland	31/8	Serious (−1)	Some corcern (−1)	I^2^ = 0	No	No	No	4105	1195	1.03 (0.97–1.1)	Yes (+1)	No	Moderate	Inadequate
Skin	13/3	Serious (−1)	Some corcern (−1)	I^2^ = 21.32	No	No	No	2260	460	1.14 (0.8–1.63)	Yes (+1)	No	Moderate	Inadequate
Stomach	14/4	Serious (−1)	Some corcern (−1)	I^2^ = 0	No	No	No	2330	548	0.72 (0.52–0.98)	No	No	Low	Inadequate
Thymus	15/3	Serious (−1)	Some corcern (−1)	I^2^ = 0	No	No	No	2104	527	0.83 (0.65–1.1)	No	No	Low	Inadequate
Thyroid	24/7	Serious (−1)	Some corcern (−1)	I^2^ = 0	No	No	No	3574	1080	1.14 (0.98–1.33)	Yes (+1)	No	Moderate	Inadequate

Design: Serious: most information is from ++ and +, but there are a few − (because in each sample there is a high number of shared sham groups); Very Serious: the shared sham group (from NTP study) introduce an anomal data repeated as many times as the number of treated groups. RoB: Some Corcern: some studies show "−" in some relevant item; Conflict of interest item is not considered. Inconsistency: No if I^2^ < 50%, Serious (−1) I^2^ > 50% (up to 75%). Indirectness of Evidence: No: (most information is from wild type rodents). Imprecision: No, because of the high number of animals and because the boundaries of the CI of the pooled effect size are on the same side of the null value or the ratio; between the CI interval and the null value (RR) is less than 110%. Publication Bias: Yes: the authors declare the publication of incomplete data due to the fact that their data support the original data of the NTP study; No other study publishes data on the heart despite having analyzed it.

## Data Availability

Authors have the access to all scientific databases, in order to collect all relevant papers for the systematic review.

## Supplementary Materials

The following supporting information can be downloaded at: https://www.mdpi.com/article/10.3390/ijerph20032071/s1. File S1: Raw data in terms of malignant and benign tumor incidence for each considered organ/tumor; File S2: Incidence data and results of meta-analysis. Malignant tumors from Figure S2.1 to Figure S2.25; benign tumors from Figure S2.26 to Figure S2.41.

## Appendix A

**Table A1:** Summary of the most relevant information of the 27 papers (66 treated-sham control comparisons) in terms of populations, exposure and outcomes.

ID Paper [ref]	ID Treatment Group	Author Year (Treat-Group Number)	RoB (Quality Category)	Species	Strain	Prone/ WT	Sex	Exposure Starts in Utero	Number of Animals per Group	Frequency	Modulation/ Platform	SAR (W/Kg)	wb SAR/ Local SAR	Duration (w)	Timing (h/d, d/w)	Organ	Type of Tumor (Mal/Ben)	Outcome Measure	Note
1 [62]	1	Adey 1999	2	Rats	F344	WT	M+F	Y	60	836 MHz	TDMA	1–1.60	local	LTE (94)	2 h/d 4d/w	CNS	Malignant tumors	Incidence/ survival	SAR values related to growth
2 [61]	2	Adey 2000	2	Rats	F344	WT	M+F	Y	90	836 MHz	TDMA	0.74–1.60	local	LTE (96)	3 h/d 4d/w	CNS	Malignant tumors	Incidence/ survival	SAR values related to growth
3 [75]	3	Anderson 2004 (1)	2	Rats	F344	WT	M+F	Y	180	1600 MHz	Iridium (QPSK)	0.16	local	LTE (104)	4 h/d 7d/w	Multiple	Malignant and benign tumors	Incidence/ survival	There is a cage control group, sham is shared
	4	Anderson 2004 (2)	2	Rats	F344	WT	M+F	Y	180	1600 MHz	Iridium (QPSK)	1.6	local	LTE (104)	5 h/d 7d/w	Multiple	Malignant and benign tumors	Incidence/ survival	
4 [82]	5	Bartsch 2010 (1)	2	Rats	SD	WT	F	N	12	900 MHz	GSM	0.038–0.08	wb	LTE (101)	24 h/d 7d/w	Pituitary	Malignant tumors	Incidence/ survival	SAR values related to growth
	6	Bartsch 2010 (2)	2	Rats	SD	WT	F	N	12	900 MHz	GSM	0.038–0.08	wb	LTE (75)	24 h/d 7d/w	Pituitary	Malignant tumors	Incidence/ survival	SAR values related to growth
	7	Bartsch 2010 (3)	2	Rats	SD	WT	F	N	30	900 MHz	GSM	0.038–0.08	wb	LTE (150)	24 h/d 7d/w	Multiple	Malignant tumors	Incidence/ survival	SAR values related to growth
	8	Bartsch 2010 (4)	2	Rats	SD	WT	F	N	30	900 MHz	GSM	0.038–0.08	wb	LTE (151)	24 h/d 7d/w	Multiple	Malignant tumors	Incidence/ survival	SAR values related to growth
5 [63]	9	Chou 1992	1	Rats	SD	WT	M	N	100	2450 MHz	pulse: 10 μs; 800 pps	0.15–0.4	wb	LTE (109)	21,5 h/d 7d/w	Multiple	Malignant and benign tumors	Incidence/ survival	SAR values related to growth
6 [64]	10	De Seze 2020	3	Rats	SD	WT	M	N	24	3700 MHz	pulse: 2,5 ns; 100 pps	0.83	wb	STE (8)	2 x 8 min 5d/w	Multiple	Malignant and benign tumors	Incidence/ survival	
7 [80]	11	Falcioni 2018 (1)	1	Rats	SD	WT	M+F	N	811/817	1800 MHz	DCS	0.001	wb	LTE (152)	19 h/d 7d/w	Multiple	Malignant and benign tumors	Incidence/ survival	reported heart and brain only, sham is shared
	12	Falcioni 2018 (2)	1	Rats	SD	WT	M+F	N	411/817	1800 MHz	DCS	0.03	wb	LTE (152)	20 h/d 7d/w	Multiple	Malignant and benign tumors	Incidence/ survival	
	13	Falcioni 2018 (3)	1	Rats	SD	WT	M+F	N	409/817	1800 MHz	DCS	0.1	wb	LTE (152)	21 h/d 7d/w	Multiple	Malignant and benign tumors	Incidence/ survival	
8 [65]	14	Frei et al 1998 a	1	Mice	C3H/HeJ	Prone	F	N	100	2450 MHz	CW	0.3	wb	LTE (78)	20 h/d 7d/w	Multiple	Malignant and benign tumors	Incidence/ survival	
9 [66]	15	Frei et al 1998 b	1	Mice	C3H/HeJ	Prone	F	N	100	2450 MHz	CW	1	wb	LTE (78)	20 h/d 7d/w	Multiple	Malignant and benign tumors	Incidence/ survival	
10 [67]	16	Jauchem 2001	1	Mice	C3H/HeJ	Prone	F	N	100	UWB	pulse width 2.5 ns; PRF: 1KHz	0.01	wb	MTE (12)	2 m 1d/w	Multiple	Malignant and benign tumors	Incidence/ survival	duration: 12 m/w × 12 w (short duration and single pulse signal)
11 [68]	17	Jin 2010	1	Rats	SD	WT	M+F	N	40	849 MHz 1950 MHz	CDMA+WCDMA	4	wb	LTE (52)	45 m 5d/w	Multiple	Benign tumors	Incidence/ survival	No tumors found, except 2 benign and some non-neoplastic lesions
12 [76]	18	La Regina 2003 (1)	2	Rats	F344	WT	M+F	N	160	835.62 MHz	FDMA	1.3 ± 0.5 (in brain)	local	LTE (104)	4 h/d 5d/w	Multiple	Malignant and benign tumors	Incidence/ survival	sham is shared
	19	La Regina 2003 (2)	2	Rats	F344	WT	M+F	N	160	847.74 MHz	CDMA	1.3 ± 0.5 (in brain)	local	LTE (104)	4 h/d 5d/w	Multiple	Malignant and benign tumors	Incidence/ survival	
13 [69]	20	Lee 2011	1	Mice	AKR/J mice	Prone	M+F	N	80	849 MHz 1950 MHz	CDMA+WCDMA	6	wb	MTE (42)	45 min 5d/w	Lymphoma	Malignant tumors	Incidence/ survival	
14 [31]	21	NTP 2018 (1)	2	Rats	SD	WT	M+F	Y	180	900 MHz	GSM	1.5	wb	LTE (107)	18^h^ 20’/7 (10’ ON / 10’ OFF)	Multiple	Malignant and benign tumors	Incidence/ survival	only 1 sham vs 6 exposed groups; data about "historical" control groups reported in Annexes
	22	NTP 2018 (2)	2	Rats	SD	WT	M+F	Y	180	900 MHz	GSM	3	wb	LTE (107)	18^h^ 20’/7 (10’ ON / 10’ OFF)	Multiple	Malignant and benign tumors	Incidence/ survival	
	23	NTP 2018 (3)	2	Rats	SD	WT	M+F	Y	180	900 MHz	GSM	6	wb	LTE (107)	18^h^ 20’/7 (10’ ON / 10’ OFF)	Multiple	Malignant and benign tumors	Incidence/ survival	
	24	NTP 2018 (4)	2	Rats	SD	WT	M+F	Y	180	900 MHz	CDMA	1.5	wb	LTE (107)	18^h^ 20’/7 (10’ ON / 10’ OFF)	Multiple	Malignant and benign tumors	Incidence/ survival	
	25	NTP 2018 (5)	2	Rats	SD	WT	M+F	Y	180	900 MHz	CDMA	3	wb	LTE (107)	18^h^ 20’/7 (10’ ON / 10’ OFF)	Multiple	Malignant and benign tumors	Incidence/ survival	
	26	NTP 2018 (6)	2	Rats	SD	WT	M+F	Y	180	900 MHz	CDMA	6	wb	LTE (107)	18^h^ 20’/7 (10’ ON / 10’ OFF)	Multiple	Malignant and benign tumors	Incidence/ survival	
15 [32]	27	NTP 2018^ (1) (*)	2	Mice	B6C3F1/N	WT	M+F	N	180	1900 MHz	DCS	2.5	wb	LTE (107)	18^h^ 20’/7 (10’ ON / 10’ OFF)	Multiple	Malignant and benign tumors	Incidence/ survival	only 1 sham vs 6 exposed groups; data about "historical" control groups reported in Annexes
	28	NTP 2018^ (2)	2	Mice	B6C3F1/N	WT	M+F	N	180	1900 MHz	DCS	5	wb	LTE (107)	18^h^ 20’/7 (10’ ON / 10’ OFF)	Multiple	Malignant and benign tumors	Incidence/ survival	
	29	NTP 2018^ (3)	2	Mice	B6C3F1/N	WT	M+F	N	180	1900 MHz	DCS	10	wb	LTE (107)	18^h^ 20’/7 (10’ ON / 10’ OFF)	Multiple	Malignant and benign tumors	Incidence/ survival	
	30	NTP 2018^ (4)	2	Mice	B6C3F1/N	WT	M+F	N	180	1900 MHz	CDMA	2.5	wb	LTE (107)	18^h^ 20’/7 (10’ ON / 10’ OFF)	Multiple	Malignant and benign tumors	Incidence/ survival	
	31	NTP 2018^ (5)	2	Mice	B6C3F1/N	WT	M+F	N	180	1900 MHz	CDMA	5	wb	LTE (107)	18^h^ 20’/7 (10’ ON / 10’ OFF)	Multiple	Malignant and benign tumors	Incidence/ survival	
	32	NTP 2018^ (6)	2	Mice	B6C3F1/N	WT	M+F	N	180	1900 MHz	CDMA	10	wb	LTE (107)	18^h^ 20’/7 (10’ ON / 10’ OFF)	Multiple	Malignant and benign tumors	Incidence/ survival	
16 [81]	33	Oberto 2007 (1)	1	Mice	Eu- Pim1	Prone	M+F	N	100	900 MHz	GSM	0.5	wb	LTE (78)	1 h/d 7d/w	Multiple	Malignant tumors	Incidence/ survival	The study is a replica of Repacholi1997, but the exposure system is different shared sham
	34	Oberto 2007 (2)	1	Mice	Eu- Pim1	Prone	M+F	N	100	900 MHz	GSM	1.4	wb	LTE (78)	1 h/d 7d/w	Multiple	Malignant tumors	Incidence/ survival	
	35	Oberto 2007 (3)	1	Mice	Eu- Pim1	Prone	M+F	N	100	900 MHz	GSM	4	wb	LTE (78)	1 h/d 7d/w	Multiple	Malignant tumors	Incidence/ survival	
17 [70]	36	Rapacholi 1997	2	Mice	Eu- Pim1	Prone	F	N	100	900 MHz	GSM	0.13–1.4	wb	LTE (78)	2 x 30 min 7d/w	Lymphoma	Malignant tumors	Incidence	
18 [77]	37	Saran 2007 (1)	1	Mice	Patch ++	WT	M+F	N	50	900 MHz	GSM	0.4	wb	STE (>1=5d)	30 min x2/d 5d/w	CNS	Malignant tumors	Incidence/ survival	
	38	Saran 2007 (2)	1	Mice	Patch +-	Prone	M+F	N	50	900 MHz	GSM	0.4	wb	STE (>1=5d)	30 min x2/d 5d/w	CNS	Malignant tumors	Incidence/ survival	
19 [83]	39	Smith 2007 (1)	1	Rats	Wistar	WT	M+F	N	100	902 MHz	GSM	0.27	wb	LTE (104)	2 h/d 5d/w	Multiple	Malignant/ benign tumors	incidence	There are 2 sham groups for 6 exposed ones.
	40	Smith 2007 (2)	1	Rats	Wistar	WT	M+F	N	100	902 MHz	GSM	0.8	wb	LTE (104)	2 h/d 5d/w	Multiple	Malignant/ benign tumors	incidence	
	41	Smith 2007 (3)	1	Rats	Wistar	WT	M+F	N	100	902 MHz	GSM	2.42	wb	LTE (104)	2 h/d 5d/w	Multiple	Malignant/ benign tumors	incidence	
	42	Smith 2007 (4)	1	Rats	Wistar	WT	M+F	N	100	1747 MHz	DCS	0.29	wb	LTE (104)	2 h/d 5d/w	Multiple	Malignant/ benign tumors	incidence	
	43	Smith 2007 (5)	1	Rats	Wistar	WT	M+F	N	100	1747 MHz	DCS	0.87	wb	LTE (104)	2 h/d 5d/w	Multiple	Malignant/ benign tumors	incidence	
	44	Smith 2007 (6)	1	Rats	Wistar	WT	M+F	N	100	1747 MHz	DCS	2.61	wb	LTE (104)	2 h/d 5d/w	Multiple	Malignant/ benign tumors	incidence	
20 [72]	45	Sommer 2004	1	Mice	AKR/J mice	with retrovirus AKV	F	N	80	900 MHz	GSM	0.4	wb	MTE (42)	24 h/d 7d/w	Multiple	Malignant tumors	Incidence/ survival	
21 [71]	46	Sommer 2007	1	Mice	AKR/J mice	with retrovirus AKV	F	N	80	1966 MHz	UMTS	0.4	wb	MTE (42)	24 h/d 7d/w	Multiple	Malignant tumors	Incidence/ survival	
22 [78]	47	Szmigielski 1982 (1)	3	Mice	C3H/HeJ	Prone	F	N	40	2450 MHz	CW	2–3	wb	MTE (46)	2 h/d 6d/w	Breast cancer	Undefined	Incidence	It doesn’t discriminate between malignant and benign because tumors are detected by palpation only
	48	Szmigielski 1982 (2)	3	Mice	C3H/HeJ	Prone	F	N	40	2450 MHz	CW	6–8	wb	MTE (46)	2 h/d 6d/w	Breast cancer	Undefined	Incidence	
23 [84]	49	Tillmann 2007 (1)	1	Mice	B6C3F1	WT	M+F	N	100	902 MHz	GSM	0.29	wb	LTE (104)	2 h/d 5d/w	Multiple	Malignant/ benign tumors	Incidence	there are 2 sham for 6 exposed groups.
	50	Tillmann 2007 (2)	1	Mice	B6C3F1	WT	M+F	N	100	902 MHz	GSM	0.86	wb	LTE (104)	2 h/d 5d/w	Multiple	Malignant/ benign tumors	Incidence	
	51	Tillmann 2007 (3)	1	Mice	B6C3F1	WT	M+F	N	100	902 MHz	GSM	2.6	wb	LTE (104)	2 h/d 5d/w	Multiple	Malignant/ benign tumors	Incidence	
	52	Tillmann 2007 (4)	1	Mice	B6C3F1	WT	M+F	N	100	1747 MHz	DCS	0.29	wb	LTE (104)	2 h/d 5d/w	Multiple	Malignant/ benign tumors	Incidence	
	53	Tillmann 2007 (5)	1	Mice	B6C3F1	WT	M+F	N	100	1747 MHz	DCS	0.86	wb	LTE (104)	2 h/d 5d/w	Multiple	Malignant/ benign tumors	Incidence	
	54	Tillmann 2007 (6)	1	Mice	B6C3F1	WT	M+F	N	100	1747 MHz	DCS	2.6	wb	LTE (104)	2 h/d 5d/w	Multiple	Malignant/ benign tumors	Incidence	
24 [73]	55	Tillmann 2010 (1)	1	Mice	B6C3F1	WT	F	N	60	1966 MHz	UMTS	1.5-5	wb	LTE (104)	20 h/d 7d/w	Multiple	Malignant/ benign tumors	Incidence	SAR values related to growth
25 [74]	56	Toler 1997	1	Mice	C3H/HeJ	Prone	F	N	200	435 MHz	pulse: 1 μs; PRF: 1KHz	0.32	wb	LTE (91)	22 h/d 7d/w	Multiple	Malignant/ benign tumors	Incidence/ survival	
26 [85]	57	Utteridge 2002 (1)	1	Mice	E*μ*- Pim 1 ++	WT	F	N	120	898.4 MHz	GSM	0.25	wb	LTE (104)	1 h/d 5d/w	Multiple	Malignant tumors	Incidence/ survival	the study is a replica of Repacholi1997, but the exposure system is different Lymphoma and CNS only there are 2 sham for 8 exposed groups
	58	Utteridge 2002 (2)	1	Mice	E*μ*- Pim 1 ++	WT	F	N	120	898.4 MHz	GSM	1	wb	LTE (104)	1 h/d 5d/w	Multiple	Malignant tumors	Incidence/ survival	
	59	Utteridge 2002 (3)	1	Mice	E*μ*- Pim 1 ++	WT	F	N	120	898.4 MHz	GSM	2	wb	LTE (104)	1 h/d 5d/w	Multiple	Malignant tumors	Incidence/ survival	
	60	Utteridge 2002 (4)	1	Mice	E*μ*- Pim 1 ++	WT	F	N	120	898.4 MHz	GSM	4	wb	LTE (104)	1 h/d 5d/w	Multiple	Malignant tumors	Incidence/ survival	
	61	Utteridge 2002 (5)	1	Mice	E*μ*- Pim 1 + -	Prone	F	N	120	898.4 MHz	GSM	0.25	wb	LTE (104)	1 h/d 5d/w	Multiple	Malignant tumors	Incidence/ survival	
	62	Utteridge 2002 (6)	1	Mice	E*μ*- Pim 1 + -	Prone	F	N	120	898.4 MHz	GSM	1	wb	LTE (104)	1 h/d 5d/w	Multiple	Malignant tumors	Incidence/ survival	
	63	Utteridge 2002 (7)	1	Mice	E*μ*- Pim 1 + -	Prone	F	N	120	898.4 MHz	GSM	2	wb	LTE (104)	1 h/d 5d/w	Multiple	Malignant tumors	Incidence/ survival	
	64	Utteridge 2002 (8)	1	Mice	E*μ*- Pim 1 + -	Prone	F	N	120	898.4 MHz	GSM	4	wb	LTE (104)	1 h/d 5d/w	Multiple	Malignant tumors	Incidence/ survival	
27 [79]	65	Zook 2001 (1)	2	Rats	SD	WT	M+F	Y	60	860 MHz	digital modulation	1	local	LTE (95)	6 h/d 5d/w	CNS	Malignant tumors	Incidence/ survival	shared sham
	66	Zook 2001 (2)	2	Rats	SD	WT	M+F	Y	60	860 MHz	CW	1	local	LTE (95)	6 h/d 5d/w	CNS	Malignant tumors	Incidence/ survival	

(*) NTP 2018 incates the NTP experimental study on rats, while NTP 2018^ incates the NTP experimental study on mice.
