# Supplementary material for: In Vivo Studies on Radiofrequency (100 kHz–300 GHz) Electromagnetic Field Exposure and Cancer: A Systematic Review
Source: Int J Environ Res Public Health. 2023 Jan 23;20(3):2071. doi: 10.3390/ijerph20032071 (PMC9915925; doi:10.3390/ijerph20032071)
Supplement: Supplementary file 1 [file ijerph-20-02071-s001.zip › File S1.pdf]

Supplementary Material 1

Raw data in terms of malignant and benign tumor incidence for each considered organ/tumor

Table S1.1. Adrenals Malignant

Paper (treated/sham comparison)	SAR	Duration	Incidence exposed	n° exposed animals	Incidence sham	n° sham animals	Species	Genetic Background
Chou 1992 [63]	0.15-0.4	LTE	3	100	1	100	Rats	WT
Frei et al 1998 b [66]	1	LTE	1	98	2	99	Mice	Prone
Frei et al 1998 a [65]	0.3	LTE	0	95	1	93	Mice	Prone
La Regina 2003 (1) [76]	1.3 ± 0.5	LTE	1	160	3	160	Rats	WT
La Regina 2003 (2) [76]	1.3 ± 0.5	LTE	1	160	3	160	Rats	WT
NTP 2018 (1) [31]	1.5	LTE	4	180	3	180	Rats	WT
NTP 2018 (2) [31]	3	LTE	5	179	3	180	Rats	WT
NTP 2018 (3) [31]	6	LTE	1	178	3	180	Rats	WT
NTP 2018 (4) [31]	1.5	LTE	8	180	3	180	Rats	WT
NTP 2018 (5) [31]	3	LTE	6	180	3	180	Rats	WT
NTP 2018 (6) [31]	6	LTE	3	180	3	180	Rats	WT
NTP 2018 (1) [32]	2.5	LTE	0	177	2	174	Mice	WT
NTP 2018 (2) [32]	5	LTE	0	179	2	174	Mice	WT
NTP 2018 (3) [32]	10	LTE	0	178	2	174	Mice	WT
NTP 2018 (4) [32]	2.5	LTE	0	177	2	174	Mice	WT
NTP 2018 (5) [32]	5	LTE	1	177	2	174	Mice	WT
NTP 2018 (6) [32]	10	LTE	0	177	2	174	Mice	WT
Tillmann 2007 (1) [84]	0.29	LTE	0	100	0	100	Mice	WT
Tillmann 2007 (2) [84]	0.86	LTE	0	100	0	100	Mice	WT
Tillmann 2007 (3) [84]	2.6	LTE	1	100	0	100	Mice	WT
Tillmann 2007 (4) [84]	0.29	LTE	0	100	0	100	Mice	WT
Tillmann 2007 (5) [84]	0.86	LTE	1	100	0	100	Mice	WT
Tillmann 2007 (6) [84]	2.6	LTE	0	100	0	100	Mice	WT
Toler 1997 [74]	0.32	LTE	3	183	1	175	Mice	Prone
**Excluded papers**								
De Seze 2020 [64]	0.83	STE	1	12	0	24	Rats	WT
Jauchem 2001 [67]	0.01	MTE	4	98	2	97	Mice	Prone

Table S1.2. Bladder Malignant

Paper (treated/sham comparison)	SAR	Duration	Incidence exposed	n° exposed animals	Incidence sham	n° sham animals	Species	Genetic Background
Chou 1992[63]	0.15-0.4	LTE	1	100	0	100	Rats	WT
La Regina 2003 (1) [76]	1.3 ± 0.5	LTE	0	160	0	160	Rats	WT
La Regina 2003 (2) [76]	1.3 ± 0.5	LTE	1	160	0	160	Rats	WT
NTP 2018 (1) [31]	1.5	LTE	1	177	1	177	Rats	WT
NTP 2018 (2) [31]	3	LTE	0	176	1	177	Rats	WT
NTP 2018 (3) [31]	6	LTE	0	172	1	177	Rats	WT
NTP 2018 (4) [31]	1.5	LTE	0	171	1	177	Rats	WT
NTP 2018 (5) [31]	3	LTE	0	173	1	177	Rats	WT
NTP 2018 (6) [31]	6	LTE	0	168	1	177	Rats	WT
NTP 2018 (1) [32]	2.5	LTE	2	175	0	173	Mice	WT
NTP 2018 (2) [32]	5	LTE	0	176	0	173	Mice	WT
NTP 2018 (3) [32]	10	LTE	0	175	0	173	Mice	WT
NTP 2018 (4) [32]	2.5	LTE	0	171	0	173	Mice	WT
NTP 2018 (5) [32]	5	LTE	0	173	0	173	Mice	WT
NTP 2018 (6) [32]	10	LTE	0	168	0	173	Mice	WT

Table S1.3. Hystiocytic Sarcoma

Paper (treated/sham comparison)	SAR	Duration	Incidence exposed	n° exposed animals	Incidence sham	n° sham animals	Species	Genetic Background
Chou 1992 [63]	0.15-0.4	LTE	1	100	0	100	Rats	WT
La Regina 2003 (1) [76]	1.3 ± 0.5	LTE	1	160	0	160	Rats	WT
La Regina 2003 (2) [76]	1.3 ± 0.5	LTE	0	160	0	160	Rats	WT
NTP 2018 (1) [31]	1.5	LTE	1	180	0	180	Rats	WT
NTP 2018 (2) [31]	3	LTE	0	180	0	180	Rats	WT
NTP 2018 (3) [31]	6	LTE	1	180	0	180	Rats	WT
NTP 2018 (4) [31]	1.5	LTE	0	180	0	180	Rats	WT
NTP 2018 (5) [31]	3	LTE	2	180	0	180	Rats	WT
NTP 2018 (6) [31]	6	LTE	1	180	0	180	Rats	WT
NTP 2018 (1) [32]	2.5	LTE	2	179	8	180	Mice	WT
NTP 2018 (2) [32]	5	LTE	9	180	8	180	Mice	WT
NTP 2018 (3) [32]	10	LTE	7	180	8	180	Mice	WT
NTP 2018 (4) [32]	2.5	LTE	5	180	8	180	Mice	WT
NTP 2018 (5) [32]	5	LTE	3	180	8	180	Mice	WT
NTP 2018 (6) [32]	10	LTE	9	180	8	180	Mice	WT
Oberto 2007 (1) [81]	0.5	LTE	11	100	7	100	Mice	Prone
Oberto 2007 (2) [81]	1.4	LTE	5	100	7	100	Mice	Prone
Oberto 2007 (3) [81]	4	LTE	5	100	7	100	Mice	Prone
Tillmann 2007 (1) [84]	0.29	LTE	3	100	1	100	Mice	WT
Tillmann 2007 (2) [84]	0.86	LTE	2	100	1	100	Mice	WT
Tillmann 2007 (3) [84]	2.6	LTE	3	100	1	100	Mice	WT
Tillmann 2007 (4) [84]	0.29	LTE	5	100	1	100	Mice	WT
Tillmann 2007 (5) [84]	0.86	LTE	0	100	1	100	Mice	WT
Tillmann 2007 (6) [84]	2.6	LTE	2	100	1	100	Mice	WT
Tillmann 2010 [73]	1.5-5	LTE	0	56	3	54	Mice	WT
Toler 1997 [74]	0.32	LTE	1	189	3	181	Mice	Prone
**Excluded papers**								
De Seze 2020 [64]	0.83	STE	1	6	0	24	Rats	WT
Jauchem 2001 [67]	0.01	MTE	5	100	0	100	Mice	Prone

Table S1.4. Bone Marrow

Paper (treated/sham comparison)	SAR	Duration	Incidence exposed	n° exposed animals	Incidence sham	n° sham animals	Species	Genetic Background
Frei et al 1998 a [65]	0.3	LTE	0	100	1	99	Mice	Prone
NTP 2018 (1) [32]	2.5	LTE	1	178	2	180	Mice	WT
NTP 2018 (2) [32]	5	LTE	0	179	2	180	Mice	WT
NTP 2018 (3) [32]	10	LTE	0	180	2	180	Mice	WT
NTP 2018 (4) [32]	2.5	LTE	2	179	2	180	Mice	WT
NTP 2018 (5) [32]	5	LTE	1	180	2	180	Mice	WT
NTP 2018 (6) [32]	10	LTE	2	179	2	180	Mice	WT
**Excluded papers**								
De Seze 2020 [64]	0.83	STE	1	24	0	24	Rats	WT

Table S1.5. CNS Malignant

Paper (treated/sham comparison)	SAR	Duration	Incidence exposed	n° exposed animals	Incidence sham	n° sham animals	Species	Genetic Background
Adey 1999 [62]	1 - 1.60	LTE	2	60	7	60	Rats	WT
Adey 2000 [61]	0.74 - 1.60	LTE	4	90	1	90	Rats	WT
Anderson 2004 (1) [75]	0.16	LTE	3	180	4	180	Rats	WT
Anderson 2004 (2) [75]	1.6	LTE	6	180	4	180	Rats	WT
Falcioni 2018 (1) [80]	0.001	LTE	11	811	4	817	Rats	WT
Falcioni 2018 (2) [80]	0.03	LTE	5	411	4	817	Rats	WT
Falcioni 2018 (3) [80]	0.1	LTE	3	409	4	817	Rats	WT
La Regina 2003 (1) [76]	1.3 ± 0.5	LTE	2	160	2	160	Rats	WT
La Regina 2003 (2) [76]	1.3 ± 0.5	LTE	2	160	2	160	Rats	WT
NTP 2018 (1) [31]	1.5	LTE	4	180	0	180	Rats	WT
NTP 2018 (2) [31]	3	LTE	5	180	0	180	Rats	WT
NTP 2018 (3) [31]	6	LTE	4	180	0	180	Rats	WT
NTP 2018 (4) [31]	1.5	LTE	7	180	0	180	Rats	WT
NTP 2018 (5) [31]	3	LTE	1	180	0	180	Rats	WT
NTP 2018 (6) [31]	6	LTE	4	180	0	180	Rats	WT
Sommer 2007 [71]	0.4	MTE	6	160	4	160	Mice	Prone
Utteridge 2002 (1) [85]	0.25	LTE	17	120	11	120	Mice	WT
Utteridge 2002 (2) [85]	1	LTE	15	120	11	120	Mice	WT
Utteridge 2002 (3) [85]	2	LTE	10	120	11	120	Mice	WT
Utteridge 2002 (4) [85]	4	LTE	9	118	11	120	Mice	WT
Utteridge 2002 (5) [85]	0.25	LTE	4	120	1	120	Mice	Prone
Utteridge 2002 (6) [85]	1	LTE	0	120	1	120	Mice	Prone
Utteridge 2002 (7) [85]	2	LTE	2	120	1	120	Mice	Prone
Utteridge 2002 (8) [85]	4	LTE	2	120	1	120	Mice	Prone
Zook 2001 (1) [79]	1	LTE	5	60	3	60	Rats	WT
Zook 2001 (2) [79]	1	LTE	4	60	5	60	Rats	WT
**Excluded papers**								
Saran 2007 (2) [77]	0.4	STE	4	53	3	39	Mice	WT/Prone

Table S1.6. Brain Malignant

Paper (treated/sham comparison)	SAR	Duration	Incidence exposed	n° exposed animals	Incidence sham	n° sham animals	Species	Genetic Background
Adey 1999 [62]	1 - 1.60	LTE	2	60	5	60	Rats	WT
Adey 2000 [61]	0.74 - 1.60	LTE	3	90	1	90	Rats	WT
Anderson 2004 (1) [75]	0.16	LTE	3	180	4	180	Rats	WT
Anderson 2004 (2) [75]	1.6	LTE	6	180	4	180	Rats	WT
Falcioni 2018 (1) [80]	0.001	LTE	11	811	4	817	Rats	WT
Falcioni 2018 (2) [80]	0.03	LTE	5	411	4	817	Rats	WT
Falcioni 2018 (3) [80]	0.1	LTE	3	409	4	817	Rats	WT
La Regina 2003 (1) [76]	1.3 ± 0.5	LTE	2	160	2	160	Rats	WT
La Regina 2003 (2) [76]	1.3 ± 0.5	LTE	1	160	2	160	Rats	WT
NTP 2018 (1) [31]	1.5	LTE	3	180	0	180	Rats	WT
NTP 2018 (2) [31]	3	LTE	4	180	0	180	Rats	WT
NTP 2018 (3) [31]	6	LTE	4	180	0	180	Rats	WT
NTP 2018 (4) [31]	1.5	LTE	7	180	0	180	Rats	WT
NTP 2018 (5) [31]	3	LTE	1	180	0	180	Rats	WT
NTP 2018 (6) [31]	6	LTE	4	180	0	180	Rats	WT
Sommer 2007 [77]	0.4	MTE	6	160	4	160	Mice	Prone
Utteridge 2002 (1) [85]	0.25	LTE	17	120	11	120	Mice	WT
Utteridge 2002 (2) [85]	1	LTE	15	120	11	120	Mice	WT
Utteridge 2002 (3) [85]	2	LTE	10	120	11	120	Mice	WT
Utteridge 2002 (4) [85]	4	LTE	9	118	11	120	Mice	WT
Utteridge 2002 (5) [85]	0.25	LTE	4	120	1	120	Mice	Prone
Utteridge 2002 (6) [85]	1	LTE	0	120	1	120	Mice	Prone
Utteridge 2002 (7) [85]	2	LTE	2	120	1	120	Mice	Prone
Utteridge 2002 (8) [85]	4	LTE	2	120	1	120	Mice	Prone
Zook 2001 (1) [79]	1	LTE	5	60	3	60	Rats	WT
Zook 2001 (2) [79]	1	LTE	3	60	5	60	Rats	WT
**Excluded papers**								
Saran 2007 (2) [77]	0.4	STE	4	53	3	39	Mice	WT/Prone

Table S1.7.Sensorial System Malignant

Paper (treated/sham comparison)	SAR	Duration	Incidence exposed	n° exposed animals	Incidence sham	n° sham animals	Species	Genetic Background
La Regina 2003 (1) [76]	1.3 ± 0.5	LTE	2	160	0	160	Rats	WT
La Regina 2003 (2) [76]	1.3 ± 0.5	LTE	0	160	0	160	Rats	WT
NTP 2018 (1) [31]	1.5	LTE	0	180	0	173	Rats	WT
NTP 2018 (2) [31]	3	LTE	3	180	0	173	Rats	WT
NTP 2018 (3) [31]	6	LTE	2	180	0	173	Rats	WT
NTP 2018 (4) [31]	1.5	LTE	1	169	0	173	Rats	WT
NTP 2018 (5) [31]	3	LTE	0	169	0	173	Rats	WT
NTP 2018 (6) [31]	6	LTE	0	158	0	173	Rats	WT
NTP 2018 (1) [32]	2.5	LTE	3	179	3	179	Mice	WT
NTP 2018 (2) [32]	5	LTE	2	180	3	179	Mice	WT
NTP 2018 (3) [32]	10	LTE	0	180	3	179	Mice	WT
NTP 2018 (4) [32]	2.5	LTE	4	179	3	179	Mice	WT
NTP 2018 (5) [32]	5	LTE	2	179	3	179	Mice	WT
NTP 2018 (6) [32]	10	LTE	4	179	3	179	Mice	WT
Tillmann 2007 (1) [84]	0.29	LTE	1	100	0	100	Mice	WT
Tillmann 2007 (2) [84]	0.86	LTE	0	100	0	100	Mice	WT
Tillmann 2007 (3) [84]	2.6	LTE	0	100	0	100	Mice	WT
Tillmann 2007 (4) [84]	0.29	LTE	1	100	2	100	Mice	WT
Tillmann 2007 (5) [84]	0.86	LTE	0	101	2	100	Mice	WT
Tillmann 2007 (6) [84]	2.6	LTE	0	101	2	100	Mice	WT

Table S1.8. Uro-Genital male Malignant

Paper (treated/sham comparison)	SAR	Duration	Incidence exposed	n° exposed animals	Incidence sham	n° sham animals	Species	Genetic Background
Anderson 2004 (1) [75]	0.16	LTE	1	90	0	90	Rats	WT
Anderson 2004 (2) [75]	1.6	LTE	6	90	0	90	Rats	WT
La Regina 2003 (1) [76]	1.3 ± 0.5	LTE	5	80	3	80	Rats	WT
La Regina 2003 (2) [76]	1.3 ± 0.5	LTE	3	80	3	80	Rats	WT
NTP 2018 (1) M [31]	1.5	LTE	0	90	0	90	Rats	WT
NTP 2018 (2) M [31]	3	LTE	1	90	0	90	Rats	WT
NTP 2018 (3) M [31]	6	LTE	1	90	0	90	Rats	WT
NTP 2018 (4) M [31]	1.5	LTE	0	90	0	90	Rats	WT
NTP 2018 (5) M [31]	3	LTE	0	90	0	90	Rats	WT
NTP 2018 (6) M [31]	6	LTE	0	90	0	90	Rats	WT

1.9 Uro-Genital female Malignant

Paper (treated/sham comparison)	SAR	Duration	Incidence exposed	n° exposed animals	Incidence sham	n° sham animals	Species	Genetic Background
Frei et al 1998 b [65]	1	LTE	6	100	5	99	Mice	Prone
Frei et al 1998 a [66]	0.3	LTE	2	98	3	92	Mice	Prone
La Regina 2003 (1) [76]	1.3 ± 0.4	LTE	1	80	0	80	Rats	WT
La Regina 2003 (2) [76]	1.3 ± 0.5	LTE	0	80	0	80	Rats	WT
NTP 2018 (1) F [31]	1.5	LTE	4	90	11	90	Rats	WT
NTP 2018 (2) F [31]	3	LTE	10	90	11	90	Rats	WT
NTP 2018 (3) F [31]	6	LTE	9	90	11	90	Rats	WT
NTP 2018 (4) F [31]	1.5	LTE	2	90	11	90	Rats	WT
NTP 2018 (5)F [31]	3	LTE	6	90	11	90	Rats	WT
NTP 2018 (6) F [31]	6	LTE	5	90	11	90	Rats	WT
NTP 2018 (1) F [32]	2.5	LTE	3	90	3	89	Mice	WT
NTP 2018 (2) F [32]	5	LTE	1	90	3	89	Mice	WT
NTP 2018 (3) F [32]	10	LTE	1	89	3	89	Mice	WT
NTP 2018 (4) F [32]	2.5	LTE	5	84	3	89	Mice	WT
NTP 2018 (5) F [32]	5	LTE	1	84	3	89	Mice	WT
NTP 2018 (6) F [32]	10	LTE	5	83	3	89	Mice	WT
Oberto 2007 (1) [81]	0.5	LTE	2	50	1	50	Mice	Prone
Oberto 2007 (2) [81]	1.4	LTE	1	50	1	50	Mice	Prone
Oberto 2007 (3) [81]	4	LTE	2	50	1	50	Mice	Prone
Smith 2007 (1) [83]	0.41	LTE	1	50	0	50	Rats	WT
Smith 2007 (2) [83]	1.23	LTE	1	49	0	50	Rats	WT
Smith 2007 (3) [83]	3.7	LTE	1	50	0	50	Rats	WT
Smith 2007 (4) [83]	0.41	LTE	0	50	0	50	Rats	WT
Smith 2007 (5) [83]	1.33	LTE	1	50	0	50	Rats	WT
Smith 2007 (6) [83]	4	LTE	2	50	0	50	Rats	WT
Tillmann 2007 (1) [84]	0.29	LTE	2	50	2	50	Mice	WT
Tillmann 2007 (2) [84]	0.86	LTE	6	50	2	50	Mice	WT
Tillmann 2007 (3) [84]	2.6	LTE	2	50	2	50	Mice	WT
Tillmann 2007 (4) [84]	0.29	LTE	0	50	2	50	Mice	WT
Tillmann 2007 (5) [84]	0.86	LTE	1	50	2	50	Mice	WT
Tillmann 2007 (6) [84]	2.6	LTE	0	50	2	50	Mice	WT
Toler 1997 [74]	0.32	LTE	53	187	49	172	Mice	Prone

Table S1.10. Heart Malignant

Paper (treated/sham comparison)	SAR	Duration	Incidence exposed	n° exposed animals	Incidence sham	n° sham animals	Species	Genetic Background
Falcioni 2018 (1) [80]	0.001	LTE	12	811	4	817	Rats	WT
Falcioni 2018 (2) [80]	0.03	LTE	2	411	4	817	Rats	WT
Falcioni 2018 (3) [80]	0.1	LTE	5	409	4	817	Rats	WT
NTP 2018 (1) [31]	1.5	LTE	4	180	0	180	Rats	WT
NTP 2018 (2) [31]	3	LTE	4	180	0	180	Rats	WT
NTP 2018 (3) [31]	6	LTE	5	180	0	180	Rats	WT
NTP 2018 (4) [31]	1.5	LTE	4	180	0	180	Rats	WT
NTP 2018 (5) [31]	3	LTE	3	180	0	180	Rats	WT
NTP 2018 (6) [31]	6	LTE	8	180	0	180	Rats	WT
NTP 2018 (1) [32]	2.5	LTE	1	179	0	180	Mice	WT
NTP 2018 (2) [32]	5	LTE	1	180	0	180	Mice	WT
NTP 2018 (3) [32]	10	LTE	1	180	0	180	Mice	WT
NTP 2018 (4) [32]	2.5	LTE	1	180	0	180	Mice	WT
NTP 2018 (5) [32]	5	LTE	0	180	0	180	Mice	WT
NTP 2018 (6) [32]	10	LTE	0	180	0	180	Mice	WT

Table S1.11. Intestine Malignant

Paper (treated/sham comparison)	SAR	Duration	Incidence exposed	n° exposed animals	Incidence sham	n° sham animals	Species	Genetic Background
La Regina 2003 (1) [76]	1.3 ± 0.5	LTE	1	160	1	160	Rats	WT
La Regina 2003 (2) [76]	1.3 ± 0.5	LTE	0	160	1	160	Rats	WT
NTP 2018 (1) [31]	1.5	LTE	5	171	3	173	Rats	WT
NTP 2018 (2) [31]	3	LTE	1	174	3	173	Rats	WT
NTP 2018 (3) [31]	6	LTE	2	176	3	173	Rats	WT
NTP 2018 (4) [31]	1.5	LTE	1	173	3	173	Rats	WT
NTP 2018 (5) [31]	3	LTE	2	171	3	173	Rats	WT
NTP 2018 (6) [31]	6	LTE	0	164	3	173	Rats	WT
NTP 2018 (1) [32]	2.5	LTE	1	171	3	172	Mice	WT
NTP 2018 (2) [32]	5	LTE	0	174	3	172	Mice	WT
NTP 2018 (3) [32]	10	LTE	2	170	3	172	Mice	WT
NTP 2018 (4) [32]	2.5	LTE	1	174	3	172	Mice	WT
NTP 2018 (5) [32]	5	LTE	2	168	3	172	Mice	WT
NTP 2018 (6) [32]	10	LTE	2	170	3	172	Mice	WT
**Excluded papers**								
De Seze 2020 [64]	0.83	STE	1	12	1	23	Rats	WT

Table S1.12. Kidney Malignant

Paper (treated/sham comparison)	SAR	Duration	Incidence exposed	n° exposed animals	Incidence sham	n° sham animals	Species	Genetic Background
La Regina 2003 (1) [76]	1.3 ± 0.5	LTE	2	160	0	160	Rats	WT
La Regina 2003 (2) [76]	1.3 ± 0.6	LTE	0	160	0	160	Rats	WT
NTP 2018 (1) [31]	1.5	LTE	3	180	2	180	Rats	WT
NTP 2018 (2) [31]	3	LTE	1	180	2	180	Rats	WT
NTP 2018 (3) [31]	6	LTE	3	179	2	180	Rats	WT
NTP 2018 (4) [31]	1.5	LTE	0	180	2	180	Rats	WT
NTP 2018 (5) [31]	3	LTE	4	180	2	180	Rats	WT
NTP 2018 (6) [31]	6	LTE	3	176	2	180	Rats	WT
NTP 2018 (1) [32]	2.5	LTE	0	176	1	179	Mice	WT
NTP 2018 (2) [32]	5	LTE	0	179	1	179	Mice	WT
NTP 2018 (3) [32]	10	LTE	0	177	1	179	Mice	WT
NTP 2018 (4) [32]	2.5	LTE	0	178	1	179	Mice	WT
NTP 2018 (5) [32]	5	LTE	0	178	1	179	Mice	WT
NTP 2018 (6) [32]	10	LTE	0	177	1	179	Mice	WT
**Excluded papers**								
De Seze 2020 [64]	0.83	STE	1	24	0	24	Rats	WT

Table S1.13. Leukemia

Paper (treated/sham comparison)	SAR	Duration	Incidence exposed	n° exposed animals	Incidence sham	n° sham animals	Species	Genetic Background
Anderson 2004 (1) [75]	0.16	LTE	45	180	62	180	Rats	WT
Anderson 2004 (2) [75]	1.6	LTE	46	180	62	180	Rats	WT
Chou 1992 [63]	0.15-0.4	LTE	2	100	1	100	Rats	WT
La Regina 2003 (1) [76]	1.3 ± 0.5	LTE	62	160	57	160	Rats	WT
La Regina 2003 (2) [76]	1.3 ± 0.5	LTE	45	160	57	160	Rats	WT
NTP 2018 (1) [31]	1.5	LTE	1	180	0	180	Rats	WT
NTP 2018 (2) [31]	3	LTE	1	180	0	180	Rats	WT
NTP 2018 (3) [31]	6	LTE	1	180	0	180	Rats	WT
NTP 2018 (4) [31]	1.5	LTE	5	180	0	180	Rats	WT
NTP 2018 (5) [31]	3	LTE	4	180	0	180	Rats	WT
NTP 2018 (6) [31]	6	LTE	1	180	0	180	Rats	WT
NTP 2018 (1) [32]	2.5	LTE	1	179	0	180	Mice	WT
NTP 2018 (2) [32]	5	LTE	0	180	0	180	Mice	WT
NTP 2018 (3) [32]	10	LTE	1	180	0	180	Mice	WT
NTP 2018 (4) [32]	2.5	LTE	1	180	0	180	Mice	WT
NTP 2018 (5) [32]	5	LTE	2	180	0	180	Mice	WT
NTP 2018 (6) [32]	10	LTE	1	180	0	180	Mice	WT

Table S1.14. Liver Malignant

Paper (treated/sham comparison)	SAR	Duration	Incidence exposed	n° exposed animals	Incidence sham	n° sham animals	Species	Genetic Background
Chou 1992 [63]	0.15-0.4	LTE	0	100	1	100	Rats	WT
Frei et al 1998 a [65]	0.3	LTE	1	99	3	93	Mice	Prone
NTP 2018 (1) [31]	1.5	LTE	1	180	0	180	Rats	WT
NTP 2018 (2) [31]	3	LTE	1	180	0	180	Rats	WT
NTP 2018 (3) [31]	6	LTE	0	180	0	180	Rats	WT
NTP 2018 (4) [31]	1.5	LTE	0	179	0	180	Rats	WT
NTP 2018 (5) [31]	3	LTE	1	180	0	180	Rats	WT
NTP 2018 (6) [31]	6	LTE	2	180	0	180	Rats	WT
NTP 2018 (1) [32]	2.5	LTE	42	179	45	179	Mice	WT
NTP 2018 (2) [32]	5	LTE	49	180	45	179	Mice	WT
NTP 2018 (3) [32]	10	LTE	32	179	45	179	Mice	WT
NTP 2018 (4) [32]	2.5	LTE	36	177	45	179	Mice	WT
NTP 2018 (5) [32]	5	LTE	49	180	45	179	Mice	WT
NTP 2018 (6) [32]	10	LTE	46	180	45	179	Mice	WT
Oberto 2007 (1) [81]	0.5	LTE	0	100	1	100	Mice	Prone
Oberto 2007 (2) [81]	1.4	LTE	0	100	1	100	Mice	Prone
Oberto 2007 (3) [81]	4	LTE	5	100	1	100	Mice	Prone
Tillmann 2007 (1) [84]	0.29	LTE	8	100	10	100	Mice	WT
Tillmann 2007 (2) [84]	0.86	LTE	8	100	10	100	Mice	WT
Tillmann 2007 (3) [84]	2.6	LTE	11	100	10	100	Mice	WT
Tillmann 2007 (4) [84]	0.29	LTE	9	100	9	100	Mice	WT
Tillmann 2007 (5) [84]	0.86	LTE	5	100	9	100	Mice	WT
Tillmann 2007 (6) [84]	2.6	LTE	10	100	9	100	Mice	WT
Tillmann 2010 [73]	1.5-5	LTE	13	56	8	54	Mice	WT
Toler 1997 [74]	0.32	LTE	13	188	13	180	Mice	Prone
**Excluded papers**								
De Seze 2020 [64]	0.83	LTE	0	24	2	23	Rats	WT

Table S1.15. Lung Malignant

Paper (treated/sham comparison)	SAR	Duration	Incidence exposed	n° exposed animals	Incidence sham	n° sham animals	Species	Genetic Background
Frei et al 1998 a [65]	0.3	LTE	1	99	1	97	Mice	Prone
La Regina 2003 (1) [76]	1.3 ± 0.5	LTE	0	160	3	160	Rats	WT
La Regina 2003 (2) [76]	1.3 ± 0.5	LTE	2	160	3	160	Rats	WT
NTP 2018 (1) [31]	1.5	LTE	2	180	0	180	Rats	WT
NTP 2018 (2) [31]	3	LTE	0	180	0	180	Rats	WT
NTP 2018 (3) [31]	6	LTE	0	180	0	180	Rats	WT
NTP 2018 (4) [31]	1.5	LTE	0	180	0	180	Rats	WT
NTP 2018 (5) [31]	3	LTE	1	180	0	180	Rats	WT
NTP 2018 (6) [31]	6	LTE	0	180	0	180	Rats	WT
NTP 2018 (1) [32]	2.5	LTE	13	180	16	180	Mice	WT
NTP 2018 (2) [32]	5	LTE	16	180	16	180	Mice	WT
NTP 2018 (3) [32]	10	LTE	19	180	16	180	Mice	WT
NTP 2018 (4) [32]	2.5	LTE	16	179	16	180	Mice	WT
NTP 2018 (5) [32]	5	LTE	14	180	16	180	Mice	WT
NTP 2018 (6) [32]	10	LTE	16	180	16	180	Mice	WT
Sommer 2004 [72]	0.4	MTE	16	160	16	160	Mice	retrovirus
Tillmann 2007 (1) [84]	0.29	LTE	5	100	10	100	Mice	WT
Tillmann 2007 (2) [84]	0.86	LTE	9	100	10	100	Mice	WT
Tillmann 2007 (3) [84]	2.6	LTE	5	100	10	100	Mice	WT
Tillmann 2007 (4) [84]	0.29	LTE	5	100	7	100	Mice	WT
Tillmann 2007 (5) [84]	0.86	LTE	4	100	7	100	Mice	WT
Tillmann 2007 (6) [84]	2.6	LTE	4	100	7	100	Mice	WT
Tillmann 2010 [73]	1.5-5	LTE	1	56	0	54	Mice	WT

Table S1.16. Lymphoma

Paper (treated/sham comparison)	SAR	Duration	Incidence exposed	n° exposed animals	Incidence sham	n° sham animals	Species	Genetic Background
Anderson 2004 (1) [75]	0.16	LTE	0	180	1	180	Rats	WT
Anderson 2004 (2) [75]	1.6	LTE	0	180	1	180	Rats	WT
Chou 1992 [63]	0.15-0.4	LTE	2	100	2	100	Rats	WT
Frei et al 1998 a [65]	0.3	LTE	6	99	2	97	Mice	Prone
La Regina 2003 (1) [76]	1.3 ± 0.5	LTE	2	160	2	160	Rats	WT
La Regina 2003 (2) [76]	1.3 ± 0.5	LTE	0	160	2	160	Rats	WT
Lee 2011 [69]	6	MTE	62	80	62	80	Mice	Prone
NTP 2018 (1) [31]	1.5	LTE	8	180	7	180	Rats	WT
NTP 2018 (2) [31]	3	LTE	6	180	7	180	Rats	WT
NTP 2018 (3) [31]	6	LTE	7	180	7	180	Rats	WT
NTP 2018 (4) [31]	1.5	LTE	4	180	7	180	Rats	WT
NTP 2018 (5) [31]	3	LTE	5	180	7	180	Rats	WT
NTP 2018 (6) [31]	6	LTE	7	180	7	180	Rats	WT
NTP 2018 (1) [32]	2.5	LTE	17	179	8	180	Mice	WT
NTP 2018 (2) [32]	5	LTE	12	180	8	180	Mice	WT
NTP 2018 (3) [32]	10	LTE	10	180	8	180	Mice	WT
NTP 2018 (4) [32]	2.5	LTE	12	179	8	180	Mice	WT
NTP 2018 (5) [32]	5	LTE	11	180	8	180	Mice	WT
NTP 2018 (6) [32]	10	LTE	11	180	8	180	Mice	WT
Oberto 2007 (1) [81]	0.5	LTE	28	100	31	100	Mice	Prone
Oberto 2007 (2) [81]	1.4	LTE	40	100	31	100	Mice	Prone
Oberto 2007 (3)	4	LTE	23	100	31	100	Mice	Prone
Rapacholi 1997 [70]	0.13-1.4	LTE	43	94	22	93	Mice	Prone
Sommer 2004 [72]	0.4	MTE	144	160	144	160	Mice	Prone
Sommer 2007 [71]	0.4	MTE	141	160	149	160	Mice	Prone
Tillmann 2007 (1) [84]	0.29	LTE	22	100	23	100	Mice	WT
Tillmann 2007 (2) [84]	0.86	LTE	25	100	23	100	Mice	WT
Tillmann 2007 (3) [84]	2.6	LTE	24	100	23	100	Mice	WT
Tillmann 2007 (4) [84]	0.29	LTE	22	100	23	100	Mice	WT
Tillmann 2007 (5) [84]	0.86	LTE	22	100	23	100	Mice	WT
Tillmann 2007 (6) [84]	2.6	LTE	21	100	23	100	Mice	WT
Tillmann 2010 [73]	1.5-5	LTE	16	56	17	54	Mice	WT
Toler 1997 [74]	0.32	LTE	13	200	13	200	Mice	Prone
Utteridge 2002 (1) [85]	0.25	LTE	40	120	38	120	Mice	WT
Utteridge 2002 (2) [85]	1	LTE	37	120	38	120	Mice	WT
Utteridge 2002 (3) [85]	2	LTE	45	120	38	120	Mice	WT
Utteridge 2002 (4) [85]	4	LTE	36	118	38	120	Mice	WT
Utteridge 2002 (5) [85]	0.25	LTE	88	120	89	120	Mice	Prone
Utteridge 2002 (6) [85]	1	LTE	86	120	89	120	Mice	Prone
Utteridge 2002 (7) [85]	2	LTE	93	120	89	120	Mice	Prone
Utteridge 2002 (8) [85]	4	LTE	99	120	89	120	Mice	Prone

Table S1.17. Mammary malignant

Paper (treated/sham comparison)	SAR	Duration	Incidence exposed	n° exposed animals	Incidence sham	n° sham animals	Species	Genetic Background
Anderson 2004 (1) [75]	0.16	LTE	1	180	1	180	Rats	WT
Anderson 2004 (2) [75]	1.6	LTE	2	180	1	180	Rats	WT
Bartsch 2010 (3) [82]	0.038-0.08	LTE	9	29	12	28	Rats	WT
Bartsch 2010 (4) [82]	0.038-0.08	LTE	7	29	10	30	Rats	WT
Frei et al 1998 b [66]	1	LTE	38	98	30	99	Mice	Prone
Frei et al 1998 a [65]	0.3	LTE	44	95	55	88	Mice	Prone
La Regina 2003 (1) [76]	1.3 ± 0.5	LTE	1	160	2	160	Rats	WT
La Regina 2003 (2) [76]	1.3 ± 0.5	LTE	0	160	2	160	Rats	WT
NTP 2018 (1) [31]	1.5	LTE	6	165	10	172	Rats	WT
NTP 2018 (2) [31]	3	LTE	9	171	10	172	Rats	WT
NTP 2018 (3) [31]	6	LTE	6	172	10	172	Rats	WT
NTP 2018 (4) [31]	1.5	LTE	8	167	10	172	Rats	WT
NTP 2018 (5) [31]	3	LTE	6	170	10	172	Rats	WT
NTP 2018 (6) [31]	6	LTE	4	170	10	172	Rats	WT
NTP 2018 (1) [32]	2.5	LTE	0	93	0	87	Mice	WT
NTP 2018 (2) [32]	5	LTE	1	90	0	87	Mice	WT
NTP 2018 (3) [32]	10	LTE	1	92	0	87	Mice	WT
NTP 2018 (4) [32]	2.5	LTE	0	88	0	87	Mice	WT
NTP 2018 (5) [32]	5	LTE	0	90	0	87	Mice	WT
NTP 2018 (6) [32]	10	LTE	2	91	0	87	Mice	WT
Smith 2007 (1) [83]	0.27	LTE	4	100	2	100	Rats	WT
Smith 2007 (2) [83]	0.8	LTE	1	100	2	100	Rats	WT
Smith 2007 (3) [83]	2.42	LTE	2	100	2	100	Rats	WT
Smith 2007 (4) [83]	0.29	LTE	4	100	3	100	Rats	WT
Smith 2007 (5) [83]	0.87	LTE	2	100	3	100	Rats	WT
Smith 2007 (6) [83]	2.61	LTE	3	99	3	100	Rats	WT
Szmigielski 1982 (1) [78]	2-3	MTE	32	40	14	40	Mice	Prone
Szmigielski 1982 (2) [78]	6-8	MTE	37	40	14	40	Mice	Prone
Toler 1997	0.32	LTE	85	193	82	190	Mice	Prone
**Excluded Papers**								
Jauchem 2001 [67]	0.01	MTE	47	100	51	100	Mice	Prone
De Seze 2020 [64]	0.83	STE	1	24	0	24	Rats	WT

Table S1.18. Mesenteric Lymphonode malignant

Paper (treated/sham comparison)	SAR	Duration	Incidence exposed	n° exposed animals	Incidence sham	n° sham animals	Species	Genetic Background
Frei et al 1998 b [66]	1	LTE	3	95	1	98	Mice	Prone
NTP 2018 (1) [31]	1.5	LTE	0	180	0	180	Rats	WT
NTP 2018 (2) [31]	3	LTE	0	180	0	180	Rats	WT
NTP 2018 (3) [31]	6	LTE	2	180	0	180	Rats	WT
NTP 2018 (1) [32]	2.5	LTE	0	172	1	156	Mice	WT
NTP 2018 (2) [32]	5	LTE	0	166	1	156	Mice	WT
NTP 2018 (3) [32]	10	LTE	1	168	1	156	Mice	WT
NTP 2018 (4) [32]	2.5	LTE	1	174	1	156	Mice	WT
NTP 2018 (5) [32]	5	LTE	0	161	1	156	Mice	WT
NTP 2018 (6) [32]	10	LTE	0	166	1	156	Mice	WT
Smith 2007 (1) [83]	0.27	LTE	0	99	0	98	Rats	WT
Smith 2007 (2) [83]	0.8	LTE	1	100	0	98	Rats	WT
Smith 2007 (3) [83]	2.42	LTE	2	100	0	98	Rats	WT
Smith 2007 (4) [83]	0.29	LTE	0	100	2	99	Rats	WT
Smith 2007 (5) [83]	0.87	LTE	0	100	2	99	Rats	WT
Smith 2007 (6) [83]	2.61	LTE	0	100	2	99	Rats	WT

Table S1.19. Pancreas Malignant

Paper (treated/sham comparison)	SAR	Duration	Incidence exposed	n° exposed animals	Incidence sham	n° sham animals	Species	Genetic Background
NTP 2018 (1) [31]	1.5	LTE	17	178	11	180	Rats	WT
NTP 2018 (2) [31]	3	LTE	18	178	11	180	Rats	WT
NTP 2018 (3) [31]	6	LTE	9	173	11	180	Rats	WT
NTP 2018 (4) [31]	1.5	LTE	9	178	11	180	Rats	WT
NTP 2018 (5) [31]	3	LTE	15	177	11	180	Rats	WT
NTP 2018 (6) [31]	6	LTE	8	167	11	180	Rats	WT
NTP 2018 (1) [32]	2.5	LTE	1	176	1	175	Mice	WT
NTP 2018 (2) [32]	5	LTE	2	179	1	175	Mice	WT
NTP 2018 (3) [32]	10	LTE	2	175	1	175	Mice	WT
NTP 2018 (4) [32]	2.5	LTE	1	178	1	175	Mice	WT
NTP 2018 (5) [32]	5	LTE	0	178	1	175	Mice	WT
NTP 2018 (6) [32]	10	LTE	0	176	1	175	Mice	WT
Smith 2007 (1) [83]	0.27	LTE	0	100	1	100	Rats	WT
Smith 2007 (2) [83]	0.8	LTE	0	100	1	100	Rats	WT
Smith 2007 (3) [83]	2.42	LTE	2	100	1	100	Rats	WT
Smith 2007 (4) [83]	0.29	LTE	0	100	0	100	Rats	WT
Smith 2007 (5) [83]	0.87	LTE	2	100	0	100	Rats	WT
Smith 2007 (6) [83]	2.61	LTE	0	100	0	100	Rats	WT
**Excluded papers**								
De Seze 2020 [64]	0.83	STE	1	24	0	24	Rats	WT

Table S1.20. Pituitary Malignant

Paper (treated/sham comparison)	SAR	Duration	Incidence exposed	n° exposed animals	Incidence sham	n° sham animals	Species	Genetic Background
Bartsch 2010 (1) [82]	0.038-0.08	LTE	5	12	9	12	Rats	WT
Bartsch 2010 (2) [82]	0.038-0.08	LTE	4	12	6	12	Rats	WT
Bartsch 2010 (3) [82]	0.038-0.08	LTE	8	29	6	28	Rats	WT
Bartsch 2010 (4) [82]	0.038-0.08	LTE	11	29	11	30	Rats	WT
Chou 1992 [63]	0.15-0.4	LTE	2	100	0	100	Rats	WT
La Regina 2003 (1) [76]	1.3 ± 0.5	LTE	0	160	0	160	Rats	WT
La Regina 2003 (2) [76]	1.3 ± 0.4	LTE	1	160	0	160	Rats	WT
NTP 2018 (1) [31]	1.5	LTE	1	180	1	179	Rats	WT
NTP 2018 (2) [31]	3	LTE	0	180	1	179	Rats	WT
NTP 2018 (3) [31]	6	LTE	1	180	1	179	Rats	WT
NTP 2018 (4) [31]	1.5	LTE	1	179	1	179	Rats	WT
NTP 2018 (5) [31]	3	LTE	1	179	1	179	Rats	WT
NTP 2018 (6) [31]	6	LTE	0	180	1	179	Rats	WT
NTP 2018 (1) [32]	2.5	LTE	0	165	0	166	Mice	WT
NTP 2018 (2) [32]	5	LTE	2	171	0	166	Mice	WT
NTP 2018 (3) [32]	10	LTE	1	169	0	166	Mice	WT
NTP 2018 (4) [32]	2.5	LTE	0	163	0	166	Mice	WT
NTP 2018 (5) [32]	5	LTE	1	177	0	166	Mice	WT
NTP 2018 (6) [32]	10	LTE	1	169	0	166	Mice	WT
Smith 2007 (4) [83]	0.29	LTE	0	100	0	100	Rats	WT
Smith 2007 (5) [83]	0.87	LTE	1	100	0	100	Rats	WT
Smith 2007 (6) [83]	2.61	LTE	1	100	0	100	Rats	WT
Tillmann 2007 (1) [84]	0.29	LTE	0	100	0	100	Mice	WT
Tillmann 2007 (2) [84]	0.86	LTE	0	99	0	100	Mice	WT
Tillmann 2007 (3) [84]	2.6	LTE	2	100	0	100	Mice	WT
**Excluded papers**								
De Seze 2020 [64]	0.83	STE	1	24	0	24	Rats	WT

Table S1.21. Skin Malignant

Paper (treated/sham comparison)	SAR	Duration	Incidence exposed	n° exposed animals	Incidence sham	n° sham animals	Species	Genetic Background
Chou 1992 [63]	0.15-0.4	LTE	2	100	0	100	Rats	WT
Frei et al 1998 b [66]	1	LTE	4	100	7	100	Mice	Prone
Frei et al 1998 a [65]	0.3	LTE	3	98	4	98	Mice	Prone
NTP 2018 (1) [31]	1.5	LTE	6	180	6	180	Rats	WT
NTP 2018 (2) [31]	3	LTE	7	180	6	180	Rats	WT
NTP 2018 (3) [31]	6	LTE	5	180	6	180	Rats	WT
NTP 2018 (4) [31]	1.5	LTE	6	180	6	180	Rats	WT
NTP 2018 (5) [31]	3	LTE	6	180	6	180	Rats	WT
NTP 2018 (6) [31]	6	LTE	2	180	6	180	Rats	WT
NTP 2018 (1) [32]	2.5	LTE	3	178	10	180	Mice	WT
NTP 2018 (2) [32]	5	LTE	14	180	10	180	Mice	WT
NTP 2018 (3) [32]	10	LTE	6	180	10	180	Mice	WT
NTP 2018 (4) [32]	2.5	LTE	3	180	10	180	Mice	WT
NTP 2018 (5) [32]	5	LTE	7	180	10	180	Mice	WT
NTP 2018 (6) [32]	10	LTE	5	180	10	180	Mice	WT
**Excluded papers**								
De Seze 2020 [64]	0.83	STE	1	8	3	23	Rats	WT
Jauchem 2001 [67]	0.01	MTE	5	100	4	100	Mice	Prone

Table S1.22. Spleen Malignant

Paper (treated/sham comparison)	SAR	Duration	Incidence exposed	n° exposed animals	Incidence sham	n° sham animals	Species	Genetic Background
Frei et al 1998 b [66]	1	LTE	3	101	1	99	Mice	Prone
NTP 2018 (1) [31]	1.5	LTE	0	180	4	180	Rats	WT
NTP 2018 (2) [31]	3	LTE	0	179	4	180	Rats	WT
NTP 2018 (3) [31]	6	LTE	2	180	4	180	Rats	WT
NTP 2018 (4) [31]	1.5	LTE	0	180	4	180	Rats	WT
NTP 2018 (5) [31]	3	LTE	0	180	4	180	Rats	WT
NTP 2018 (6) [31]	6	LTE	0	175	4	180	Rats	WT
NTP 2018 (1) [32]	2.5	LTE	6	175	0	173	Mice	WT
NTP 2018 (2) [32]	5	LTE	3	178	0	173	Mice	WT
NTP 2018 (3) [32]	10	LTE	1	175	0	173	Mice	WT
NTP 2018 (4) [32]	2.5	LTE	5	176	0	173	Mice	WT
NTP 2018 (5) [32]	5	LTE	3	173	0	173	Mice	WT
NTP 2018 (6) [32]	10	LTE	3	174	0	173	Mice	WT
Tillmann 2010 [73]	1.5-5	LTE	1	56	1	54	Mice	WT

Table S1.23. Stomach Malignant

Paper (treated/sham comparison)	SAR	Duration	Incidence exposed	n° exposed animals	Incidence sham	n° sham animals	Species	Genetic Background
Chou 1992 [63]	0.15-0.4	LTE	1	100	0	100	Rats	WT
NTP 2018 (1) [31]	1.5	LTE	0	180	1	180	Rats	WT
NTP 2018 (2) [31]	3	LTE	0	180	1	180	Rats	WT
NTP 2018 (3) [31]	6	LTE	2	180	1	180	Rats	WT
NTP 2018 (4) [31]	1.5	LTE	0	180	1	180	Rats	WT
NTP 2018 (5) [31]	3	LTE	0	179	1	180	Rats	WT
NTP 2018 (6) [31]	6	LTE	1	180	1	180	Rats	WT

Table S1.24. Thymus Malignant

Paper (treated/sham comparison)	SAR	Duration	Incidence exposed	n° exposed animals	Incidence sham	n° sham animals	Species	Genetic Background
La Regina 2003 (1) [76]	1.3 ± 0.5	LTE	1	160	0	160	Rats	WT
La Regina 2003 (2) [76]	1.3 ± 0.6	LTE	0	160	0	160	Rats	WT
NTP 2018 (1) [31]	1.5	LTE	1	172	1	175	Rats	WT
NTP 2018 (2) [31]	3	LTE	1	176	1	175	Rats	WT
NTP 2018 (3) [31]	6	LTE	1	172	1	175	Rats	WT
NTP 2018 (4) [31]	1.5	LTE	0	168	1	175	Rats	WT
NTP 2018 (5) [31]	3	LTE	1	174	1	175	Rats	WT
NTP 2018 (6) [31]	6	LTE	0	169	1	175	Rats	WT
Smith 2007 (1) [83]	0.27	LTE	1	97	0	98	Rats	WT
Smith 2007 (2) [83]	0.8	LTE	1	99	0	98	Rats	WT
Smith 2007 (3) [83]	2.42	LTE	1	99	0	98	Rats	WT
Smith 2007 (4) [83]	0.29	LTE	1	100	2	94	Rats	WT
Smith 2007 (5) [83]	0.87	LTE	1	97	2	94	Rats	WT
Smith 2007 (6) [83]	2.61	LTE	1	95	2	94	Rats	WT

Table S1.25. Thyroid Malignant

Paper (treated/sham comparison)	SAR	Duration	Incidence exposed	n° exposed animals	Incidence sham	n° sham animals	Species	Genetic Background
Anderson 2004 (1) [75]	0.16	LTE	16	180	11	180	Rats	WT
Anderson 2004 (2) [75]	1.6	LTE	17	180	11	180	Rats	WT
Chou 1992 [63]	0.15-0.4	LTE	2	100	0	100	Rats	WT
La Regina 2003 (1) [76]	1.3 ± 0.5	LTE	2	160	5	160	Rats	WT
La Regina 2003 (2) [76]	1.3 ± 0.5	LTE	2	160	5	160	Rats	WT
NTP 2018 (1) [31]	1.5	LTE	2	177	3	179	Rats	WT
NTP 2018 (2) [31]	3	LTE	3	179	3	179	Rats	WT
NTP 2018 (3) [31]	6	LTE	5	175	3	179	Rats	WT
NTP 2018 (4) [31]	1.5	LTE	4	177	3	179	Rats	WT
NTP 2018 (5) [31]	3	LTE	5	176	3	179	Rats	WT
NTP 2018 (6) [31]	6	LTE	6	174	3	179	Rats	WT
NTP 2018 (1) [32]	2.5	LTE	0	177	1	175	Mice	WT
NTP 2018 (2) [32]	5	LTE	1	174	1	175	Mice	WT
NTP 2018 (3) [32]	10	LTE	0	174	1	175	Mice	WT
NTP 2018 (4) [32]	2.5	LTE	0	176	1	175	Mice	WT
NTP 2018 (5) [32]	5	LTE	0	176	1	175	Mice	WT
NTP 2018 (6) [32]	10	LTE	0	175	1	175	Mice	WT
Oberto 2007 (1) [81]	0.5	LTE	0	100	0	100	Mice	Prone
Oberto 2007 (2) [81]	1.4	LTE	1	100	0	100	Mice	Prone
Oberto 2007 (3) [81]	4	LTE	0	100	0	100	Mice	Prone
Smith 2007 (1) [83]	0.27	LTE	1	100	0	100	Rats	WT
Smith 2007 (2) [83]	0.8	LTE	1	100	0	100	Rats	WT
Smith 2007 (3) [83]	2.42	LTE	1	100	0	100	Rats	WT
Smith 2007 (4) [83]	0.29	LTE	0	100	1	100	Rats	WT
Smith 2007 (5) [83]	0.87	LTE	2	100	1	100	Rats	WT
Smith 2007 (6) [83]	2.61	LTE	1	100	1	100	Rats	WT

Table S1.26. Adrenals Benign

Paper (treated/sham comparison)	SAR	Duration	Incidence exposed	n° exposed animals	Incidence sham	n° sham animals	Species	Genetic Background
Chou 1992 [63]	0.15-0.4	LTE	17	100	12	100	Rats	WT
Frei et al 1998 b [66]	1	LTE	1	99	4	100	Mice	Prone
Frei et al 1998 a [65]	0.3	LTE	2	95	2	93	Mice	Prone
La Regina 2003 (1) [76]	1.3 ± 0.5	LTE	6	160	12	160	Rats	WT
La Regina 2003 (2) [76]	1.3 ± 0.5	LTE	12	160	12	160	Rats	WT
NTP 2018 (1) [31]	1.5	LTE	35	180	13	180	Rats	WT
NTP 2018 (2) [31]	3	LTE	31	179	13	180	Rats	WT
NTP 2018 (3) [31]	6	LTE	22	178	13	180	Rats	WT
NTP 2018 (4) [31]	1.5	LTE	31	180	13	180	Rats	WT
NTP 2018 (5) [31]	3	LTE	27	180	13	180	Rats	WT
NTP 2018 (6) [31]	6	LTE	18	180	13	180	Rats	WT
NTP 2018 (1) [32]	2.5	LTE	3	177	1	174	Mice	WT
NTP 2018 (2) [32]	5	LTE	4	179	1	174	Mice	WT
NTP 2018 (3) [32]	10	LTE	0	178	1	174	Mice	WT
NTP 2018 (4) [32]	2.5	LTE	2	177	1	174	Mice	WT
NTP 2018 (5) [32]	5	LTE	5	177	1	174	Mice	WT
NTP 2018 (6) [32]	10	LTE	6	177	1	174	Mice	WT
Oberto 2007 (1) [81]	0.5	LTE	0	100	2	100	Mice	Prone
Oberto 2007 (2) [81]	1.4	LTE	3	100	2	100	Mice	Prone
Oberto 2007 (3) [81]	4	LTE	1	100	2	100	Mice	Prone
Tillmann 2007 (1) [84]	0.29	LTE	4	100	7	100	Mice	WT
Tillmann 2007 (2) [84]	0.86	LTE	7	100	7	100	Mice	WT
Tillmann 2007 (3) [84]	2.6	LTE	2	100	7	100	Mice	WT
Tillmann 2007 (4)	0.29	LTE	5	100	3	100	Mice	WT
Tillmann 2007 (5) [84]	0.86	LTE	4	100	3	100	Mice	WT
Tillmann 2007 (6) [84]	2.6	LTE	1	100	3	100	Mice	WT

Table S1.27. Brain Benign

Paper (treated/sham comparison)	SAR	Duration	Incidence exposed	n° exposed animals	Incidence sham	n° sham animals	Species	Genetic Background
Falcioni 2018 (1) [80]	0.001	LTE	8	811	2	817	Rats	WT
Falcioni 2018 (2) [80]	0.03	LTE	3	411	2	817	Rats	WT
Falcioni 2018 (3) [80]	0.1	LTE	4	409	2	817	Rats	WT
NTP 2018 (1) [31]	1.5	LTE	4	180	2	180	Rats	WT
NTP 2018 (2) [31]	3	LTE	5	180	2	180	Rats	WT
NTP 2018 (3) [31]	6	LTE	3	180	2	180	Rats	WT
NTP 2018 (4) [31]	1.5	LTE	1	180	2	180	Rats	WT
NTP 2018 (5) [31]	3	LTE	2	180	2	180	Rats	WT
NTP 2018 (6) [31]	6	LTE	4	180	2	180	Rats	WT
**Excluded papers**								
De Seze 2020 [64]	0.83	STE	1	24	0	24	Rats	WT

Table S1.28. Sensorial System Benign

Paper (treated/sham comparison)	SAR	Duration	Incidence exposed	n° exposed animals	Incidence sham	n° sham animals	Species	Genetic Background
NTP 2018 (1) [32]	2.5	LTE	14	179	10	179	Mice	WT
NTP 2018 (2) [32]	5	LTE	16	180	10	179	Mice	WT
NTP 2018 (3) [32]	10	LTE	11	180	10	179	Mice	WT
NTP 2018 (4) [32]	2.5	LTE	12	179	10	179	Mice	WT
NTP 2018 (5) [32]	5	LTE	12	179	10	179	Mice	WT
NTP 2018 (6) [32]	10	LTE	8	179	10	179	Mice	WT
Oberto 2007 (1) [81]	0.5	LTE	2	100	0	100	Mice	Prone
Oberto 2007 (2) [81]	1.4	LTE	2	100	0	100	Mice	Prone
Oberto 2007 (3) [81]	4	LTE	4	100	0	100	Mice	Prone
Tillmann 2007 (1) [84]	0.29	LTE	10	100	9	100	Mice	WT
Tillmann 2007 (2) [84]	0.86	LTE	9	100	9	100	Mice	WT
Tillmann 2007 (3) [84]	2.6	LTE	12	100	9	100	Mice	WT
Tillmann 2007 (4) [84]	0.29	LTE	5	100	14	100	Mice	WT
Tillmann 2007 (5) [84]	0.86	LTE	7	100	14	100	Mice	WT
Tillmann 2007 (6) [84]	2.6	LTE	8	100	14	100	Mice	WT

Table S1.29. Uro-Genital male Benign

Paper (treated/sham comparison)	SAR	Duration	Incidence exposed	n° exposed animals	Incidence sham	n° sham animals	Species	Genetic Background
Anderson 2004 (1) [75]	0.16	LTE	79	90	79	90	Rats	WT
Anderson 2004 (1) [75]	1.6	LTE	80	90	79	90	Rats	WT
Chou 1992 [63]	0.15-0.4	LTE	2	101	1	101	Rats	WT
La Regina 2003 (1) [76]	1.3 ± 0.5	LTE	76	80	80	80	Rats	WT
La Regina 2003 (2) [76]	1.3 ± 0.5	LTE	72	80	80	80	Rats	WT
NTP 2018 (1) M [31]	1.5	LTE	4	90	4	90	Rats	WT
NTP 2018 (2) M [31]	3	LTE	9	90	4	90	Rats	WT
NTP 2018 (3) M [31]	6	LTE	5	90	4	90	Rats	WT
NTP 2018 (4) M [31]	1.5	LTE	2	90	4	90	Rats	WT
NTP 2018 (5) M [31]	3	LTE	4	90	4	90	Rats	WT
NTP 2018 (6) M [31]	6	LTE	2	90	4	90	Rats	WT
NTP 2018 (1) M [32]	2.5	LTE	2	90	3	90	Mice	WT
NTP 2018 (2) M [32]	5	LTE	0	90	3	90	Mice	WT
NTP 2018 (3) M [32]	10	LTE	0	90	3	90	Mice	WT
NTP 2018 (4) M [32]	2.5	LTE	2	90	3	90	Mice	WT
NTP 2018 (5) M [32]	5	LTE	1	91	3	90	Mice	WT
NTP 2018 (6) M [32]	10	LTE	1	91	3	90	Mice	WT

Table S1.30. Uro-Genital female Benign

Paper (treated/sham comparison)	SAR	Duration	Incidence exposed	n° exposed animals	Incidence sham	n° sham animals	Species	Genetic Background
Frei et al 1998 b [66]	1	LTE	14	100	15	99	Mice	Prone
Frei et al 1998 a [65]	0.3	LTE	12	97	9	92	Mice	Prone
La Regina 2003 (1) [76]	1.3 ± 0.4	LTE	12	80	12	80	Rats	WT
La Regina 2003 (2) [76]	1.3 ± 0.5	LTE	14	80	12	80	Rats	WT
NTP 2018 (1) F [31]	1.5	LTE	22	90	19	90	Rats	WT
NTP 2018 (2) F [31]	3	LTE	13	90	19	90	Rats	WT
NTP 2018 (3) F [31]	6	LTE	19	90	19	90	Rats	WT
NTP 2018 (4) F [31]	1.5	LTE	18	90	19	90	Rats	WT
NTP 2018 (5) F [31]	3	LTE	18	90	19	90	Rats	WT
NTP 2018 (6) F [31]	6	LTE	19	90	19	90	Rats	WT
NTP 2018 (1) F [32]	2.5	LTE	6	90	5	89	Mice	WT
NTP 2018 (2) F [32]	5	LTE	8	90	5	89	Mice	WT
NTP 2018 (3) F [32]	10	LTE	10	89	5	89	Mice	WT
NTP 2018 (4) F [32]	2.5	LTE	2	89	5	89	Mice	WT
NTP 2018 (5) F [32]	5	LTE	11	88	5	89	Mice	WT
NTP 2018 (6) F [32]	10	LTE	8	90	5	89	Mice	WT
Smith 2007 (1) [83]	0.27	LTE	7	50	10	50	Mice	Prone
Smith 2007 (2) [83]	0.8	LTE	6	49	10	50	Mice	WT
Smith 2007 (3) [83]	2.42	LTE	12	50	10	50	Mice	WT
Smith 2007 (4) [83]	0.29	LTE	5	50	8	50	Mice	WT
Smith 2007 (5) [83]	0.87	LTE	6	50	8	50	Mice	WT
Smith 2007 (6) [83]	2.61	LTE	7	50	8	50	Mice	WT
Toler 1997 [74]	0.32	LTE	4	187	5	172	Mice	WT
Tillmann 2007 (1) [84]	0.29	LTE	9	50	1	50	Mice	Prone
Tillmann 2007 (2) [84]	0.86	LTE	4	50	1	50	Mice	Prone
Tillmann 2007 (3) [84]	2.6	LTE	4	50	1	50	Rats	WT
Tillmann 2007 (4) [84]	0.29	LTE	5	50	5	50	Rats	WT
Tillmann 2007 (5) [84]	0.86	LTE	3	50	5	50	Rats	WT
Tillmann 2007 (6) [84]	2.6	LTE	5	50	5	50	Rats	WT

Table S1.31. Intestine Benign

Paper (treated/sham comparison)	SAR	Duration	Incidence exposed	n° exposed animals	Incidence sham	n° sham animals	Species	Genetic Background
NTP 2018 (1) [31]	1.5	LTE	0	171	1	173	Rats	WT
NTP 2018 (2) [31]	3	LTE	0	174	1	173	Rats	WT
NTP 2018 (3) [31]	6	LTE	0	176	1	173	Rats	WT
NTP 2018 (4) [31]	1.5	LTE	0	173	1	173	Rats	WT
NTP 2018 (5) [31]	3	LTE	0	171	1	173	Rats	WT
NTP 2018 (6) [31]	6	LTE	0	164	1	173	Rats	WT
NTP 2018 (1) [32]	2.5	LTE	1	171	0	172	Mice	WT
NTP 2018 (2) [32]	5	LTE	2	174	0	172	Mice	WT
NTP 2018 (3) [32]	10	LTE	0	170	0	172	Mice	WT
NTP 2018 (4) [32]	2.5	LTE	1	174	0	172	Mice	WT
NTP 2018 (5) [32]	5	LTE	2	166	0	172	Mice	WT
NTP 2018 (6) [32]	10	LTE	3	171	0	172	Mice	WT

Table S1.32. Kidney Benign

Paper (treated/sham comparison)	SAR	Duration	Incidence exposed	n° exposed animals	Incidence sham	n° sham animals	Species	Genetic Background
Chou 1992 [63]	0.15-0.4	LTE	1	100	1	100	Rats	WT
NTP 2018 (1) [31]	1.5	LTE	4	180	6	180	Rats	WT
NTP 2018 (2) [31]	3	LTE	4	180	6	180	Rats	WT
NTP 2018 (3) [31]	6	LTE	3	179	6	180	Rats	WT
NTP 2018 (4) [31]	1.5	LTE	3	180	6	180	Rats	WT
NTP 2018 (5) [31]	3	LTE	3	180	6	180	Rats	WT
NTP 2018 (6) [31]	6	LTE	0	176	6	180	Rats	WT
NTP 2018 (1) [32]	2.5	LTE	1	176	2	179	Mice	WT
NTP 2018 (2) [32]	5	LTE	1	179	2	179	Mice	WT
NTP 2018 (3) [32]	10	LTE	0	177	2	179	Mice	WT
NTP 2018 (4) [32]	2.5	LTE	0	178	2	179	Mice	WT
NTP 2018 (5) [32]	5	LTE	0	178	2	179	Mice	WT
NTP 2018 (6) [32]	10	LTE	1	177	2	179	Mice	WT
Tillmann 2010 [73]	1.5-5	LTE	1	56	0	54	Mice	WT

Table S1.33. Liver Benign

Paper (treated/sham comparison)	SAR	Duration	Incidence exposed	n° exposed animals	Incidence sham	n° sham animals	Species	Genetic Background
Chou 1992 [63]	0.15-0.4	LTE	3	100	0	100	Rats	WT
Frei et al 1998 b [66]	1	LTE	12	100	13	100	Mice	Prone
Frei et al 1998 a [65]	0.3	LTE	10	99	9	93	Mice	Prone
La Regina 2003 (1) [76]	1.3 ± 0.5	LTE	0	160	1	160	Rats	WT
La Regina 2003 (2) [76]	1.3 ± 0.5	LTE	3	160	1	160	Rats	WT
NTP 2018 (1) [31]	1.5	LTE	4	180	7	180	Rats	WT
NTP 2018 (2) [31]	3	LTE	1	180	7	180	Rats	WT
NTP 2018 (3) [31]	6	LTE	5	180	7	180	Rats	WT
NTP 2018 (4) [31]	1.5	LTE	4	180	7	180	Rats	WT
NTP 2018 (5) [31]	3	LTE	6	179	7	180	Rats	WT
NTP 2018 (6) [31]	6	LTE	1	178	7	180	Rats	WT
NTP 2018 (1) [32]	2.5	LTE	79	179	71	179	Mice	WT
NTP 2018 (2) [32]	5	LTE	80	180	71	179	Mice	WT
NTP 2018 (3) [32]	10	LTE	65	179	71	179	Mice	WT
NTP 2018 (4) [32]	2.5	LTE	90	177	71	179	Mice	WT
NTP 2018 (5) [32]	5	LTE	77	180	71	179	Mice	WT
NTP 2018 (6) [32]	10	LTE	82	180	71	179	Mice	WT
Oberto 2007 (1) [81]	0.5	LTE	2	100	0	100	Mice	Prone
Oberto 2007 (2) [81]	1.4	LTE	0	100	0	100	Mice	Prone
Oberto 2007 (3) [81]	4	LTE	0	100	0	100	Mice	Prone
Tillmann 2007 (1) [84]	0.29	LTE	11	100	17	100	Mice	WT
Tillmann 2007 (2) [84]	0.86	LTE	12	100	17	100	Mice	WT
Tillmann 2007 (3) [84]	2.6	LTE	9	100	17	100	Mice	WT
Tillmann 2007 (4) [84]	0.29	LTE	13	100	13	100	Mice	WT
Tillmann 2007 (5) [84]	0.86	LTE	11	100	13	100	Mice	WT
Tillmann 2007 (6) [84]	2.6	LTE	5	100	13	100	Mice	WT
Tillmann 2010 [73]	1.5-5	LTE	48	56	38	54	Mice	WT
Toler 1997 [74]	0.32	LTE	100	188	94	180	Mice	Prone
**Excluded papers**								
De Seze 2020 [64]	0.83	LTE	0	24	2	23	Rats	WT
Jauchem 2001 [67]	0.01	MTE	10	100	8	100	Mice	Prone

Table S1.34. Lung Benign

Paper (treated/sham comparison)	SAR	Duration	Incidence exposed	n° exposed animals	Incidence sham	n° sham animals	Species	Genetic Background
Frei et al 1998 b [66]	1	LTE	3	100	4	100	Mice	Prone
Frei et al 1998 a [65]	0.3	LTE	0	99	4	97	Mice	Prone
NTP 2018 (1) [31]	1.5	LTE	1	180	1	180	Rats	WT
NTP 2018 (2) [31]	3	LTE	0	180	1	180	Rats	WT
NTP 2018 (3) [31]	6	LTE	0	180	1	180	Rats	WT
NTP 2018 (4) [31]	1.5	LTE	2	180	1	180	Rats	WT
NTP 2018 (5) [31]	3	LTE	1	180	1	180	Rats	WT
NTP 2018 (6) [31]	6	LTE	0	180	1	180	Rats	WT
NTP 2018 (1) [32]	2.5	LTE	19	180	16	180	Mice	WT
NTP 2018 (2) [32]	5	LTE	25	180	16	180	Mice	WT
NTP 2018 (3) [32]	10	LTE	18	180	16	180	Mice	WT
NTP 2018 (4) [32]	2.5	LTE	14	179	16	180	Mice	WT
NTP 2018 (5) [32]	5	LTE	20	180	16	180	Mice	WT
NTP 2018 (6) [32]	10	LTE	14	180	16	180	Mice	WT
Oberto 2007 (1) [81]	0.5	LTE	2	100	3	100	Mice	Prone
Oberto 2007 (2) [81]	1.4	LTE	1	100	3	100	Mice	Prone
Oberto 2007 (3) [81]	4	LTE	4	100	3	100	Mice	Prone
Tillmann 2007 (1) [84]	0.29	LTE	14	100	17	100	Mice	WT
Tillmann 2007 (2) [84]	0.86	LTE	15	100	17	100	Mice	WT
Tillmann 2007 (3) [84]	2.6	LTE	13	100	17	100	Mice	WT
Tillmann 2007 (4) [84]	0.29	LTE	9	100	9	100	Mice	WT
Tillmann 2007 (5) [84]	0.86	LTE	7	100	9	100	Mice	WT
Tillmann 2007 (6) [84]	2.6	LTE	11	100	9	100	Mice	WT
Tillmann 2010 [73]	1.5-5	LTE	2	56	2	54	Mice	WT
**Excluded papers**								
Jauchem 2001 [67]	0.01	MTE	4	100	3	100	Mice	Prone

Table S1.35. Mammary Benign

Paper (treated/sham comparison)	SAR	Duration	Incidence exposed	n° exposed animals	Incidence sham	n° sham animals	Species	Genetic Background
Anderson 2004 (1) [75]	0.16	LTE	40	180	26	180	Rats	WT
Anderson 2004 (2) [75]	1.6	LTE	29	180	26	180	Rats	WT
Frei et al 1998 a [65]	0.3	LTE	2	96	1	89	Mice	Prone
Jin 2010 [68]	4	LTE	2	41	1	41	Rats	WT
La Regina 2003 (1) [76]	1.3 ± 0.5	LTE	9	160	8	160	Rats	WT
La Regina 2003 (2) [76]	1.3 ± 0.5	LTE	12	160	8	160	Rats	WT
NTP 2018 (1) [31]	1.5	LTE	76	165	73	172	Rats	WT
NTP 2018 (2) [31]	3	LTE	66	171	73	172	Rats	WT
NTP 2018 (3) [31]	6	LTE	67	172	73	172	Rats	WT
NTP 2018 (4) [31]	1.5	LTE	70	167	73	172	Rats	WT
NTP 2018 (5) [31]	3	LTE	66	170	73	172	Rats	WT
NTP 2018 (6) [31]	6	LTE	65	170	73	172	Rats	WT
NTP 2018 (1) [32]	2.5	LTE	0	93	0	87	Mice	WT
NTP 2018 (2) [32]	5	LTE	0	90	0	87	Mice	WT
NTP 2018 (3) [32]	10	LTE	0	92	0	87	Mice	WT
NTP 2018 (4) [32]	2.5	LTE	1	88	0	87	Mice	WT
NTP 2018 (5) [32]	5	LTE	2	90	0	87	Mice	WT
NTP 2018 (6) [32]	10	LTE	0	91	0	87	Mice	WT
Smith 2007 (1) [83]	0.27	LTE	16	100	11	100	Rats	WT
Smith 2007 (2) [83]	0.8	LTE	12	100	11	100	Rats	WT
Smith 2007 (3) [83]	2.42	LTE	12	100	11	100	Rats	WT
Smith 2007 (4) [83]	0.29	LTE	14	100	18	100	Rats	WT
Smith 2007 (5) [83]	0.87	LTE	15	100	18	100	Rats	WT
Smith 2007 (6) [83]	2.61	LTE	17	99	18	100	Rats	WT
Szmigielski 1982 [78] (1)	2.5	MTE	32	40	14	40	Mice	Prone
Szmigielski 1982 [78] (1)	7	MTE	37	40	14	40	Mice	Prone
**Excluded papers**								
Jauchem 2001 [67]	0.01	MTE	1	100	1	100	Mice	Prone
De Seze 2020 [64]	0.83	STE	1	12	0	24	Rats	WT

Table S1.36. Pancreas benign

Paper (treated/sham comparison)	SAR	Duration	Incidence exposed	n° exposed animals	Incidence sham	n° sham animals	Species	Genetic Background
Chou 1992 [63]	0.15-0.4	LTE	1	100	2	100	Rats	WT
Frei et al 1998 b [66]	1	LTE	2	100	1	99	Mice	Prone
Frei et al 1998 a [65]	0.3	LTE	1	95	0	93	Mice	Prone
La Regina 2003 (1) [76]	1.3 ± 0.5	LTE	4	160	7	160	Rats	WT
La Regina 2003 (2) [76]	1.3 ± 0.5	LTE	2	160	7	160	Rats	WT
NTP 2018 (1) [31]	1.5	LTE	39	178	28	180	Rats	WT
NTP 2018 (2) [31]	3	LTE	31	178	28	180	Rats	WT
NTP 2018 (3) [31]	6	LTE	21	173	28	180	Rats	WT
NTP 2018 (4) [31]	1.5	LTE	37	178	28	180	Rats	WT
NTP 2018 (5) [31]	3	LTE	39	177	28	180	Rats	WT
NTP 2018 (6) [31]	6	LTE	18	167	28	180	Rats	WT
NTP 2018 (1) [32]	2.5	LTE	0	178	0	175	Mice	WT
NTP 2018 (2) [32]	5	LTE	1	180	0	175	Mice	WT
NTP 2018 (3) [32]	10	LTE	3	177	0	175	Mice	WT
NTP 2018 (4) [32]	2.5	LTE	1	178	0	175	Mice	WT
NTP 2018 (5) [32]	5	LTE	1	178	0	175	Mice	WT
NTP 2018 (6) [32]	10	LTE	1	176	0	175	Mice	WT
Smith 2007 (1) [83]	0.27	LTE	4	100	5	100	Rats	WT
Smith 2007 (2) [83]	0.8	LTE	4	100	5	100	Rats	WT
Smith 2007 (3) [83]	2.42	LTE	1	100	5	100	Rats	WT
Smith 2007 (4) [83]	0.29	LTE	3	100	3	100	Rats	WT
Smith 2007 (5) [83]	0.87	LTE	1	100	3	100	Rats	WT
Smith 2007 (6) [83]	2.61	LTE	3	100	3	100	Rats	WT
**Excluded papers**								
De Seze 2020 [64]	0.83	STE	1	6	3	23	Rats	WT
Jauchem 2001 [67]	0.01	MTE	2	100	2	100	Mice	Prone

Table S1.37. Pituitary Benign

Paper (treated/sham comparison)	SAR	Duration	Incidence exposed	n° exposed animals	Incidence sham	n° sham animals	Species	Genetic Background
Chou 1992 [63]	0.15-0.4	LTE	17	100	21	100	Rats	WT
Frei et al 1998 a [65]	0.3	LTE	1	95	0	90	Mice	Prone
La Regina 2003 (1) [76]	1.3 ± 0.5	LTE	21	160	26	160	Rats	WT
La Regina 2003 (2) [76]	1.3 ± 0.5	LTE	25	160	26	160	Rats	WT
NTP 2018 (1) [31]	1.5	LTE	61	180	60	179	Rats	WT
NTP 2018 (2) [31]	3	LTE	64	180	60	179	Rats	WT
NTP 2018 (3) [31]	6	LTE	58	180	60	179	Rats	WT
NTP 2018 (4) [31]	1.5	LTE	66	179	60	179	Rats	WT
NTP 2018 (5) [31]	3	LTE	64	179	60	179	Rats	WT
NTP 2018 (6) [31]	6	LTE	53	180	60	179	Rats	WT
NTP 2018 (1) [32]	2.5	LTE	5	165	6	166	Mice	WT
NTP 2018 (2) [32]	5	LTE	7	171	6	166	Mice	WT
NTP 2018 (3) [32]	10	LTE	5	169	6	166	Mice	WT
NTP 2018 (4) [32]	2.5	LTE	8	163	6	166	Mice	WT
NTP 2018 (5) [32]	5	LTE	10	177	6	166	Mice	WT
NTP 2018 (6) [32]	10	LTE	1	169	6	166	Mice	WT
Oberto 2007 (1) [81]	0.5	LTE	11	100	7	100	Mice	Prone
Oberto 2007 (2) [81]	1.4	LTE	12	100	7	100	Mice	Prone
Oberto 2007 (3) [81]	4	LTE	11	100	7	100	Mice	Prone
Smith 2007 (1) [83]	0.27	LTE	37	100	44	100	Rats	WT
Smith 2007 (2) [83]	0.8	LTE	49	100	44	100	Rats	WT
Smith 2007 (3) [83]	2.42	LTE	47	100	44	100	Rats	WT
Smith 2007 (4) [83]	0.29	LTE	50	100	44	100	Rats	WT
Smith 2007 (5) [83]	0.87	LTE	44	100	44	100	Rats	WT
Smith 2007 (6) [83]	2.61	LTE	44	100	44	100	Rats	WT
Tillmann 2007 (1) [84]	0.29	LTE	10	100	9	100	Mice	WT
Tillmann 2007 (2) [84]	0.86	LTE	9	99	9	100	Mice	WT
Tillmann 2007 (3) [84]	2.6	LTE	10	100	9	100	Mice	WT
Tillmann 2007 (4) [84]	0.29	LTE	11	100	6	100	Mice	WT
Tillmann 2007 (5) [84]	0.86	LTE	12	99	6	99	Mice	WT
Tillmann 2007 (6) [84]	2.6	LTE	6	100	6	100	Mice	WT
**Excluded papers**								
De Seze 2020 [64]	0.83	STE	5	24	4	23	Rats	WT

Table S1.38. Skin Benign

Paper (treated/sham comparison)	SAR	Duration	Incidence exposed	n° exposed animals	Incidence sham	n° sham animals	Species	Genetic Background
Chou 1992 [63]	0.15-0.4	LTE	7	100	2	100	Rats	WT
NTP 2018 (1) [31]	1.5	LTE	15	180	15	180	Rats	WT
NTP 2018 (2) [31]	3	LTE	22	180	15	180	Rats	WT
NTP 2018 (3) [31]	6	LTE	16	180	15	180	Rats	WT
NTP 2018 (4) [31]	1.5	LTE	23	180	15	180	Rats	WT
NTP 2018 (5) [31]	3	LTE	24	180	15	180	Rats	WT
NTP 2018 (6) [31]	6	LTE	18	180	15	180	Rats	WT
NTP 2018 (1) [32]	2.5	LTE	1	180	4	180	Mice	WT
NTP 2018 (2) [32]	5	LTE	2	180	4	180	Mice	WT
NTP 2018 (3) [32]	10	LTE	0	180	4	180	Mice	WT
NTP 2018 (4) [32]	2.5	LTE	0	180	4	180	Mice	WT
NTP 2018 (5) [32]	5	LTE	1	180	4	180	Mice	WT
NTP 2018 (6) [32]	10	LTE	2	180	4	180	Mice	WT
**Excluded papers**								
De Seze 2020 [64]	0.83	STE	3	8	6	23	Rats	WT

Table S1.39. Stomach Benign

Paper (treated/sham comparison)	SAR	Duration	Incidence exposed	n° exposed animals	Incidence sham	n° sham animals	Species	Genetic Background
Chou 1992 [63]	0.15-0.4	LTE	3	100	4	100	Rats	WT
Frei et al 1998 a [65]	0.3	LTE	1	100	2	94	Mice	Prone
NTP 2018 (1) [31]	1.5	LTE	0	180	1	180	Rats	WT
NTP 2018 (2) [31]	3	LTE	1	180	1	180	Rats	WT
NTP 2018 (3) [31]	6	LTE	0	180	1	180	Rats	WT
NTP 2018 (4) [31]	1.5	LTE	0	180	1	180	Rats	WT
NTP 2018 (5) [31]	3	LTE	0	179	1	180	Rats	WT
NTP 2018 (6) [31]	6	LTE	1	180	1	180	Rats	WT
NTP 2018 (1) [32]	2.5	LTE	1	176	1	174	Mice	WT
NTP 2018 (2) [32]	5	LTE	2	179	1	174	Mice	WT
NTP 2018 (3) [32]	10	LTE	0	172	1	174	Mice	WT
NTP 2018 (4) [32]	2.5	LTE	1	177	1	174	Mice	WT
NTP 2018 (5) [32]	5	LTE	1	173	1	174	Mice	WT
NTP 2018 (6) [32]	10	LTE	1	174	1	174	Mice	WT

Table S1.40. Thymus Benign

Paper (treated/sham comparison)	SAR	Duration	Incidence exposed	n° exposed animals	Incidence sham	n° sham animals	Species	Genetic Background
NTP 2018 (1) [31]	1.5	LTE	3	172	1	175	Rats	WT
NTP 2018 (2) [31]	3	LTE	1	176	1	175	Rats	WT
NTP 2018 (3) [31]	6	LTE	0	172	1	175	Rats	WT
NTP 2018 (4) [31]	1.5	LTE	0	168	1	175	Rats	WT
NTP 2018 (5) [31]	3	LTE	1	174	1	175	Rats	WT
NTP 2018 (6) [31]	6	LTE	0	169	1	175	Rats	WT
NTP 2018 (1) [32]	1.5	LTE	0	163	0	160	Mice	WT
NTP 2018 (2) [32]	5	LTE	1	165	0	160	Mice	WT
NTP 2018 (3) [32]	10	LTE	0	158	0	160	Mice	WT
Smith 2007 (1) [83]	0.27	LTE	6	97	8	98	Rats	WT
Smith 2007 (2) [83]	0.8	LTE	6	99	8	98	Rats	WT
Smith 2007 (3) [83]	2.42	LTE	5	99	8	98	Rats	WT
Smith 2007 (4) [83]	0.29	LTE	8	100	6	94	Rats	WT
Smith 2007 (5) [83]	0.87	LTE	3	97	6	94	Rats	WT
Smith 2007 (6) [83]	2.61	LTE	6	95	6	94	Rats	WT

Table S1.41. Thyroid Benign

Paper (treated/sham comparison)	SAR	Duration	Incidence exposed	n° exposed animals	Incidence sham	n° sham animals	Species	Genetic Background
Anderson 2004 (1) [75]	0.16	LTE	17	180	25	180	Rats	WT
Anderson 2004 (2) [75]	1.6	LTE	17	180	25	180	Rats	WT
Chou 1992 [63]	0.15-0.4	LTE	10	100	9	100	Rats	WT
Frei et al 1998 a [65]	0.3	LTE	1	84	0	86	Mice	Prone
La Regina 2003 (1) [76]	1.3 ± 0.5	LTE	10	160	8	160	Rats	WT
La Regina 2003 (2) [76]	1.3 ± 0.5	LTE	13	160	8	160	Rats	WT
NTP 2018 (1) [31]	1.5	LTE	22	177	14	179	Rats	WT
NTP 2018 (2) [31]	3	LTE	17	179	14	179	Rats	WT
NTP 2018 (3) [31]	6	LTE	21	175	14	179	Rats	WT
NTP 2018 (4) [31]	1.5	LTE	23	177	14	179	Rats	WT
NTP 2018 (5) [31]	3	LTE	18	176	14	179	Rats	WT
NTP 2018 (6) [31]	6	LTE	20	174	14	179	Rats	WT
NTP 2018 (1) [32]	2.5	LTE	0	177	0	175	Mice	WT
NTP 2018 (2) [32]	5	LTE	0	174	0	175	Mice	WT
NTP 2018 (3) [32]	10	LTE	0	174	0	175	Mice	WT
NTP 2018 (4) [32]	2.5	LTE	3	176	0	175	Mice	WT
NTP 2018 (5) [32]	5	LTE	2	176	0	175	Mice	WT
NTP 2018 (6) [32]	10	LTE	2	175	0	175	Mice	WT
Smith 2007 (1) [83]	0.27	LTE	14	100	14	100	Rats	WT
Smith 2007 (2) [83]	0.8	LTE	12	100	14	100	Rats	WT
Smith 2007 (3) [83]	2.42	LTE	18	100	14	100	Rats	WT
Smith 2007 (4) [83]	0.29	LTE	8	100	13	100	Rats	WT
Smith 2007 (5) [83]	0.87	LTE	13	100	13	100	Rats	WT
Smith 2007 (6) [83]	2.61	LTE	15	100	13	100	Rats	WT
