# Supplementary material for: In Vivo Studies on Radiofrequency (100 kHz–300 GHz) Electromagnetic Field Exposure and Cancer: A Systematic Review
Source: Int J Environ Res Public Health. 2023 Jan 23;20(3):2071. doi: 10.3390/ijerph20032071 (PMC9915925; doi:10.3390/ijerph20032071)
Supplement: Supplementary file 1 [file ijerph-20-02071-s001.zip › File S2.pdf]

International Journal of
Environmental Research and Public Health

MDPI

Supplementary Material 2

Incidence data and results of meta-analysis. Malignant tumors from Figure S2.1 to Figure S2.25; benign tumors from Figure S2.26 to Figure S2.41

Figure S2.1 Adrenal Malignant

		exposed		sham					
ID Study	study name	events	total number	events	total number	RR	lower limit	upper limit	weight %
1	Chou 1992 [63]	3	100	1	100	3.000	0.313	28.748	4.04%
2	Frei et al 1998 b [66]	1	98	2	99	0.505	0.046	5.563	3.59%
3	Frei et al 1998a [65]	0	95	1	93	0.326	0.013	8.078	2.01%
4	La Regina 2003 (1) [76]	1	160	3	160	0.333	0.035	3.198	4.02%
5	La Regina 2003 (2) [76]	1	160	3	160	0.333	0.035	3.198	4.02%
6	NTP 2018 (1) [31]	4	180	3	180	1.333	0.301	5.902	9.28%
7	NTP 2018 (2) [31]	5	179	3	180	1.676	0.405	6.942	10.17%
8	NTP 2018 (3) [31]	1	178	3	180	0.337	0.035	3.235	4.02%
9	NTP 2018 (4) [31]	8	180	3	180	2.667	0.716	9.934	11.87%
10	NTP 2018 (5) [31]	6	180	3	180	2.000	0.506	7.911	10.86%
11	NTP 2018 (6) [31]	3	180	3	180	1.000	0.203	4.915	8.10%
12	NTP 2018^ (1) [32]	0	177	2	174	0.197	0.009	4.109	2.22%
13	NTP 2018^ (2) [32]	0	179	2	174	0.194	0.009	4.064	2.22%
14	NTP 2018^ (3) [32]	0	178	2	174	0.196	0.009	4.086	2.22%
15	NTP 2018^ (4) [32]	0	177	2	174	0.197	0.009	4.109	2.22%
16	NTP 2018^ (5) [32]	1	177	2	174	0.492	0.045	5.416	3.57%
17	NTP 2018^ (6) [32]	0	177	2	174	0.197	0.009	4.109	2.22%
18	Tillmann 2007 (1) [84]	0	100	0	100	1.000	0.020	51.124	1.33%
19	Tillmann 2007 (2) [84]	0	100	0	100	1.000	0.020	51.124	1.33%
20	Tillmann 2007 (3) [84]	1	100	0	100	3.000	0.121	74.212	2.01%
21	Tillmann 2007 (4) [84]	0	100	0	100	1.000	0.020	51.124	1.33%
22	Tillmann 2007 (5) [84]	1	100	0	100	3.000	0.121	74.212	2.01%
23	Tillmann 2007 (6) [84]	0	100	0	100	1.000	0.020	51.124	1.33%
24	Toler 1997 [74]	3	183	1	175	2.869	0.299	27.530	4.02%
	**Combined Effect Size**					**1.016**	**1.510**	**0.680**	
	**Heterogeneity**	**Tau = 0**	**I^2 = 0**						
	**Overall effect**	**z = 0.085**	**(p = 0.932)**						

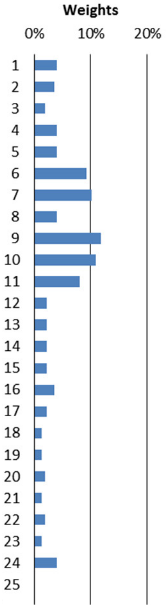

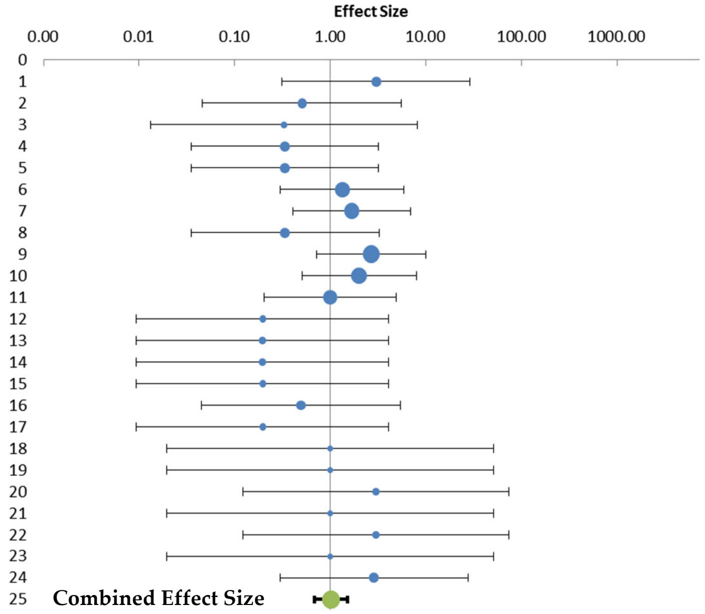

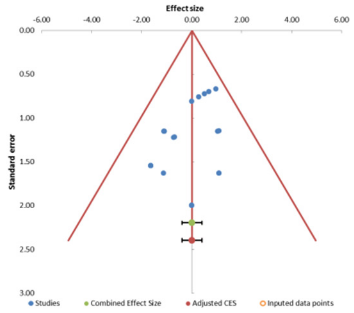

NTP 2018 [31] indicates NTP rat study, NTP 2018^ [32] indicates NTP mice study

Figure S2.2 Bladder Malignant

		exposed		sham					
ID Study	study name	events	total number	events	total number	RR	lower limit	upper limit	weight %
1	Chou 1992 [63]	1	100	0	100	3.000	0.121	74.212	7.46%
2	La Regina 2003 (1) [76	0	160	0	160	1.000	0.020	50.845	4.95%
3	La Regina 2003 (2) [76	1	160	0	160	3.000	0.122	73.992	7.44%
4	NTP 2018 (1) [31]	1	177	1	177	1.000	0.062	16.015	9.93%
5	NTP 2018 (2) [31]	0	176	1	177	0.335	0.014	8.264	7.44%
6	NTP 2018 (3) [31]	0	172	1	177	0.343	0.014	8.456	7.44%
7	NTP 2018 (4) [31]	0	171	1	177	0.345	0.014	8.505	7.44%
8	NTP 2018 (5) [31]	0	173	1	177	0.341	0.014	8.407	7.44%
9	NTP 2018 (6) [31]	0	168	1	177	0.351	0.014	8.656	7.44%
10	NTP 2018^ (1) [32]	2	175	0	173	4.943	0.237	103.313	8.27%
11	NTP 2018^ (2) [32]	0	176	0	173	0.983	0.019	49.946	4.95%
12	NTP 2018^ (3) [32]	0	175	0	173	0.989	0.019	50.231	4.95%
13	NTP 2018^ (4) [32]	0	171	0	173	1.012	0.020	51.404	4.95%
14	NTP 2018^ (5) [32]	0	173	0	173	1.000	0.020	50.811	4.95%
15	NTP 2018^ (6) [32]	0	168	0	173	1.030	0.020	52.321	4.95%
	**Combined Effect Size**					**0.904**	**0.540**	**1.512**	
	**Heterogeneity**	**Tau = 0**	**I^2 = 0**						
	**Overall effect**	**z = -0.423**	**(p = 0.672)**						

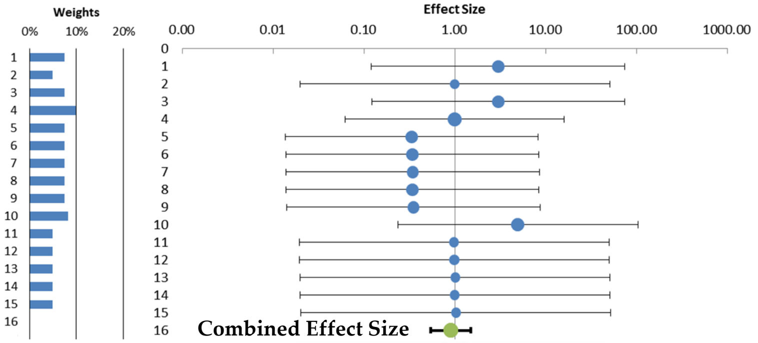

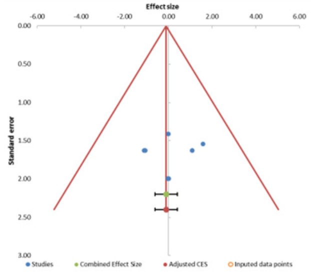

NTP 2018 [31] indicates NTP rat study, NTP 2018^ [32] indicates NTP mice study

Figure S2.3 Histiocytic Sarcoma

		exposed		sham					
ID Study	study name	events	total number	events	total number	RR	lower limit	upper limit	weight %
1	Chou 1992 [63]	1	100	0	100	3.000	0.121	74.212	0.97%
2	La Regina 2003 (1) [76	1	160	0	160	3.000	0.122	73.992	0.96%
3	La Regina 2003 (2) [76	0	160	0	160	1.000	0.020	50.845	0.64%
4	NTP 2018 (1) [31]	1	180	0	180	3.000	0.122	73.952	0.96%
5	NTP 2018 (2) [31]	0	180	0	180	1.000	0.020	50.795	0.64%
6	NTP 2018 (3) [31]	1	180	0	180	3.000	0.122	73.952	0.96%
7	NTP 2018 (4) [31]	0	180	0	180	1.000	0.020	50.795	0.64%
8	NTP 2018 (5) [31]	2	180	0	180	5.000	0.239	104.489	1.07%
9	NTP 2018 (6) [31]	1	180	0	180	3.000	0.122	73.952	0.96%
10	NTP 2018^ (1) [32]	2	179	8	180	0.251	0.054	1.174	4.17%
11	NTP 2018^ (2) [32]	9	180	8	180	1.125	0.443	2.859	11.38%
12	NTP 2018^ (3) [32]	7	180	8	180	0.875	0.323	2.370	9.97%
13	NTP 2018^ (4) [32]	5	180	8	180	0.625	0.208	1.881	8.16%
14	NTP 2018^ (5) [32]	3	180	8	180	0.375	0.101	1.397	5.72%
15	NTP 2018^ (6) [32]	9	180	8	180	1.125	0.443	2.859	11.38%
16	Oberto 2007 (1) [81]	11	100	7	100	1.571	0.631	3.911	11.98%
17	Oberto 2007 (2) [81]	5	100	7	100	0.714	0.233	2.190	7.93%
18	Oberto 2007 (3) [81]	5	100	7	100	0.714	0.233	2.190	7.93%
19	Tillmann 2007 (1) [84]	3	100	1	100	3.000	0.313	28.748	1.95%
20	Tillmann 2007 (2) [84]	2	100	1	100	2.000	0.182	22.026	1.73%
21	Tillmann 2007 (3) [84]	3	100	1	100	3.000	0.313	28.748	1.95%
22	Tillmann 2007 (4) [84]	5	100	1	100	5.000	0.587	42.589	2.17%
23	Tillmann 2007 (5) [84]	0	100	1	100	0.333	0.013	8.246	0.97%
24	Tillmann 2007 (6) [84]	2	100	1	100	2.000	0.182	22.026	1.73%
25	Tillmann 2010 [73]	0	56	3	54	0.138	0.007	2.696	1.14%
26	Toler 1997 [74]	1	189	3	181	0.319	0.033	3.063	1.94%
	**Combined Effect Size**					**0.979**	**0.735**	**1.305**	
	**Heterogeneity**	**Tau = 0**	**I^2= 0**						
	**Overall effect**	**z = -0.151**	**(p = 0.880)**						

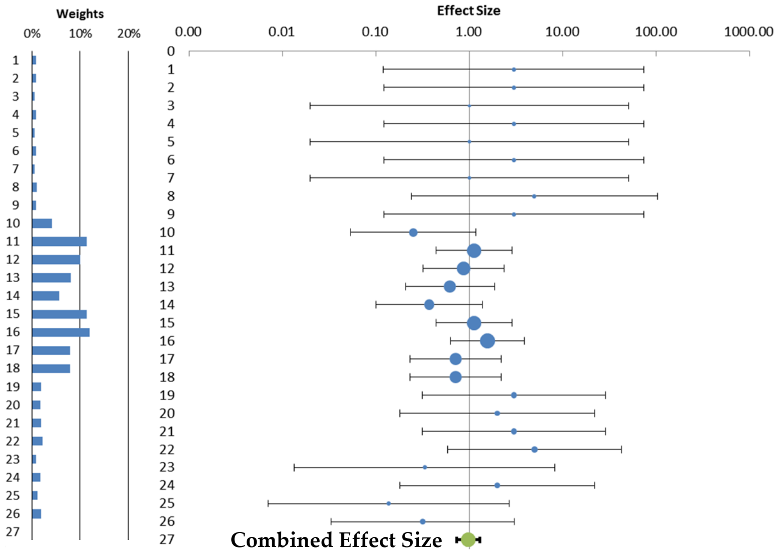

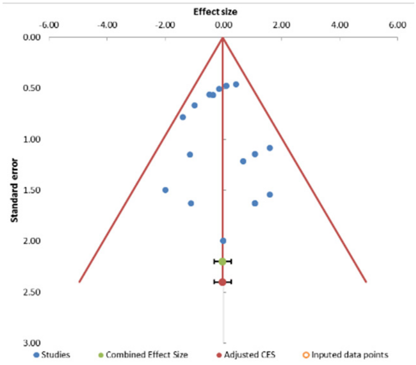

NTP 2018 [31] indicates NTP rat study, NTP 2018^ [32] indicates NTP mice study

Figure S2.4 Bone Marrow Malignant

		exposed		sham					
ID Study	study name	events	total number	events	total number	RR	lower limit	upper limit	weight %
1	Frei et al 1998a [65]	0	100	1	99	0.330	0.013	8.164	8.25%
2	NTP 2018^ (1) [32]	1	178	2	180	0.506	0.046	5.572	14.66%
3	NTP 2018^ (2) [32]	0	179	2	180	0.201	0.010	4.203	9.14%
4	NTP 2018^ (3) [32]	0	180	2	180	0.200	0.010	4.180	9.14%
5	NTP 2018^ (4) [32]	2	179	2	180	1.006	0.142	7.108	22.08%
6	NTP 2018^ (5) [32]	1	180	2	180	0.500	0.045	5.510	14.66%
7	NTP 2018^ (6) [32]	2	179	2	180	1.006	0.142	7.108	22.08%
	**Combined Effect Size**					**0.558**	**0.304**	**1.024**	
	**Heterogeneity**	**Tau = 0**	**I^2 = 0**						
	**Overall effect**	**z = -2.351**	**(p = 0.019)**						

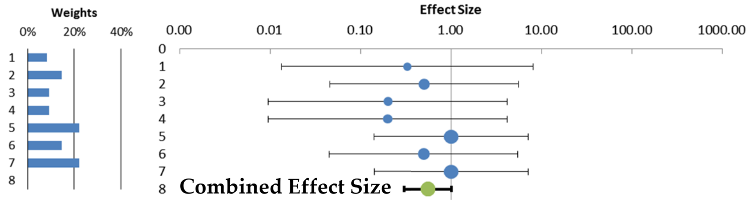

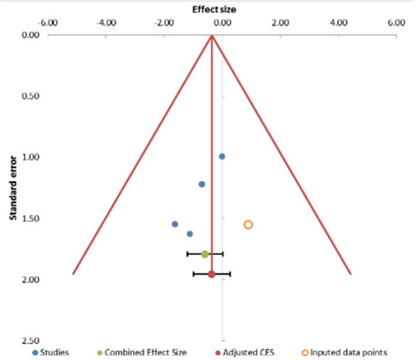

NTP 2018^ [32] indicates NTP mice study

Figure S2.5 CNS Malignant

ID	Study study name	exposed events	exposed total number	sham events	sham total number	RR	lower limit	upper limit	weight %
1	Adey 1999 [62]	2	60	7	60	0.286	0.061	1.341	3.07%
2	Adey 2000 [61]	4	90	1	90	4.000	0.449	35.620	1.52%
3	Anderson 2004 (1) [75]	3	180	4	180	0.750	0.169	3.320	3.27%
4	Anderson 2004 (2) [75]	6	180	4	180	1.500	0.429	5.248	4.61%
5	Falcioni 2018 (1) [80]	11	811	4	817	2.770	0.885	8.672	5.53%
6	Falcioni 2018 (2) [80]	5	411	4	817	2.485	0.670	9.216	4.19%
7	Falcioni 2018 (3) [80]	3	409	4	817	1.498	0.336	6.672	3.23%
8	La Regina 2003 (1) [76]	2	160	2	160	1.000	0.142	7.065	1.89%
9	La Regina 2003 (2) [76]	2	160	2	160	1.000	0.142	7.065	1.89%
10	NTP 2018 (1) [31]	4	180	0	180	9.000	0.483	167.600	0.85%
11	NTP 2018 (2) [31]	5	180	0	180	11.000	0.607	199.419	0.86%
12	NTP 2018 (3) [31]	4	180	0	180	9.000	0.483	167.600	0.85%
13	NTP 2018 (4) [31]	7	180	0	180	15.000	0.855	263.229	0.88%
14	NTP 2018 (5) [31]	1	180	0	180	3.000	0.122	73.952	0.70%
15	NTP 2018 (6) [31]	4	180	0	180	9.000	0.483	167.600	0.85%
16	Sommer 2007 [71]	6	160	4	160	1.500	0.429	5.240	4.63%
17	Utteridge 2002 (1) [85]	17	120	11	120	1.545	0.753	3.171	14.06%
18	Utteridge 2002 (2) [85]	15	120	11	120	1.364	0.651	2.857	13.28%
19	Utteridge 2002 (3) [85]	10	120	11	120	0.909	0.399	2.069	10.74%
20	Utteridge 2002 (4) [85]	9	118	11	120	0.832	0.356	1.943	10.10%
21	Utteridge 2002 (5) [85]	4	120	1	120	4.000	0.449	35.661	1.52%
22	Utteridge 2002 (6) [85]	0	120	1	120	0.333	0.013	8.235	0.71%
23	Utteridge 2002 (7) [85]	2	120	1	120	2.000	0.182	22.031	1.26%
24	Utteridge 2002 (8) [85]	2	120	1	120	2.000	0.182	22.031	1.26%
25	Zook 2001 (1) [79]	5	60	3	60	1.667	0.411	6.760	3.74%
26	Zook 2001 (2) [79]	4	60	5	60	0.800	0.223	2.872	4.49%
	Combined Effect Size					1.405	1.073	1.839	
	Heterogeneity	Tau = 0	$I^2 = 0$						
	Overall effect	z = 2.596	(p = 0.009)						

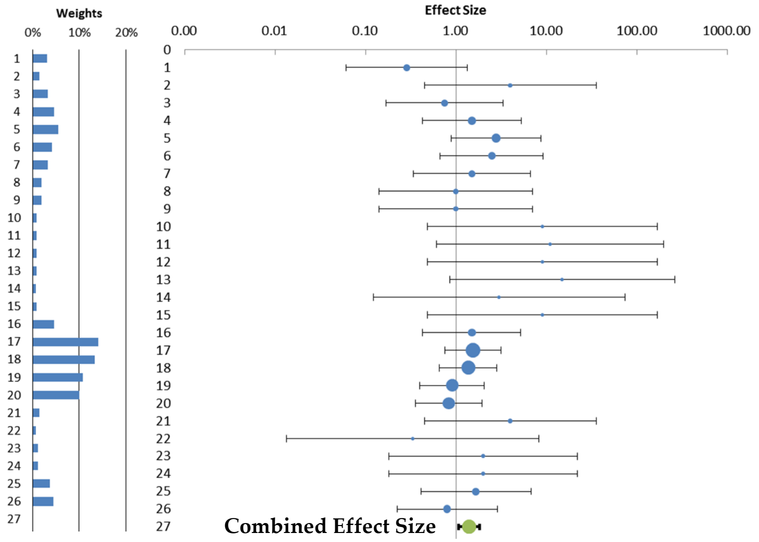

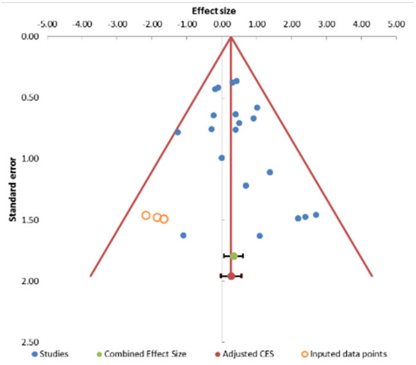

NTP 2018 [31] indicates NTP rat study

Figure S2.6 Brain Malignant

		exposed		sham					
**ID Study**	**study name**	**events**	**total number**	**events**	**total number**	**RR**	**lower limit**	**upper limit**	**weight %**
1	Adey 1999 [62]	2	60	5	60	0.400	0.079	2.015	2.86%
2	Adey 2000 [61]	3	90	1	90	3.000	0.313	28.738	1.45%
3	Anderson 2004 (1) [75]	3	180	4	180	0.750	0.169	3.320	3.33%
4	Anderson 2004 (2) [75]	6	180	4	180	1.500	0.429	5.248	4.70%
5	Falcioni 2018 (1) [80]	11	811	4	817	2.770	0.885	8.672	5.63%
6	Falcioni 2018 (2) [80]	5	411	4	817	2.485	0.670	9.216	4.27%
7	Falcioni 2018 (3) [80]	3	409	4	817	1.498	0.336	6.672	3.29%
8	La Regina 2003 (1) [76]	2	160	2	160	1.000	0.142	7.065	1.93%
9	La Regina 2003 (2) [76]	1	160	2	160	0.500	0.045	5.509	1.28%
10	NTP 2018 (1) [31]	3	180	0	180	7.039	0.363	136.661	0.84%
11	NTP 2018 (2) [31]	4	180	0	180	9.050	0.486	168.532	0.86%
12	NTP 2018 (3) [31]	4	180	0	180	9.000	0.483	167.600	0.86%
13	NTP 2018 (4) [31]	7	180	0	180	15.000	0.855	263.229	0.90%
14	NTP 2018 (5) [31]	1	180	0	180	3.000	0.122	73.952	0.72%
15	NTP 2018 (6) [31]	4	180	0	180	9.000	0.483	167.600	0.86%
16	Sommer 2007 [31]	6	160	4	160	1.500	0.429	5.240	4.71%
17	Utteridge 2002 (1) [85]	17	120	11	120	1.545	0.753	3.171	14.32%
18	Utteridge 2002 (2) [85]	15	120	11	120	1.364	0.651	2.857	13.52%
19	Utteridge 2002 (3) [85]	10	120	11	120	0.909	0.399	2.069	10.93%
20	Utteridge 2002 (4) [85]	9	118	11	120	0.832	0.356	1.943	10.29%
21	Utteridge 2002 (5) [85]	4	120	1	120	4.000	0.449	35.661	1.54%
22	Utteridge 2002 (6) [85]	0	120	1	120	0.333	0.013	8.235	0.72%
23	Utteridge 2002 (7) [85]	2	120	1	120	2.000	0.182	22.031	1.28%
24	Utteridge 2002 (8) [85]	2	120	1	120	2.000	0.182	22.031	1.28%
25	Zook 2001 (1) [79]	5	60	3	60	1.667	0.411	6.760	3.81%
26	Zook 2001 (2) [79]	3	60	5	60	0.600	0.148	2.434	3.81%
	**Combined Effect Size**					**1.392**	**1.072**	**1.807**	
	**Heterogeneity**	**Tau = 0**	**I^2= 0**						
	**Overall effect**	**z = 2.606**	**(p = 0.009)**						

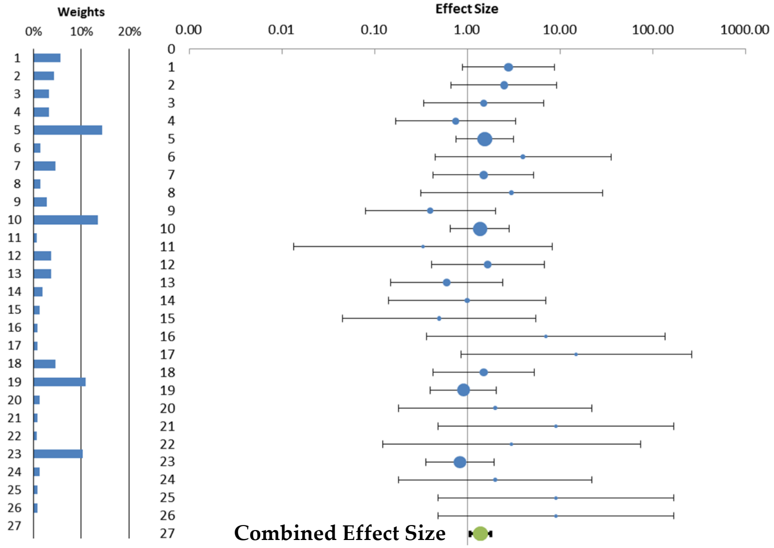

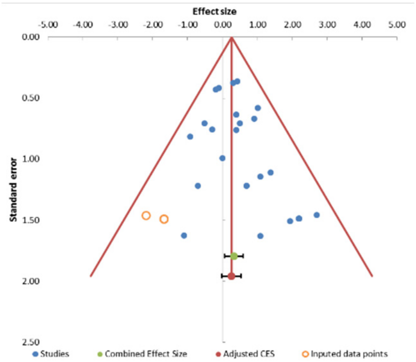

NTP 2018 [31] indicates NTP rat study

Figure S2.7 Sensorial System Malignant

		exposed		sham					
ID Study	study name	events	total number	events	total number	RR	lower limit	upper limit	weight %
1	La Regina 2003 (1) [76]	2	160	0	160	5,000	0,239	104,533	3,24%
2	La Regina 2003 (2) [76]	0	160	0	160	1,000	0,020	50,845	1,94%
3	NTP 2018 (1) [31]	0	180	0	173	0,961	0,019	48,838	1,94%
4	NTP 2018 (2) [31]	3	180	0	173	6,729	0,347	130,656	3,40%
5	NTP 2018 (3) [31]	2	180	0	173	4,807	0,230	100,454	3,23%
6	NTP 2018 (4) [31]	1	169	0	173	3,071	0,125	75,709	2,91%
7	NTP 2018 (5) [31]	0	169	0	173	1,024	0,020	52,012	1,94%
8	NTP 2018 (6) [31]	0	158	0	173	1,094	0,022	55,625	1,94%
9	NTP 2018^ (1) [32]	3	179	3	179	1,000	0,203	4,915	11,78%
10	NTP 2018^ (2) [32]	2	180	3	179	0,663	0,111	3,944	9,39%
11	NTP 2018^ (3) [32]	0	180	3	179	0,142	0,007	2,758	3,40%
12	NTP 2018^ (4) [32]	4	179	3	179	1,333	0,301	5,902	13,50%
13	NTP 2018^ (5) [32]	2	179	3	179	0,667	0,112	3,966	9,40%
14	NTP 2018^ (6) [32]	4	179	3	179	1,333	0,301	5,902	13,50%
15	Tillmann 2007 (1) [84]	1	100	0	100	3,000	0,121	74,212	2,92%
16	Tillmann 2007 (2) [84]	0	100	0	100	1,000	0,020	51,124	1,94%
17	Tillmann 2007 (3) [84]	0	100	0	100	1,000	0,020	51,124	1,94%
18	Tillmann 2007 (4) [84]	1	100	2	100	0,500	0,045	5,506	5,22%
19	Tillmann 2007 (5) [84]	0	101	2	100	0,198	0,009	4,150	3,25%
20	Tillmann 2007 (6) [84]	0	101	2	100	0,198	0,009	4,150	3,25%
	**Combined Effect Size**					**1,028**	**0,680**	**1,551**	
	**Heterogeneity**	**Tau = 0**	**I^2= 0**						
	**Overall effect**	**z = 0.138**	**(p = 0.890)**						

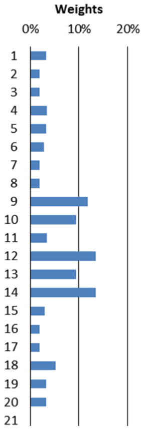

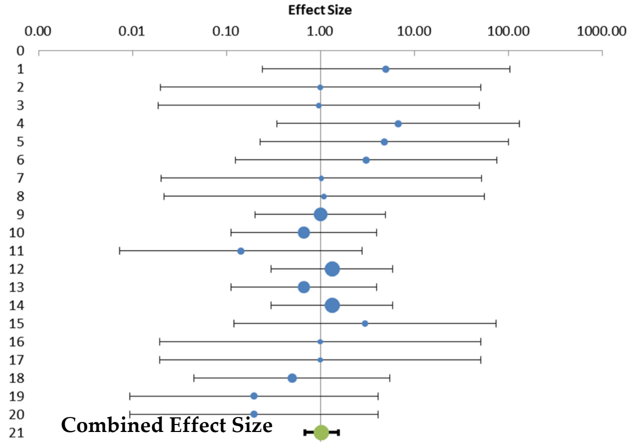

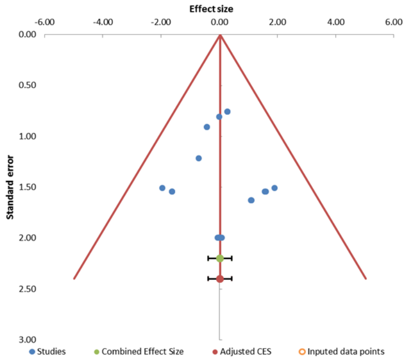

NTP 2018 [31] indicates NTP rat study, NTP 2018^ [32] indicates NTP mice study

Figure S2.8 Male Uro-genital System Malignant

ID Study	study name	exposed events	exposed total number	sham events	sham total number	RR	lower limit	upper limit	weight %
1	Anderson 2004 (1) [75]	1	90	0	90	3,000	0,121	74,279	6,16%
2	Anderson 2004 (1) [75]	6	90	0	90	13,000	0,729	231,891	7,65%
3	La Regina 2003 (1) [76]	5	80	3	80	1,667	0,408	6,814	32,07%
4	La Regina 2003 (2) [76]	3	80	3	80	1,000	0,206	4,865	25,40%
5	NTP 2018 (1)M [31]	0	90	0	90	1,000	0,020	51,208	4,10%
6	NTP 2018 (2)M [31]	1	90	0	90	3,000	0,121	74,279	6,16%
7	NTP 2018 (3)M [31]	1	90	0	90	3,000	0,121	74,279	6,16%
8	NTP 2018 (4)M [31]	0	90	0	90	1,000	0,020	51,208	4,10%
9	NTP 2018 (5)M [31]	0	90	0	90	1,000	0,020	51,208	4,10%
10	NTP 2018 (6)M [31]	0	90	0	90	1,000	0,020	51,208	4,10%
	**Combined Effect Size**					**1,756**	**1,034**	**2,982**	
	**Heterogeneity**	**Tau =0**	**I^2 = 0**						
	**Overall effect**	**z = 2.406**	**(p = 0.016)**						

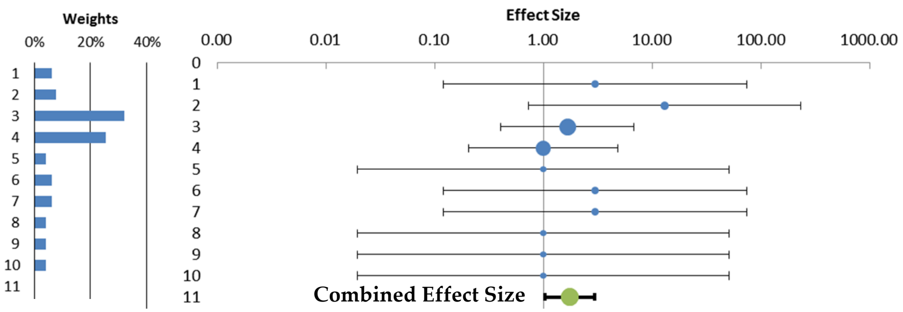

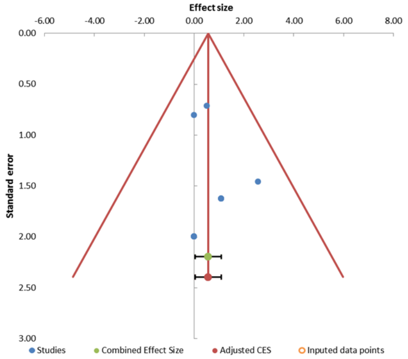

NTP 2018 [31] indicates NTP rat study

Figure S2.9 Female Uro-genital System Malignant

ID Study	study name	exposed events	exposed total number	sham events	sham total number	RR	lower limit	upper limit	weight %
1	Frei et al 1998 b [66]	6	100	5	99	1,188	0,372	3,793	3,53%
2	Frei et al 1998a [65]	2	98	3	92	0,626	0,106	3,703	1,51%
3	La Regina 2003 (1) [76]	1	80	0	80	3,000	0,121	74,363	0,46%
4	La Regina 2003 (2) [76]	0	80	0	80	1,000	0,019	51,314	0,31%
5	NTP 2018 (1)F [31]	4	90	11	90	0,364	0,119	1,108	3,84%
6	NTP 2018 (2)F [31]	10	90	11	90	0,909	0,404	2,045	7,25%
7	NTP 2018 (3)F [31]	9	90	11	90	0,818	0,354	1,889	6,81%
8	NTP 2018 (4)F [31]	2	90	11	90	0,182	0,041	0,805	2,15%
9	NTP 2018 (5)F [31]	6	90	11	90	0,545	0,209	1,421	5,20%
10	NTP 2018 (6)F [31]	5	90	11	90	0,455	0,163	1,264	4,55%
11	NTP 2018^ (1)F [32]	3	90	3	89	0,989	0,203	4,821	1,90%
12	NTP 2018^ (2)F [32]	1	90	3	89	0,330	0,034	3,158	0,93%
13	NTP 2018^ (3)F [32]	1	89	3	89	0,333	0,035	3,193	0,93%
14	NTP 2018^ (4)F [32]	5	84	3	89	1,766	0,431	7,232	2,40%
15	NTP 2018^ (5)F [32]	1	84	3	89	0,353	0,037	3,383	0,93%
16	NTP 2018^ (6)F [32]	5	83	3	89	1,787	0,436	7,319	2,40%
17	Oberto 2007 (1) [81]	2	50	1	50	2,000	0,182	21,999	0,84%
18	Oberto 2007 (2) [81]	1	50	1	50	1,000	0,062	16,091	0,62%
19	Oberto 2007 (3) [81]	2	50	1	50	2,000	0,182	21,999	0,84%
20	Smith 2007 (1) [83]	1	50	0	50	3,000	0,120	74,837	0,47%
21	Smith 2007 (2) [83]	1	49	0	50	3,060	0,123	76,347	0,47%
22	Smith 2007 (3) [83]	1	50	0	50	3,000	0,120	74,837	0,47%
23	Smith 2007 (4) [83]	0	50	0	50	1,000	0,019	51,906	0,31%
24	Smith 2007 (5) [83]	1	50	0	50	3,000	0,120	74,837	0,47%
25	Smith 2007 (6) [83]	2	50	0	50	5,000	0,237	105,482	0,52%
26	Tillmann 2007 (1) [84]	2	50	2	50	1,000	0,143	6,989	1,27%
27	Tillmann 2007 (2) [84]	6	50	2	50	3,000	0,624	14,434	1,95%
28	Tillmann 2007 (3) [84]	2	50	2	50	1,000	0,143	6,989	1,27%
29	Tillmann 2007 (4) [84]	0	50	2	50	0,200	0,009	4,219	0,52%
30	Tillmann 2007 (5) [84]	1	50	2	50	0,500	0,045	5,500	0,84%
31	Tillmann 2007 (6) [84]	0	50	2	50	0,200	0,009	4,219	0,52%
32	Toler 1997 [74]	53	187	49	172	0,995	0,715	1,383	43,52%
	**Combined Effect Size**					**0,882**	**0,723**	**1,076**	
	**Heterogeneity**	Tau = 0	$I^2 = 0$						
	**Overall effect**	z = -1.284	(p = 0.199)						

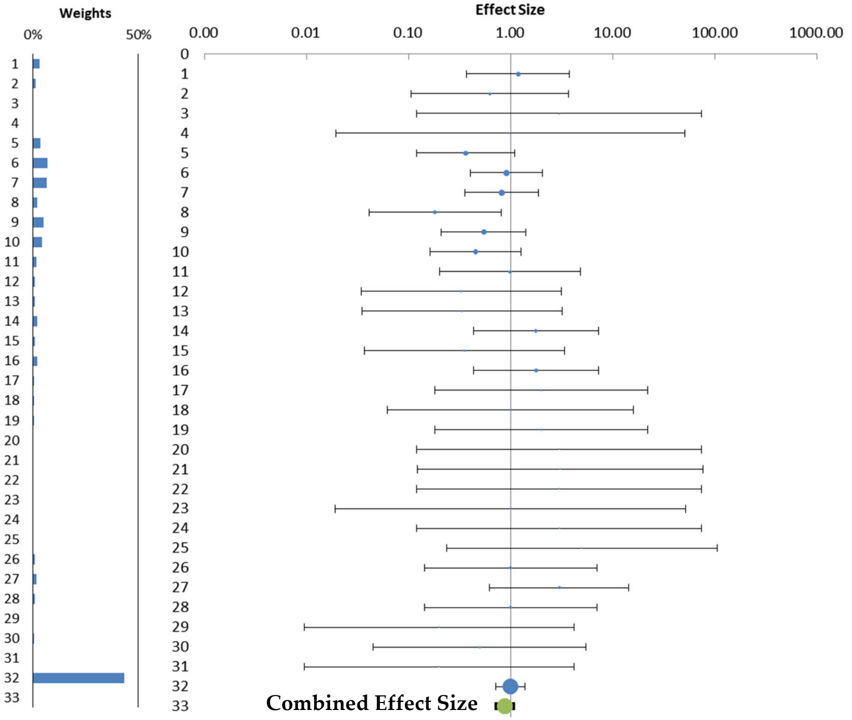

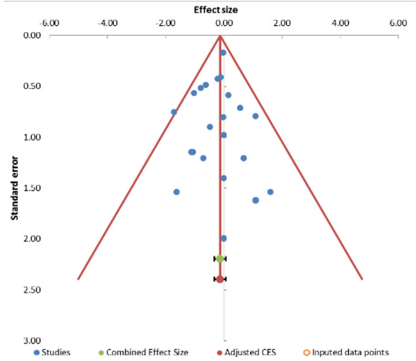

NTP 2018 [31] indicates NTP rat study, NTP 2018^ [32] indicates NTP mice study

Figure S2.10 Heart Malignant

		exposed		sham					
ID Study	study name	events	total number	events	total number	RR	lower limit	upper limit	weight %
1	Falcioni 2018 (1) [80]	12	811	4	817	3,022	0,978	9,339	26,65%
2	Falcioni 2018 (2) [80]	2	411	4	817	0,994	0,182	5,413	11,81%
3	Falcioni 2018 (3) [80]	5	409	4	817	2,497	0,673	9,261	19,75%
4	NTP 2018 (1) [31]	4	180	0	180	9,000	0,483	167,600	3,99%
5	NTP 2018 (2) [31]	4	180	0	180	9,000	0,483	167,600	3,99%
6	NTP 2018 (3) [31]	5	180	0	180	11,000	0,607	199,419	4,06%
7	NTP 2018 (4) [31]	4	180	0	180	9,000	0,483	167,600	3,99%
8	NTP 2018 (5) [31]	3	180	0	180	7,000	0,361	135,905	3,88%
9	NTP 2018 (6) [31]	8	180	0	180	17,000	0,979	295,178	4,19%
10	NTP 2018^ (1) [32]	1	179	0	180	3,017	0,122	74,364	3,32%
11	NTP 2018^ (2) [32]	1	180	0	180	3,000	0,122	73,952	3,32%
12	NTP 2018^ (3) [32]	1	180	0	180	3,000	0,122	73,952	3,32%
13	NTP 2018^ (4) [32]	1	180	0	180	3,000	0,122	73,952	3,32%
14	NTP 2018^ (5) [32]	0	180	0	180	1,000	0,020	50,795	2,21%
15	NTP 2018^ (6) [32]	0	180	0	180	1,000	0,020	50,795	2,21%
	**Combined Effect Size**					**3,238**	**2,105**	**4,983**	
	**Heterogeneity**	**Tau = 0**	**I^2= 0**						
	**Overall effect**	**z = 5.849**	**(p<<0.01)**						

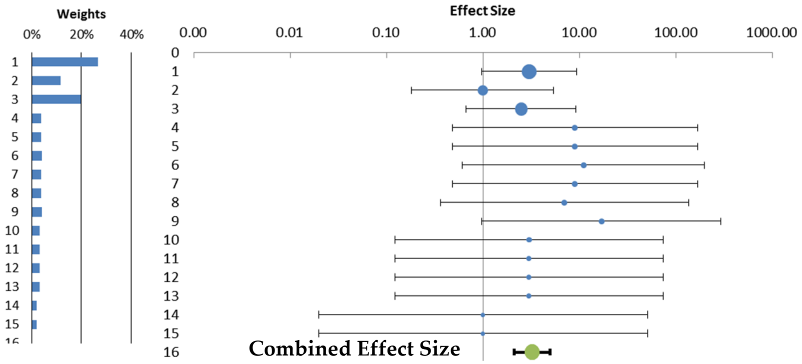

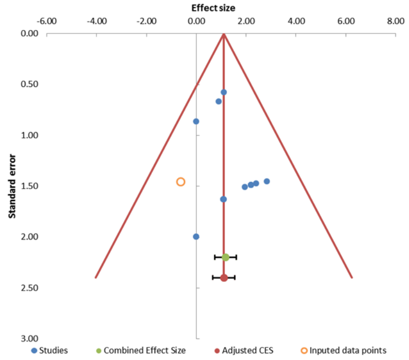

NTP 2018 [31] indicates NTP rat study, NTP 2018^ [32] indicates NTP mice study

Figure S2.11 Intestine Malignant

ID Study	study name	exposed events	exposed total number	sham events	sham total number	RR	lower limit	upper limit	weight %
1	La Regina 2003 (1) [76]	1	160	1	160	1,000	0,062	16,018	3,93%
2	La Regina 2003 (2) [76]	0	160	1	160	0,333	0,014	8,221	2,95%
3	NTP 2018 (1) [31]	5	171	3	173	1,686	0,407	6,981	14,99%
4	NTP 2018 (2) [31]	1	174	3	173	0,331	0,035	3,180	5,92%
5	NTP 2018 (3) [31]	2	176	3	173	0,655	0,110	3,898	9,52%
6	NTP 2018 (4) [31]	1	173	3	173	0,333	0,035	3,199	5,92%
7	NTP 2018 (5) [31]	2	171	3	173	0,674	0,113	4,011	9,52%
8	NTP 2018 (6) [31]	0	164	3	173	0,151	0,008	2,925	3,44%
9	NTP 2018^ (1) [32]	1	171	3	172	0,335	0,035	3,217	5,92%
10	NTP 2018^ (2) [32]	0	174	3	172	0,141	0,007	2,742	3,44%
11	NTP 2018^ (3) [32]	2	170	3	172	0,675	0,113	4,012	9,52%
12	NTP 2018^ (4) [32]	1	174	3	172	0,330	0,034	3,162	5,92%
13	NTP 2018^ (5) [32]	2	168	3	172	0,683	0,115	4,059	9,52%
14	NTP 2018^ (6) [32]	2	170	3	172	0,675	0,113	4,012	9,52%
	**Combined Effect Size**					**0,585**	**0,399**	**0,857**	
	**Heterogeneity**	**Tau = 0**	**$I^2 = 0$**						
	**Overall effect**	**z = -3.033**	**(p = 0.002)**						

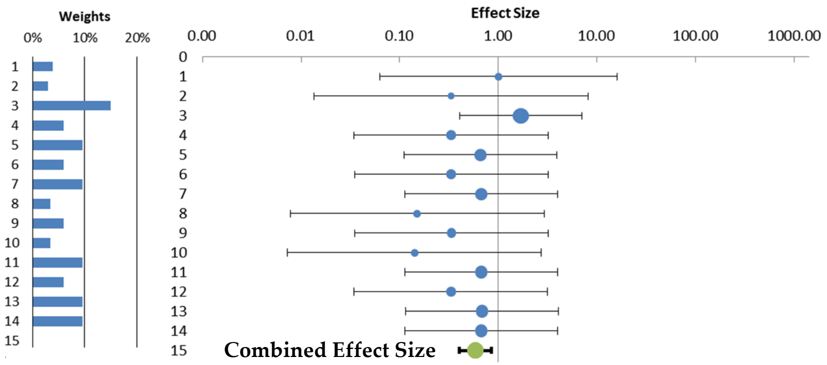

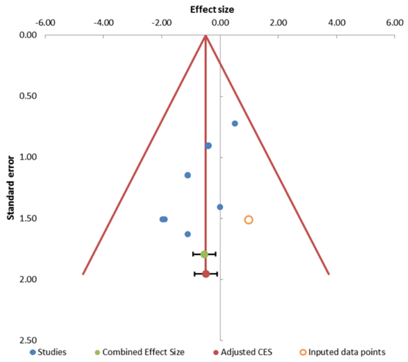

NTP 2018 [31] indicates NTP rat study, NTP 2018^ [32] indicates NTP mice study

Figure S2.12 Kidney Malignant

		exposed		sham					
ID Study	study name	events	total number	events	total number	RR	lower limit	upper limit	weight %
1	La Regina 2003 (1) [76]	2	160	0	160	5,000	0,239	104,533	4,64%
2	La Regina 2003 (1) [76]	0	160	0	160	1,000	0,020	50,845	2,78%
3	NTP 2018 (1) [31]	3	180	2	180	1,500	0,252	8,924	13,48%
4	NTP 2018 (2) [31]	1	180	2	180	0,500	0,045	5,510	7,44%
5	NTP 2018 (3) [31]	3	179	2	180	1,508	0,254	8,973	13,48%
6	NTP 2018 (4) [31]	0	180	2	180	0,200	0,010	4,180	4,64%
7	NTP 2018 (5) [31]	4	180	2	180	2,000	0,369	10,844	15,00%
8	NTP 2018 (6) [31]	3	176	2	180	1,534	0,258	9,126	13,48%
9	NTP 2018^ (1) [32]	0	176	1	179	0,339	0,014	8,357	4,17%
10	NTP 2018^ (2) [32]	0	179	1	179	0,333	0,014	8,217	4,17%
11	NTP 2018^ (3) [32]	0	177	1	179	0,337	0,014	8,310	4,17%
12	NTP 2018^ (4) [32]	0	178	1	179	0,335	0,014	8,263	4,17%
13	NTP 2018^ (5) [32]	0	178	1	179	0,335	0,014	8,263	4,17%
14	NTP 2018^ (6) [32]	0	177	1	179	0,337	0,014	8,310	4,17%
	**Combined Effect Size**					**0,949**	**0,572**	**1,573**	
	**Heterogeneity**	**Tau = 0**	**I^2 = 0**						
	**Overall effect**	**z = -0.226**	**(p = 0.821)**						

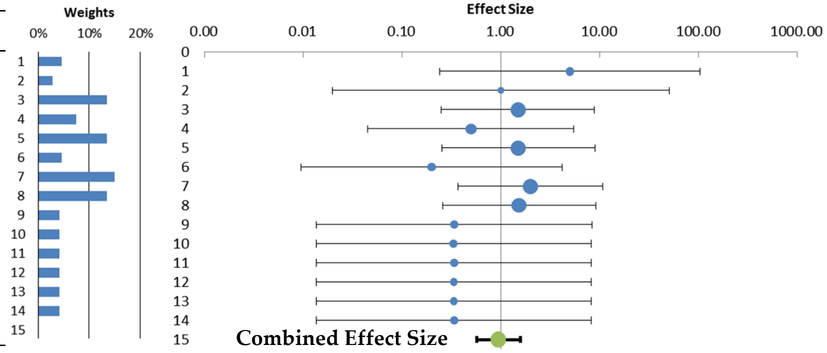

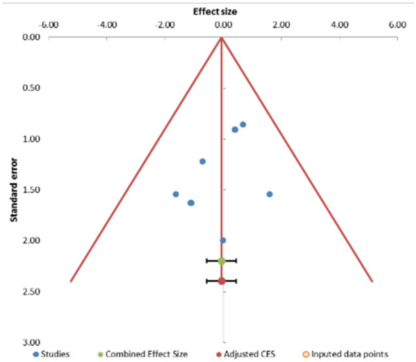

NTP 2018 [31] indicates NTP rat study, NTP 2018^ [32] indicates NTP mice study

Figure S2.13 Leukemia

ID Study	study name	exposed events	exposed total number	sham events	sham total number	RR	lower limit	upper limit	weight %
1	Anderson 2004 (1) [75]	45	180	62	180	0,726	0,525	1,004	22,65%
2	Anderson 2004 (2) [75]	46	180	62	180	0,742	0,538	1,023	22,99%
3	Chou 1992 [63]	2	100	1	100	2,000	0,182	22,026	0,49%
4	La Regina 2003 (1) [76]	62	160	57	160	1,088	0,817	1,448	27,86%
5	La Regina 2003 (2) [76]	45	160	57	160	0,789	0,570	1,093	22,63%
6	NTP 2018 (1) [31]	1	180	0	180	3,000	0,122	73,952	0,28%
7	NTP 2018 (2) [31]	1	180	0	180	3,000	0,122	73,952	0,28%
8	NTP 2018 (3) [31]	1	180	0	180	3,000	0,122	73,952	0,28%
9	NTP 2018 (4) [31]	5	180	0	180	11,000	0,607	199,419	0,34%
10	NTP 2018 (5) [31]	4	180	0	180	9,000	0,483	167,600	0,33%
11	NTP 2018 (6) [31]	1	180	0	180	3,000	0,122	73,952	0,28%
12	NTP 2018^ (1) [32]	1	179	0	180	3,017	0,122	74,364	0,28%
13	NTP 2018^ (2) [32]	0	180	0	180	1,000	0,020	50,795	0,18%
14	NTP 2018^ (3) [32]	1	180	0	180	3,000	0,122	73,952	0,28%
15	NTP 2018^ (4) [32]	1	180	0	180	3,000	0,122	73,952	0,003
16	NTP 2018^ (5) [32]	2	180	0	180	5,000	0,239	104,489	0,003
17	NTP 2018^ (6) [32]	1	180	0	180	3,000	0,122	73,952	0,003
	**Combined Effect Size**					**0,884**	**0,738**	**1,059**	
	**Heterogeneity**	**Tau = 0.072**	**I^2= 0.039**						
	**Overall effect**	**z = -1.450**	**(p = 0.147)**						

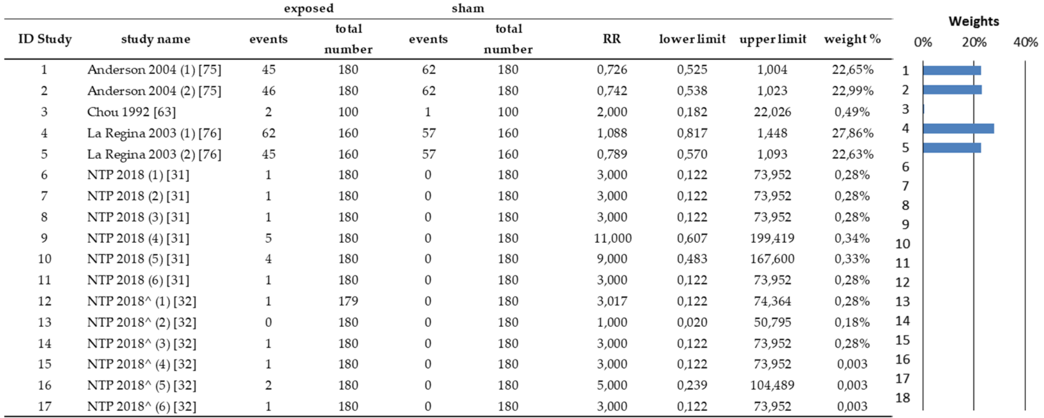

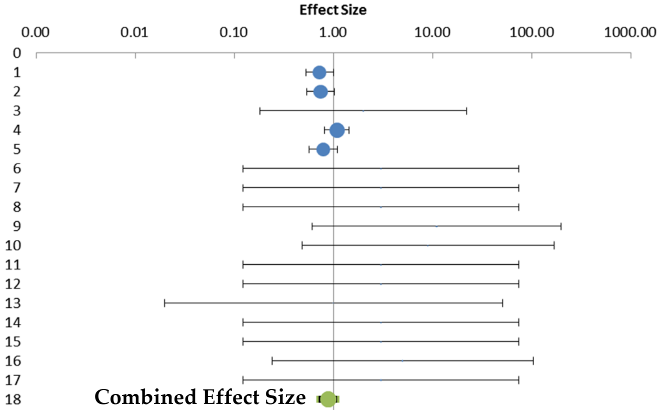

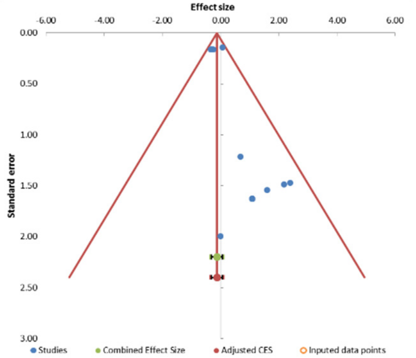

NTP 2018 [31] indicates NTP rat study, NTP 2018^ [32] indicates NTP mice study

Figure S2.14 Liver Malignant

ID Study	study name	exposed events	exposed total number	sham events	sham total number	RR	lower limit	upper limit	weight %
1	Chou 1992 [63]	0	100	1	100	0,333	0,013	8,246	0,17%
2	Frei et al 1998a [65]	1	99	3	93	0,313	0,033	3,000	0,35%
3	NTP 2018 (1) [31]	1	180	0	180	3,000	0,122	73,952	0,17%
4	NTP 2018 (2) [31]	1	180	0	180	3,000	0,122	73,952	0,17%
5	NTP 2018 (3) [31]	0	180	0	180	1,000	0,020	50,795	0,11%
6	NTP 2018 (4) [31]	0	179	0	180	1,006	0,020	51,078	0,11%
7	NTP 2018 (5) [31]	1	180	0	180	3,000	0,122	73,952	0,17%
8	NTP 2018 (6) [31]	2	180	0	180	5,000	0,239	104,489	0,19%
9	NTP 2018^ (1) [32]	42	179	45	179	0,933	0,647	1,347	13,09%
10	NTP 2018^ (2) [32]	49	180	45	179	1,083	0,764	1,535	14,49%
11	NTP 2018^ (3) [32]	32	179	45	179	0,711	0,475	1,066	10,79%
12	NTP 2018^ (4) [32]	36	177	45	179	0,809	0,549	1,192	11,77%
13	NTP 2018^ (5) [32]	49	180	45	179	1,083	0,764	1,535	14,49%
14	NTP 2018^ (6) [32]	46	180	45	179	1,017	0,712	1,452	13,90%
15	Oberto 2007 (1) [81]	0	100	1	100	0,333	0,013	8,246	0,17%
16	Oberto 2007 (2) [81]	0	100	1	100	0,333	0,013	8,246	0,17%
17	Oberto 2007 (3) [81]	5	100	1	100	5,000	0,587	42,589	0,39%
18	Tillmann 2007 (1) [84]	8	100	10	100	0,800	0,328	1,954	2,23%
19	Tillmann 2007 (2) [84]	8	100	10	100	0,800	0,328	1,954	2,23%
20	Tillmann 2007 (3) [84]	11	100	10	100	1,100	0,487	2,486	2,67%
21	Tillmann 2007 (4) [84]	9	100	9	100	1,000	0,412	2,427	2,26%
22	Tillmann 2007 (5) [84]	5	100	9	100	0,556	0,192	1,610	1,57%
23	Tillmann 2007 (6) [84]	10	100	9	100	1,111	0,469	2,631	2,39%
24	Tillmann 2010 [73]	13	56	8	54	1,567	0,700	3,510	2,76%
25	Toler 1997 [74]	13	188	13	180	0,957	0,455	2,014	3,19%
	**Combined Effect Size**					**0,958**	**0,861**	**1,066**	
	**Heterogeneity**	**Tau = 0**	**$I^2 = 0$**						
	**Overall effect**	**z = -0.835**	**(p = 0.403)**						

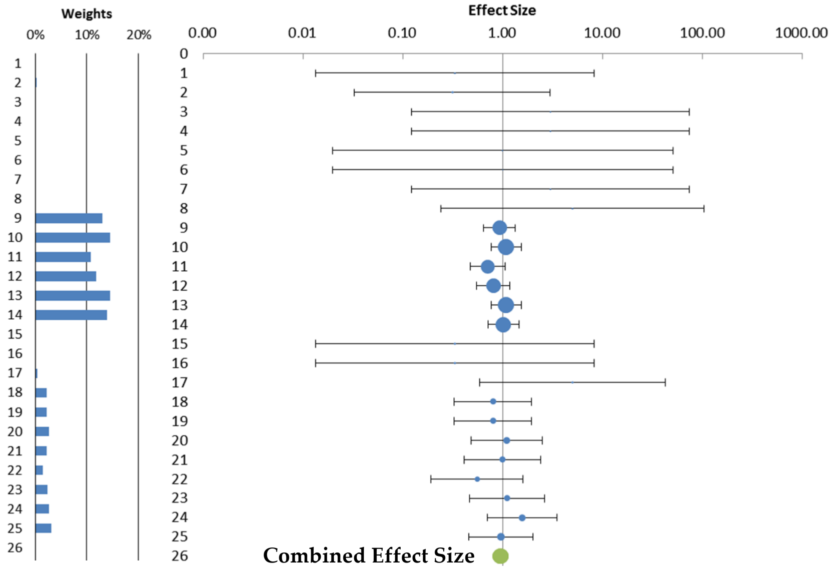

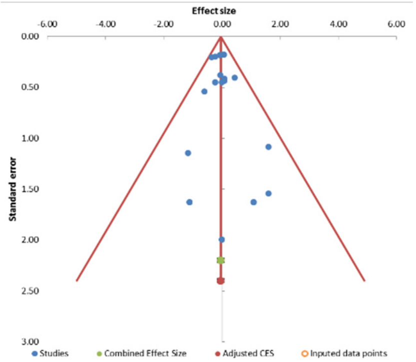

NTP 2018 [31] indicates NTP rat study, NTP 2018^ [32] indicates NTP mice study

Figure S2.15 Lung Malignant

		exposed		sham					
ID Study	study name	events	total number	events	total number	RR	lower limit	upper limit	weight %
1	Frei et al 1998a [65]	1	99	1	97	0,980	0,061	15,714	0,59%
2	La Regina 2003 (1) [76]	0	160	3	160	0,143	0,007	2,775	0,51%
3	La Regina 2003 (2) [76]	2	160	3	160	0,667	0,112	3,963	1,42%
4	NTP 2018 (1) [31]	2	180	0	180	5,000	0,239	104,489	0,49%
5	NTP 2018 (2) [31]	0	180	0	180	1,000	0,020	50,795	0,29%
6	NTP 2018 (3) [31]	0	180	0	180	1,000	0,020	50,795	0,29%
7	NTP 2018 (4) [31]	0	180	0	180	1,000	0,020	50,795	0,29%
8	NTP 2018 (5) [31]	1	180	0	180	3,000	0,122	73,952	0,44%
9	NTP 2018 (6) [31]	0	180	0	180	1,000	0,020	50,795	0,29%
10	NTP 2018^ (1) [32]	13	180	16	180	0,813	0,402	1,643	9,07%
11	NTP 2018^ (2) [32]	16	180	16	180	1,000	0,515	1,942	10,21%
12	NTP 2018^ (3) [32]	19	180	16	180	1,188	0,630	2,239	11,18%
13	NTP 2018^ (4) [32]	16	179	16	180	1,006	0,518	1,953	10,22%
14	NTP 2018^ (5) [32]	14	180	16	180	0,875	0,439	1,743	9,47%
15	NTP 2018^ (6) [32]	16	180	16	180	1,000	0,515	1,942	10,21%
16	Sommer 2004 [72]	16	160	16	160	1,000	0,517	1,935	10,34%
17	Tillmann 2007 (1) [84]	5	100	10	100	0,500	0,176	1,420	4,15%
18	Tillmann 2007 (2) [84]	9	100	10	100	0,900	0,380	2,131	6,09%
19	Tillmann 2007 (3) [84]	5	100	10	100	0,500	0,176	1,420	4,15%
20	Tillmann 2007 (4) [84]	5	100	7	100	0,714	0,233	2,190	3,60%
21	Tillmann 2007 (5) [84]	4	100	7	100	0,571	0,171	1,905	3,12%
22	Tillmann 2007 (6) [84]	4	100	7	100	0,571	0,171	1,905	3,12%
23	Tillmann 2010 [73]	1	56	0	54	2,895	0,116	72,097	0,44%
	**Combined Effect Size**					**0,886**	**0,769**	**1,021**	
	**Heterogeneity**	**Tau = 0**	**I^2= 0**						
	**Overall effect**	**z = -1.771**	**(p = 0.077)**						

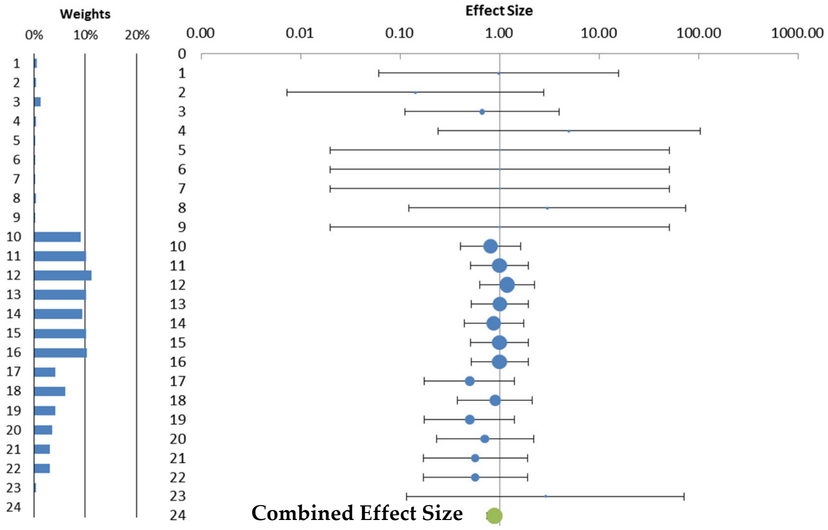

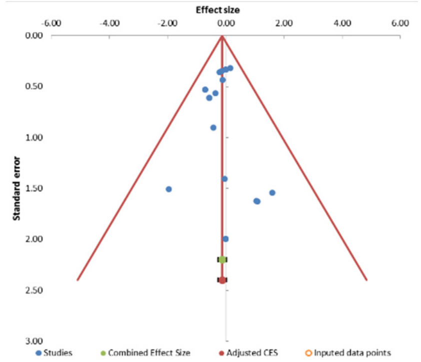

NTP 2018 [31] indicates NTP rat study, NTP 2018^ [32] indicates NTP mice study

Figure S2.16 Lymphoma

ID Study	study name	exposed events	exposed total number	sham events	sham total number	RR	lower limit	upper limit	weight %
1	Anderson 2004 (1) [75]	0	180	1	180	0,333	0,014	8,217	0,01%
2	Anderson 2004 (2) [75]	0	180	1	180	0,333	0,014	8,217	0,01%
3	Chou 1992 [63]	2	100	2	100	1,000	0,142	7,044	0,04%
4	Frei et al 1998a [65]	6	99	2	97	2,939	0,602	14,349	0,06%
5	La Regina 2003 (1) [76]	2	160	2	160	1,000	0,142	7,065	0,04%
6	La Regina 2003 (2) [76]	0	160	2	160	0,200	0,010	4,181	0,02%
7	Lee 2011 [69]	62	80	62	80	1,000	0,845	1,183	5,06%
8	NTP 2018 (1) [31]	8	180	7	180	1,143	0,422	3,096	0,14%
9	NTP 2018 (2) [31]	6	180	7	180	0,857	0,293	2,510	0,12%
10	NTP 2018 (3) [31]	7	180	7	180	1,000	0,357	2,803	0,13%
11	NTP 2018 (4) [31]	4	180	7	180	0,571	0,170	1,926	0,10%
12	NTP 2018 (5) [31]	5	180	7	180	0,714	0,230	2,217	0,11%
13	NTP 2018 (6) [31]	7	180	7	180	1,000	0,357	2,803	0,13%
14	NTP 2018^ (1) [32]	17	179	8	180	2,137	0,944	4,838	0,21%
15	NTP 2018^ (2) [32]	12	180	8	180	1,500	0,626	3,592	0,19%
16	NTP 2018^ (3) [32]	10	180	8	180	1,250	0,503	3,104	0,17%
17	NTP 2018^ (4) [32]	12	179	8	180	1,508	0,630	3,612	0,19%
18	NTP 2018^ (5) [32]	11	180	8	180	1,375	0,565	3,348	0,18%
19	NTP 2018^ (6) [32]	11	180	8	180	1,375	0,565	3,348	0,18%
20	Oberto 2007 (1) [81]	28	100	31	100	0,903	0,586	1,391	0,77%
21	Oberto 2007 (2) [81]	40	100	31	100	1,290	0,882	1,888	0,99%
22	Oberto 2007 (3) [81]	23	100	31	100	0,742	0,466	1,182	0,66%
23	Rapacholi 1997 [70]	43	94	22	93	1,934	1,259	2,970	0,78%
24	Sommer 2004 [72]	144	160	144	160	1,000	0,929	1,076	26,46%
25	Sommer 2007 [83]	141	160	149	160	0,946	0,881	1,016	28,20%
26	Tillmann 2007 (1) [84]	22	100	23	100	0,957	0,570	1,605	0,53%
27	Tillmann 2007 (2) [84]	25	100	23	100	1,087	0,661	1,786	0,58%
28	Tillmann 2007 (3) [84]	24	100	23	100	1,043	0,631	1,726	0,56%
29	Tillmann 2007 (4) [84]	22	100	23	100	0,957	0,570	1,605	0,53%
30	Tillmann 2007 (5) [84]	22	100	23	100	0,957	0,570	1,605	0,53%
31	Tillmann 2007 (6) [84]	21	100	23	100	0,913	0,540	1,545	0,52%
32	Tillmann 2010 [73]	16	56	17	54	0,908	0,509	1,617	0,43%
33	Toler 1997 [74]	13	200	13	200	1,000	0,474	2,108	0,26%
34	Utteridge 2002 (1) [85]	40	120	38	120	1,053	0,729	1,519	1,06%
35	Utteridge 2002 (2) [85]	37	120	38	120	0,974	0,668	1,420	1,00%
36	Utteridge 2002 (3) [85]	45	120	38	120	1,184	0,833	1,683	1,15%
37	Utteridge 2002 (4) [85]	36	118	38	120	0,963	0,659	1,409	0,99%
38	Utteridge 2002 (5) [85]	88	120	89	120	0,989	0,850	1,151	6,20%
39	Utteridge 2002 (6) [85]	86	120	89	120	0,966	0,827	1,128	5,93%
40	Utteridge 2002 (7) [85]	93	120	89	120	1,045	0,905	1,206	6,91%
41	Utteridge 2002 (8) [85]	99	120	89	120	1,112	0,972	1,273	7,87%
	**Combined Effect Size**					**1,003**	**0,969**	**1,038**	
	**Heterogeneity**	**Tau = 0**	**$I^2 = 0$**						
	**Overall effect**	**z = 0.167**	**(p = 0.868)**						

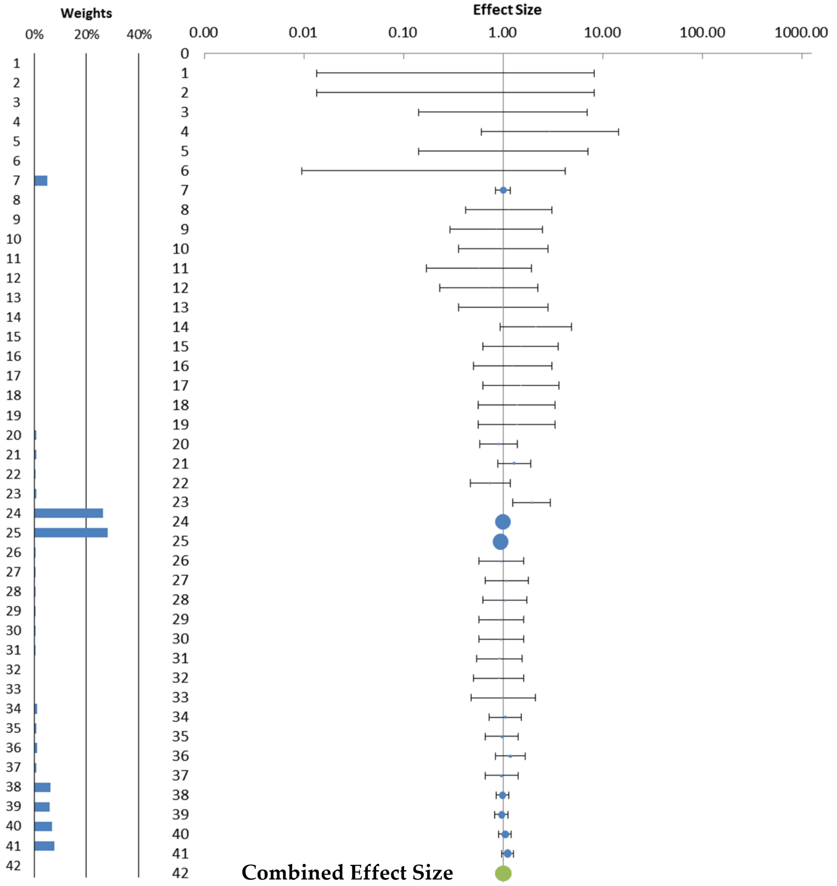

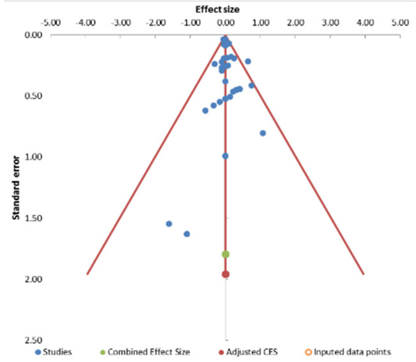

NTP 2018 [31] indicates NTP rat study, NTP 2018^ [32] indicates NTP mice study

Figure S2.17 Mammary Malignant

ID Study	study name	exposed events	exposed total number	sham events	sham total number	RR	lower limit	upper limit	weight %
1	Anderson 2004 (1) [75]	1	180	1	180	1,000	0,062	16,014	0,67%
2	Anderson 2004 (2) [75]	2	180	1	180	2,000	0,181	22,039	0,89%
3	Bartsch 2010 (exp3) [82]	9	29	12	28	0,724	0,357	1,468	6,08%
4	Bartsch 2010 (exp4) [82]	7	29	10	30	0,724	0,313	1,674	4,99%
5	Frei et al 1998 b [66]	38	98	30	99	1,280	0,865	1,892	9,57%
6	Frei et al 1998a [65]	44	95	55	88	0,741	0,565	0,973	11,10%
7	La Regina 2003 (1) [76]	1	160	2	160	0,500	0,045	5,509	0,89%
8	La Regina 2003 (2) [76]	0	160	2	160	0,200	0,010	4,181	0,57%
9	NTP 2018 (1) [31]	6	165	10	172	0,625	0,232	1,688	3,89%
10	NTP 2018 (2) [31]	9	171	10	172	0,905	0,376	2,179	4,59%
11	NTP 2018 (3) [31]	6	172	10	172	0,600	0,222	1,620	3,89%
12	NTP 2018 (4) [31]	8	167	10	172	0,824	0,332	2,043	4,40%
13	NTP 2018 (5) [31]	6	170	10	172	0,607	0,225	1,639	3,89%
14	NTP 2018 (6) [31]	4	170	10	172	0,405	0,129	1,271	3,17%
15	NTP 2018^ (1) [32]	0	93	0	87	0,936	0,018	47,939	0,35%
16	NTP 2018^ (2) [32]	1	90	0	87	2,901	0,117	71,841	0,51%
17	NTP 2018^ (3) [32]	1	92	0	87	2,839	0,115	70,288	0,51%
18	NTP 2018^ (4) [32]	0	88	0	87	0,989	0,019	50,657	0,35%
19	NTP 2018^ (5) [32]	0	90	0	87	0,967	0,019	49,534	0,35%
20	NTP 2018^ (6) [32]	2	91	0	87	4,783	0,228	100,301	0,57%
21	Smith 2007 (1) [77]	4	100	2	100	2,000	0,371	10,784	1,69%
22	Smith 2007 (2) [77]	1	100	2	100	0,500	0,045	5,506	0,89%
23	Smith 2007 (3) [77]	2	100	2	100	1,000	0,142	7,044	1,30%
24	Smith 2007 (4) [77]	4	100	3	100	1,333	0,303	5,858	2,10%
25	Smith 2007 (5) [77]	2	100	3	100	0,667	0,113	3,947	1,53%
26	Smith 2007 (6) [77]	3	99	3	100	1,010	0,207	4,932	1,87%
27	Szmigielski 1982 (1) [78]	32	40	14	40	2,286	1,447	3,610	8,78%
28	Szmigielski 1982 (2) [78]	37	40	14	40	2,643	1,705	4,096	9,02%
29	Toler 1997 [74]	85	193	82	190	1,020	0,812	1,283	11,60%
	**Combined Effect Size**					**1,034**	**0,839**	**1,275**	
	**Heterogeneity**	**Tau = 0.329**	**I^2= 43.71 %**						
	**Overall effect**	**z = 0.329**	**(p = 0.742)**						

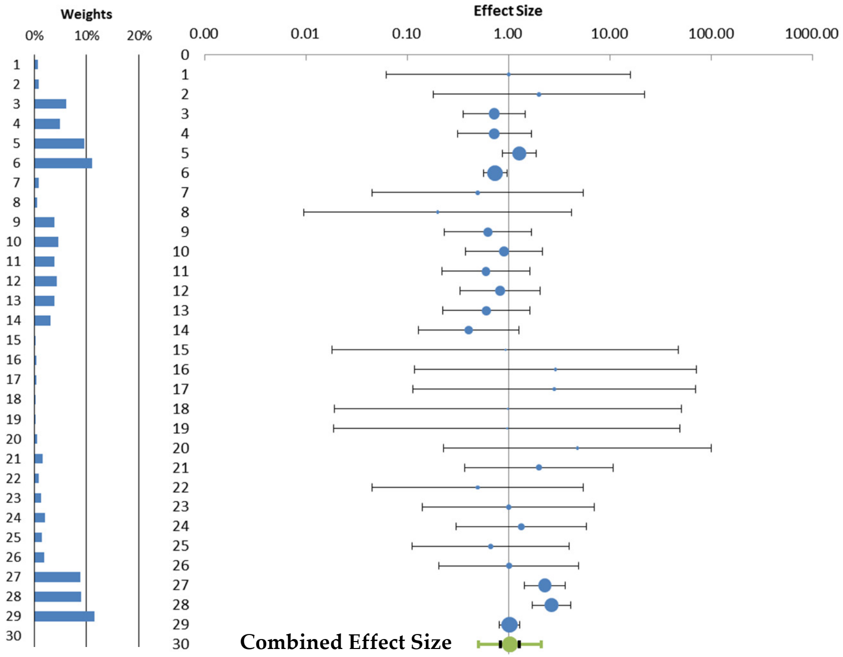

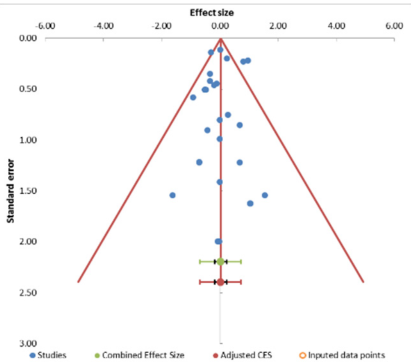

NTP 2018 [31] indicates NTP rat study, NTP 2018^ [32] indicates NTP mice study

Figure S2.18 Mesentheric Lympho-node Malignant

		exposed		sham					
ID Study	study name	events	total number	events	total number	RR	lower limit	upper limit	weight %
1	Frei et al 1998 b [66]	3	95	1	98	3,095	0,323	29,652	11,71%
2	NTP 2018 (1) [31]	0	180	0	180	1,000	0,020	50,795	3,85%
3	NTP 2018 (2) [31]	0	180	0	180	1,000	0,020	50,795	3,85%
4	NTP 2018 (3) [31]	2	180	0	180	5,000	0,239	104,489	6,44%
5	NTP 2018^ (1) [32]	0	172	1	156	0,303	0,012	7,460	5,79%
6	NTP 2018^ (2) [32]	0	166	1	156	0,313	0,013	7,729	5,79%
7	NTP 2018^ (3) [32]	1	168	1	156	0,929	0,058	14,873	7,73%
8	NTP 2018^ (4) [32]	1	174	1	156	0,897	0,056	14,359	7,73%
9	NTP 2018^ (5) [32]	0	161	1	156	0,323	0,013	7,968	5,79%
10	NTP 2018^ (6) [32]	0	166	1	156	0,313	0,013	7,729	5,79%
11	Smith 2007 (1) [83]	0	99	0	98	0,990	0,019	50,624	3,86%
12	Smith 2007 (2) [83]	1	100	0	98	2,941	0,119	72,748	5,81%
13	Smith 2007 (3) [83]	2	100	0	98	4,901	0,234	102,710	6,46%
14	Smith 2007 (4) [83]	0	100	2	99	0,198	0,009	4,150	6,46%
15	Smith 2007 (5) [83]	0	100	2	99	0,198	0,009	4,150	6,46%
16	Smith 2007 (6) [83]	0	100	2	99	0,198	0,009	4,150	6,46%
	**Combined Effect Size**					**0,822**	**0,436**	**1,550**	
	**Heterogeneity**	**Tau = 0**	**I^2 = 0**						
	**Overall effect**	**z = -0.659**	**(p = 0.510)**						

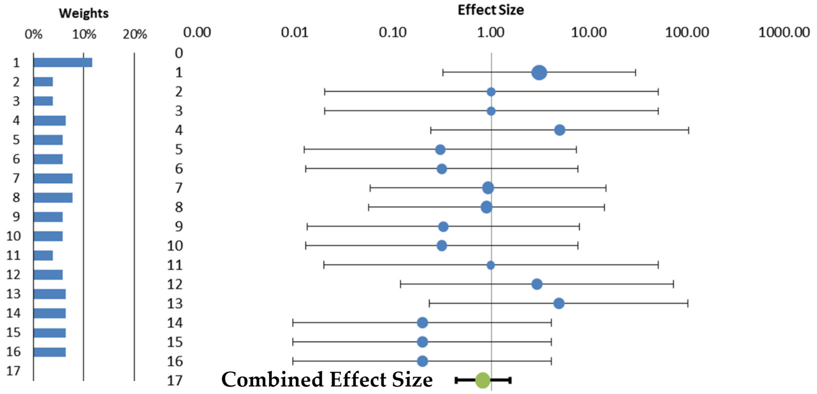

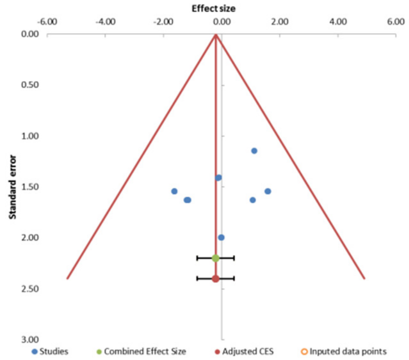

NTP 2018 [31] indicates NTP rat study, NTP 2018^ [32] indicates NTP mice study

Figure S2.19 Pancreas Malignant

ID Study	study name	exposed events	exposed total number	sham events	sham total number	RR	lower limit	upper limit	weight %
1	NTP 2018 (1) [31]	17	178	11	180	1,563	0,752	3,250	17,07%
2	NTP 2018 (2) [31]	18	178	11	180	1,655	0,803	3,411	17,48%
3	NTP 2018 (3) [31]	9	173	11	180	0,851	0,361	2,009	12,41%
4	NTP 2018 (4) [31]	9	178	11	180	0,827	0,350	1,954	12,39%
5	NTP 2018 (5) [31]	15	177	11	180	1,387	0,653	2,943	16,16%
6	NTP 2018 (6) [31]	8	167	11	180	0,784	0,322	1,907	11,57%
7	NTP 2018^ (1) [32]	1	176	1	175	0,994	0,062	15,924	1,19%
8	NTP 2018^ (2) [32]	2	179	1	175	1,955	0,177	21,546	1,59%
9	NTP 2018^ (3) [32]	2	175	1	175	2,000	0,182	22,038	1,59%
10	NTP 2018^ (4) [32]	1	178	1	175	0,983	0,061	15,745	1,19%
11	NTP 2018^ (5) [32]	0	178	1	175	0,328	0,013	8,080	0,89%
12	NTP 2018^ (6) [32]	0	176	1	175	0,331	0,013	8,171	0,89%
13	Smith 2007 (1) [83]	0	100	1	100	0,333	0,013	8,246	0,89%
14	Smith 2007 (2) [83]	0	100	1	100	0,333	0,013	8,246	0,89%
15	Smith 2007 (3) [83]	2	100	1	100	2,000	0,182	22,026	1,60%
16	Smith 2007 (4) [83]	0	100	0	100	1,000	0,020	51,124	0,59%
17	Smith 2007 (5) [83]	2	100	0	100	5,000	0,239	104,778	0,99%
18	Smith 2007 (6) [83]	0	100	0	100	5,000	0,239	104,778	0,59%
	**Combined Effect Size**					**1,167**	**0,940**	**1,448**	
	**Heterogeneity**	**Tau = 0**	**I^2 = 0**						
	**Overall effect**	**z = 1.510**	**(p = 0.131)**						

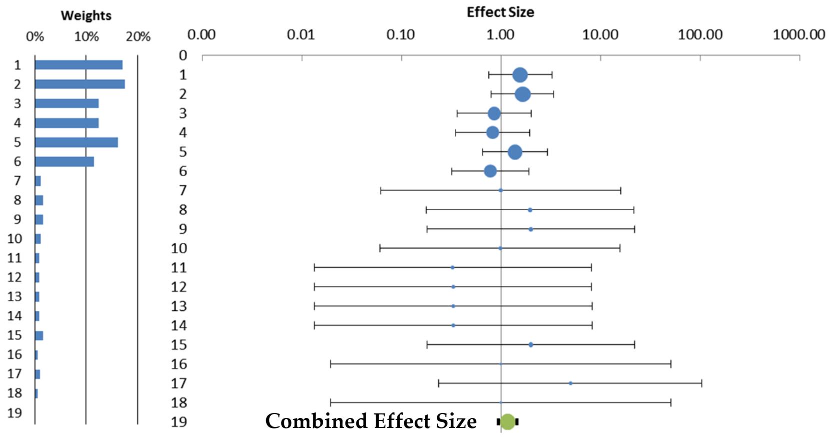

NTP 2018 [31] indicates NTP rat study, NTP 2018^ [32] indicates NTP mice study

Figure S2.20 Pituitary Malignant

		exposed		sham					
ID Study	study name	events	total number	events	total number	RR	lower limit	upper limit	weight %
1	Bartsch 2010 (exp1) [82]	5	12	9	12	0,556	0,253	1,222	21,63%
2	Bartsch 2010 (exp2) [82]	4	12	6	12	0,667	0,236	1,880	12,50%
3	Bartsch 2010 (exp3) [82]	8	29	6	28	1,287	0,501	3,306	14,11%
4	Bartsch 2010 (exp4) [82]	11	29	11	30	1,034	0,526	2,034	27,41%
5	Chou 1992 [63]	2	100	0	100	5,000	0,239	104,778	1,31%
6	La Regina 2003 (1) [76]	0	160	0	160	1,000	0,020	50,845	0,78%
7	La Regina 2003 (2) [76]	1	160	0	160	3,000	0,122	73,992	1,18%
8	NTP 2018 (1) [31]	1	180	1	179	0,994	0,062	15,925	1,57%
9	NTP 2018 (2) [31]	0	180	1	179	0,331	0,013	8,172	1,18%
10	NTP 2018 (3) [31]	1	180	1	179	0,994	0,062	15,925	1,57%
11	NTP 2018 (4) [31]	1	179	1	179	1,000	0,062	16,014	1,57%
12	NTP 2018 (5) [31]	1	179	1	179	1,000	0,062	16,014	1,57%
13	NTP 2018 (6) [31]	0	180	1	179	0,331	0,013	8,172	1,18%
14	NTP 2018^ (1) [32]	0	165	0	166	1,006	0,020	51,136	0,78%
15	NTP 2018^ (2) [32]	2	171	0	166	4,855	0,232	101,475	1,31%
16	NTP 2018^ (3) [32]	1	169	0	166	2,947	0,120	72,670	1,18%
17	NTP 2018^ (4) [32]	0	163	0	166	1,018	0,020	51,763	0,78%
18	NTP 2018^ (5) [32]	1	177	0	166	2,815	0,114	69,396	1,18%
19	NTP 2018^ (6) [32]	1	169	0	166	2,947	0,120	72,670	1,18%
20	Smith 2007 (4) [83]	0	100	0	100	1,000	0,020	51,124	0,79%
21	Smith 2007 (5) [83]	1	100	0	100	3,000	0,121	74,212	1,18%
22	Smith 2007 (6) [83]	1	100	0	100	3,000	0,121	74,212	1,18%
23	Tillmann 2007 (1) [84]	0	100	0	100	1,000	0,020	51,124	0,79%
24	Tillmann 2007 (2) [84]	0	99	0	100	1,010	0,020	51,639	0,79%
25	Tillmann 2007 (3) [84]	2	100	0	100	5,000	0,239	104,778	1,31%
	**Combined Effect Size**					**0,981**	**0,774**	**1,243**	
	**Heterogeneity**	**Tau = 0**	**I^2= 0**						
	**Overall effect**	**z = -0.169**	**(p = 0.866)**						

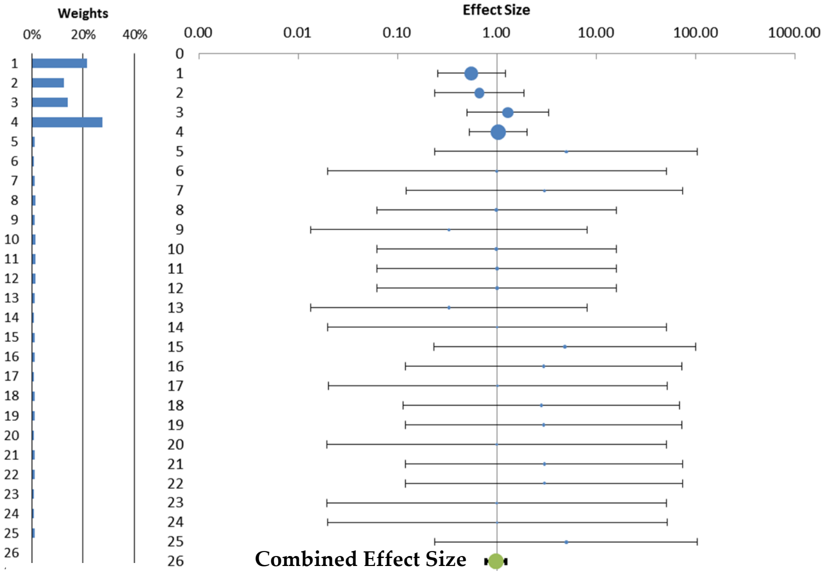

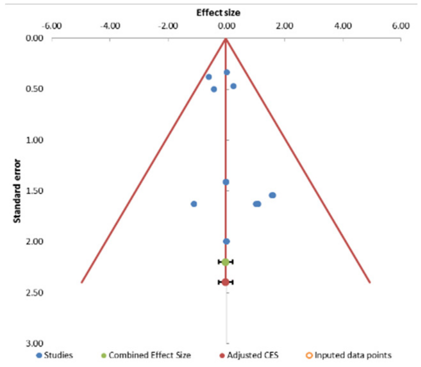

NTP 2018 [31] indicates NTP rat study, NTP 2018^ [32] indicates NTP mice study

Figure S2.21 Skin Malignant

		exposed		sham					
ID Study	study name	events	total number	events	total number	RR	lower limit	upper limit	weight %
1	Chou 1992 [63]	2	100	0	100	5,000	0,239	104,778	0,94%
2	Frei et al 1998 b [66]	4	100	7	100	0,571	0,171	1,905	6,02%
3	Frei et al 1998a [65]	3	98	4	98	0,750	0,171	3,294	3,99%
4	NTP 2018 (1) [31]	6	180	6	180	1,000	0,327	3,054	6,97%
5	NTP 2018 (2) [31]	7	180	6	180	1,167	0,398	3,416	7,52%
6	NTP 2018 (3) [31]	5	180	6	180	0,833	0,258	2,692	6,31%
7	NTP 2018 (4) [31]	6	180	6	180	1,000	0,327	3,054	6,97%
8	NTP 2018 (5) [31]	6	180	6	180	1,000	0,327	3,054	6,97%
9	NTP 2018 (6) [31]	2	180	6	180	0,333	0,068	1,638	3,42%
10	NTP 2018^ (1) [32]	3	178	10	180	0,303	0,085	1,089	5,32%
11	NTP 2018^ (2) [32]	14	180	10	180	1,400	0,637	3,077	14,00%
12	NTP 2018^ (3) [32]	6	180	10	180	0,600	0,222	1,621	8,78%
13	NTP 2018^ (4) [32]	3	180	10	180	0,300	0,084	1,077	5,32%
14	NTP 2018^ (5) [32]	7	180	10	180	0,700	0,272	1,804	9,69%
15	NTP 2018^ (6) [32]	5	180	10	180	0,500	0,174	1,439	7,77%
	**Combined Effect Size**					**0,755**	**0,566**	**1,008**	
	**Heterogeneity**	**Tau = 0**	**I^2 = 0**						
	**Overall effect**	**z = -2.088**	**(p = 0.037)**						

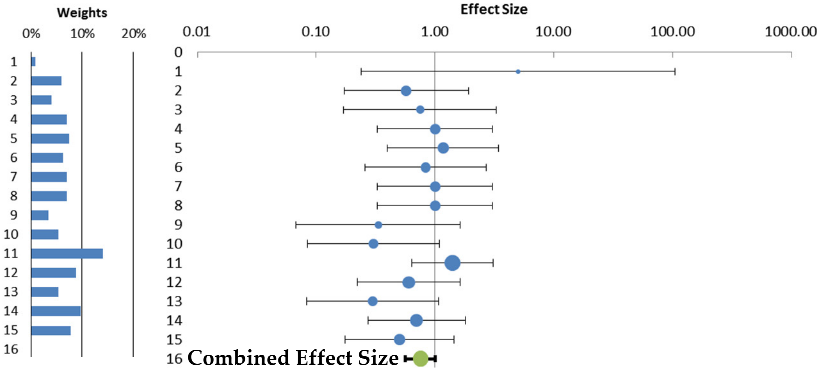

NTP 2018 [31] indicates NTP rat study, NTP 2018^ [32] indicates NTP mice study

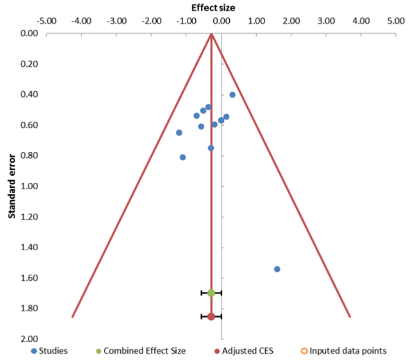

Figure S2.22 Spleen Malignant

ID Study	study name	exposed events	exposed total number	sham events	sham total number	RR	lower limit	upper limit	weight %
1	Frei et al 1998 b [66]	3	101	1	99	2,941	0,307	28,178	8,83%
2	NTP 2018 (1) [31]	0	180	4	180	0,111	0,006	2,069	6,71%
3	NTP 2018 (2) [31]	0	179	4	180	0,112	0,006	2,081	6,71%
4	NTP 2018 (3) [31]	2	180	4	180	0,500	0,092	2,711	11,08%
5	NTP 2018 (4) [31]	0	180	4	180	0,111	0,006	2,069	6,71%
6	NTP 2018 (5) [31]	0	180	4	180	0,111	0,006	2,069	6,71%
7	NTP 2018 (6) [31]	0	175	4	180	0,114	0,006	2,128	6,71%
8	NTP 2018^ (1) [32]	6	175	0	173	12,852	0,722	228,695	6,83%
9	NTP 2018^ (2) [32]	3	178	0	173	6,804	0,350	132,118	6,59%
10	NTP 2018^ (3) [32]	1	175	0	173	2,966	0,120	73,122	5,99%
11	NTP 2018^ (4) [32]	5	176	0	173	10,814	0,596	196,055	6,78%
12	NTP 2018^ (5) [32]	3	173	0	173	7,000	0,361	135,921	6,59%
13	NTP 2018^ (6) [32]	3	174	0	173	6,960	0,358	135,143	6,59%
14	Tillmann 2010 [73]	1	56	1	54	0,964	0,060	15,506	7,18%
	**Combined Effect Size**					**1,067**	**0,353**	**3,222**	
	**Heterogeneity**	**Tau = 1.232**	**I^2= 44.49%**						
	**Overall effect**	**z = 0.127**	**(p = 0.899)**						

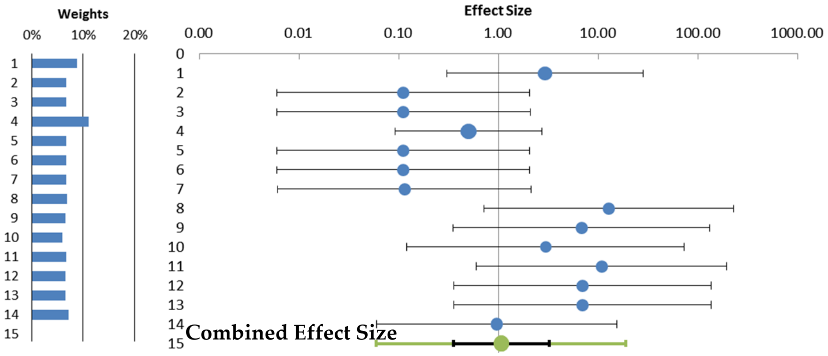

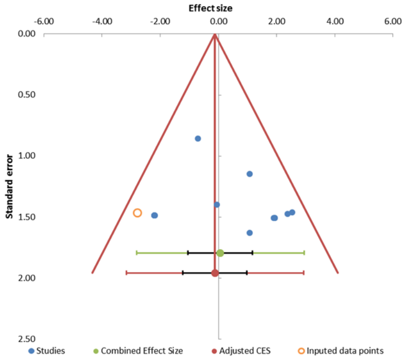

NTP 2018 [31] indicates NTP rat study, NTP 2018^ [32] indicates NTP mice study

Figure S2.23 Stomach Malignant

		exposed		sham					
ID Study	study name	events	total number	events	total number	RR	lower limit	upper limit	weight %
1	Chou 1992 [63]	1	100	0	100	3,000	0,121	74,212	12,35%
2	NTP 2018 (1) [31]	0	180	1	180	0,333	0,014	8,217	12,31%
3	NTP 2018 (2) [31]	0	180	1	180	0,333	0,014	8,217	12,31%
4	NTP 2018 (3) [31]	2	180	1	180	2,000	0,181	22,039	21,96%
5	NTP 2018 (4) [31]	0	180	1	180	0,333	0,014	8,217	12,31%
6	NTP 2018 (5) [31]	0	179	1	180	0,335	0,014	8,263	12,31%
7	NTP 2018 (6) [31]	1	180	1	180	1,000	0,062	16,014	16,44%
	Combined Effect Size					0,777	0,321	1,881	
	Heterogeneity	Tau = 0	$I^2 = 0$						
	Overall effect	z = -0.698	(p = 0.485)						

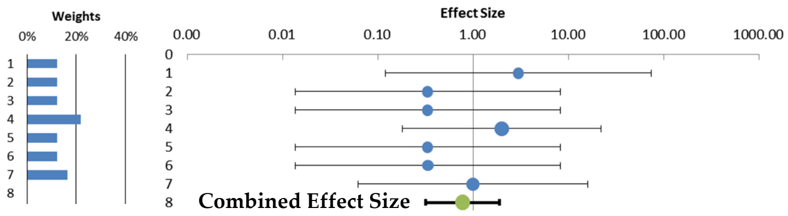

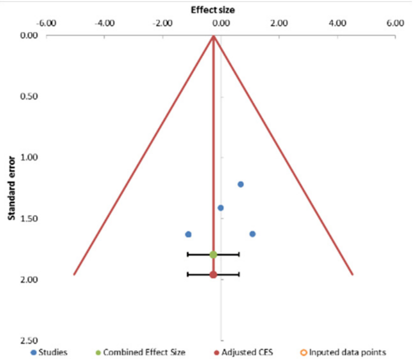

NTP 2018 [31] indicates NTP rat study

Figure S2.24 Thymus Malignant

ID Study	study name	exposed events	exposed total number	sham events	sham total number	RR	lower limit	upper limit	weight %
1	La Regina 2003 (1) [76]	1	160	0	160	3.000	0.122	73.992	5.75%
2	La Regina 2003 (2) [76]	0	160	0	160	1.000	0.020	50.845	3.83%
3	NTP 2018 (1) [31]	1	172	1	175	1.017	0.064	16.295	7.67%
4	NTP 2018 (2) [31]	1	176	1	175	0.994	0.062	15.924	7.67%
5	NTP 2018 (3) [31]	1	172	1	175	1.017	0.064	16.295	7.67%
6	NTP 2018 (4) [31]	0	168	1	175	0.347	0.014	8.559	5.75%
7	NTP 2018 (5) [31]	1	174	1	175	1.006	0.063	16.107	7.67%
8	NTP 2018 (6) [31]	0	169	1	175	0.345	0.014	8.509	5.75%
9	Smith 2007 (1) [83]	1	97	0	98	3.031	0.122	74.985	5.77%
10	Smith 2007 (2) [83]	1	99	0	98	2.970	0.120	73.479	5.76%
11	Smith 2007 (3) [83]	1	99	0	98	2.970	0.120	73.479	5.76%
12	Smith 2007 (4) [83]	1	100	2	94	0.470	0.043	5.176	10.31%
13	Smith 2007 (5) [83]	1	97	2	94	0.485	0.044	5.336	10.32%
14	Smith 2007 (6) [83]	1	95	2	94	0.495	0.045	5.448	10.32%
	**Combined Effect Size**					**0.912**	**0.582**	**1.429**	
	**Heterogeneity**	**Tau = 0**	**$I^2 = 0$**						
	**Overall effect**	**z = -0.442**	**(p = 0.658)**						

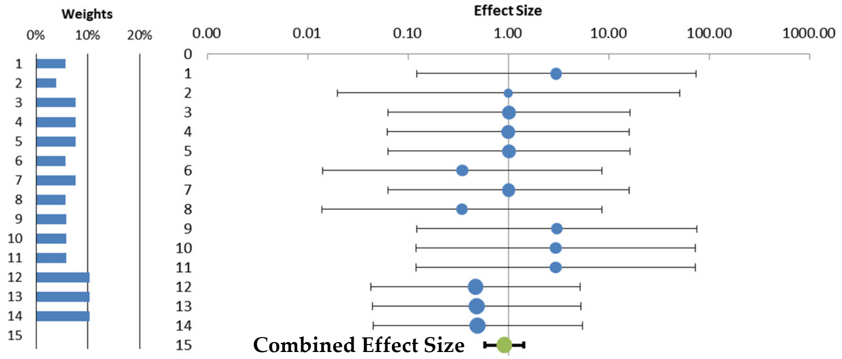

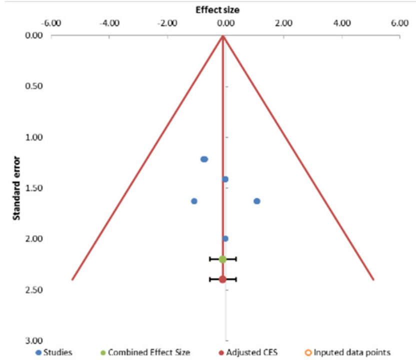

NTP 2018 [31] indicates NTP rat study

Figure S2.25 Thyroid Malignant

ID Study	study name	exposed events	exposed total number	sham events	sham total number	RR	lower limit	upper limit	weight %
1	Anderson 2004 (1) [75]	16	180	11	180	1.455	0.693	3.054	20.73%
2	Anderson 2004 (2) [75]	17	180	11	180	1.545	0.743	3.214	21.28%
3	Chou 1992 [63]	2	100	0	100	5.000	0.239	104.778	1.24%
4	La Regina 2003 (1) [76]	2	160	5	160	0.400	0.078	2.044	4.29%
5	La Regina 2003 (2) [76]	2	160	5	160	0.400	0.078	2.044	4.29%
6	NTP 2018 (1) [31]	2	177	3	179	0.674	0.113	4.011	3.59%
7	NTP 2018 (2) [31]	3	179	3	179	1.000	0.203	4.915	4.50%
8	NTP 2018 (3) [31]	5	175	3	179	1.705	0.412	7.060	5.65%
9	NTP 2018 (4) [31]	4	177	3	179	1.348	0.305	5.968	5.16%
10	NTP 2018 (5) [31]	5	176	3	179	1.695	0.409	7.020	5.65%
11	NTP 2018 (6) [31]	6	174	3	179	2.057	0.520	8.136	6.04%
12	NTP 2018^ (1) [32]	0	177	1	175	0.330	0.013	8.125	1.11%
13	NTP 2018^ (2) [32]	1	174	1	175	1.006	0.063	16.107	1.48%
14	NTP 2018^ (3) [32]	0	174	1	175	0.335	0.014	8.265	1.11%
15	NTP 2018^ (4) [32]	0	176	1	175	0.331	0.013	8.171	1.11%
16	NTP 2018^ (5) [32]	0	176	1	175	0.331	0.013	8.171	1.11%
17	NTP 2018^ (6) [32]	0	175	1	175	0.333	0.014	8.218	1.11%
18	Oberto 2007 (1) [81]	0	100	0	100	1.000	0.020	51.124	0.74%
19	Oberto 2007 (2) [81]	1	100	0	100	1.000	0.020	51.124	0.74%
20	Oberto 2007 (3) [81]	0	100	0	100	3.000	0.121	74.212	1.11%
21	Smith 2007 (1) [83]	1	100	0	100	3.000	0.121	74.212	1.11%
22	Smith 2007 (2) [83]	1	100	0	100	3.000	0.121	74.212	1.11%
23	Smith 2007 (3) [83]	1	100	0	100	3.000	0.121	74.212	1.11%
24	Smith 2007 (4) [83]	0	100	1	100	0.333	0.013	8.246	1.11%
25	Smith 2007 (5) [83]	2	100	1	100	2.000	0.182	22.026	1.99%
26	Smith 2007 (6) [83]	1	100	1	100	1.000	0.062	16.037	1.49%
	**Combined Effect Size**					**1.229**	**0.963**	**1.567**	
	**Heterogeneity**	**Tau = 0**	**I^2 = 0**						
	**Overall effect**	**z = 1.742**	**(p = 0.081)**						

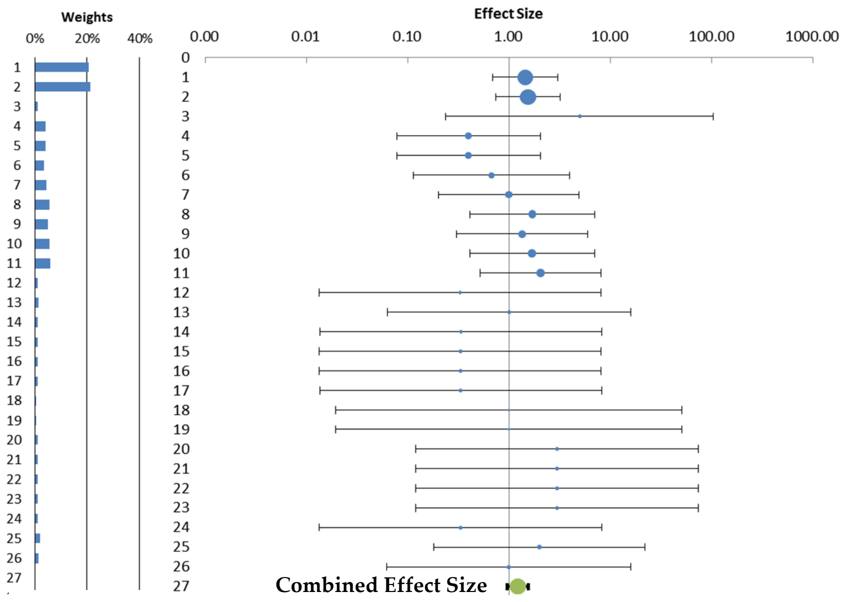

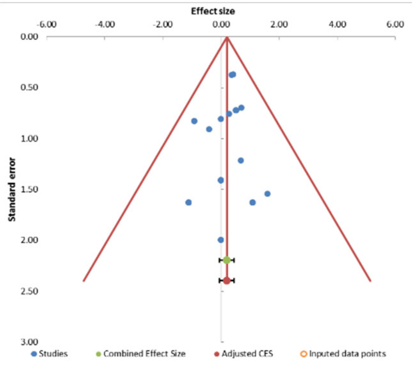

NTP 2018 [31] indicates NTP rat study, NTP 2018^ [32] indicates NTP mice study

Figure S2.26 Adrenals Benign

ID Study	study name	exposed events	exposed total number	sham events	sham total number	RR	lower limit	upper limit	weight %
1	Chou 1992 [63]	17	100	12	100	1.417	0.711	2.822	7.61%
2	Frei et al 1998 b [66]	1	99	4	100	0.253	0.028	2.250	1.31%
3	Frei et al 1998a [65]	2	95	2	93	0.979	0.139	6.892	1.61%
4	La Regina 2003 (1) [76]	6	160	12	160	0.500	0.192	1.304	5.07%
5	La Regina 2003 (2) [76]	12	160	12	160	1.000	0.462	2.165	6.68%
6	NTP 2018 (1) [31]	35	180	13	180	2.692	1.471	4.926	8.66%
7	NTP 2018 (2) [31]	31	179	13	180	2.398	1.295	4.439	8.50%
8	NTP 2018 (3) [31]	22	178	13	180	1.711	0.888	3.297	7.99%
9	NTP 2018 (4) [31]	31	180	13	180	2.385	1.288	4.415	8.50%
10	NTP 2018 (5) [31]	27	180	13	180	2.077	1.105	3.902	8.31%
11	NTP 2018 (6) [31]	18	180	13	180	1.385	0.698	2.747	7.64%
12	NTP 2018^ (1) [32]	3	177	1	174	2.949	0.307	28.299	1.23%
13	NTP 2018^ (2) [32]	4	179	1	174	3.888	0.436	34.704	1.30%
14	NTP 2018^ (3) [32]	0	178	1	174	0.326	0.013	8.034	0.63%
15	NTP 2018^ (4) [32]	2	177	1	174	1.966	0.178	21.665	1.10%
16	NTP 2018^ (5) [32]	5	177	1	174	4.915	0.576	41.955	1.35%
17	NTP 2018^ (6) [32]	6	177	1	174	5.898	0.712	48.844	1.39%
18	Oberto 2007 (1) [81]	0	100	2	100	0.200	0.010	4.191	0.70%
19	Oberto 2007 (2) [81]	3	100	2	100	1.500	0.253	8.881	1.90%
20	Oberto 2007 (3) [81]	1	100	2	100	0.500	0.045	5.506	1.10%
21	Tillmann 2007 (1) [84]	4	100	7	100	0.571	0.171	1.905	3.64%
22	Tillmann 2007 (2) [84]	7	100	7	100	1.000	0.362	2.764	4.69%
23	Tillmann 2007 (3) [84]	2	100	7	100	0.286	0.060	1.355	2.40%
24	Tillmann 2007 (4) [84]	5	100	3	100	1.667	0.406	6.846	2.82%
25	Tillmann 2007 (5) [84]	4	100	3	100	1.333	0.303	5.858	2.61%
26	Tillmann 2007 (6) [84]	1	100	3	100	0.333	0.035	3.194	1.23%
	**Combined Effect Size**					**1.433**	**1.088**	**1.888**	
	**Heterogeneity**	**Tau = 0.329**	**I^2= 28.08%**						
	**Overall effect**	**z = 2.686**	**(p = 0.007)**						

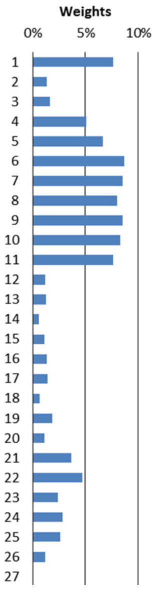

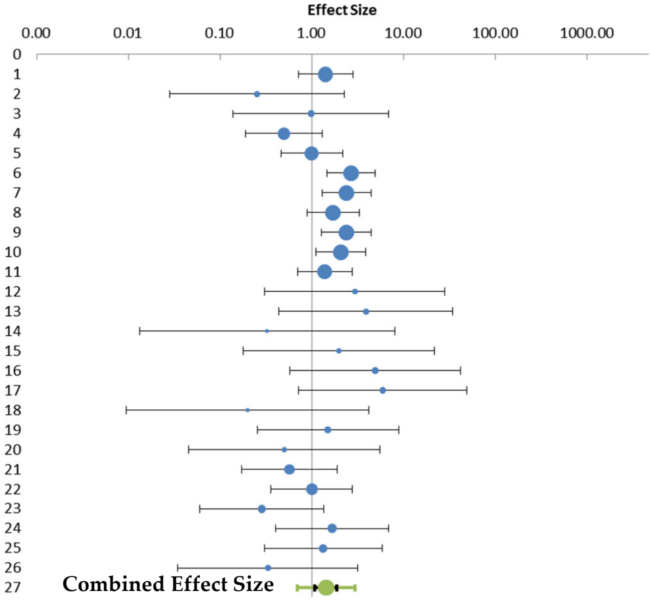

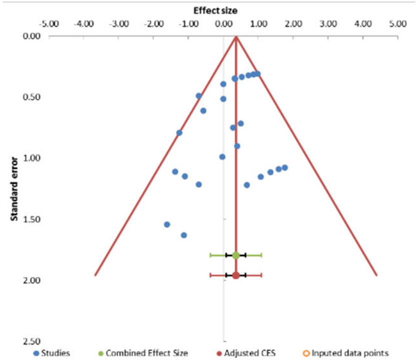

NTP 2018 [31] indicates NTP rat study, NTP 2018^ [32] indicates NTP mice study

Figure S2.27 CNS/Brain Benign

ID Study	study name	exposed events	exposed total number	sham events	sham total number	RR	lower limit	upper limit	weight %
1	Falcioni 2018 (1) [80]	8	811	2	817	4.030	0.857	18.940	14.33%
2	Falcioni 2018 (2) [80]	3	411	2	817	2.982	0.499	17.806	10.75%
3	Falcioni 2018 (3) [80]	4	409	2	817	3.995	0.734	21.758	11.95%
4	NTP 2018 (1) [31]	4	180	2	180	2.000	0.369	10.844	12.07%
5	NTP 2018 (2) [31]	5	180	2	180	2.500	0.489	12.789	12.95%
6	NTP 2018 (3) [31]	3	180	2	180	1.500	0.252	8.924	10.85%
7	NTP 2018 (4) [31]	1	180	2	180	0.500	0.045	5.510	5.99%
8	NTP 2018 (5) [31]	2	180	2	180	1.000	0.141	7.069	9.02%
9	NTP 2018 (6) [31]	4	180	2	180	2.000	0.369	10.844	12.07%
	**Combined Effect Size**					**2.163**	**1.371**	**3.411**	
	**Heterogeneity**	**Tau = 0**	**$I^2 = 0$**						
	**Overall effect**	**z = 3.902**	**(p<<0.01)**						

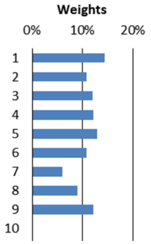

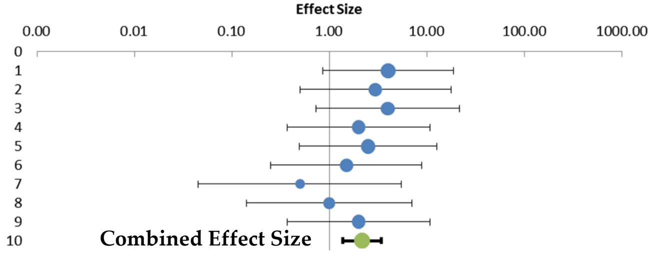

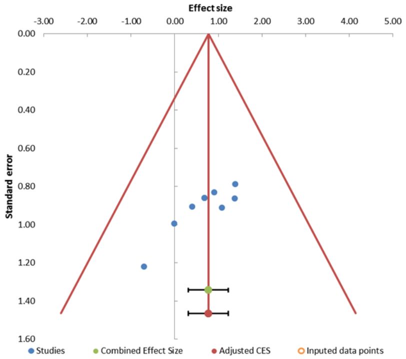

NTP 2018 [31] indicates NTP rat study

Figure S2.28 Sensorial System Benign

		exposed		sham					
ID Study	study name	events	total number	events	total number	RR	lower limit	upper limit	weight %
1	NTP 2018^ (1) [32]	14	179	10	179	1.400	0.637	3.076	9.13%
2	NTP 2018^ (2) [32]	16	180	10	179	1.591	0.740	3.420	9.57%
3	NTP 2018^ (3) [32]	11	180	10	179	1.094	0.475	2.518	8.30%
4	NTP 2018^ (4) [32]	12	179	10	179	1.200	0.531	2.714	8.61%
5	NTP 2018^ (5) [32]	12	179	10	179	1.200	0.531	2.714	8.61%
6	NTP 2018^ (6) [32]	8	179	10	179	0.800	0.322	1.986	7.17%
7	Oberto 2007 (1) [63]	2	100	0	100	5.000	0.239	104.778	0.74%
8	Oberto 2007 (2) [63]	2	100	0	100	5.000	0.239	104.778	0.74%
9	Oberto 2007 (3) [63]	4	100	0	100	9.000	0.482	167.974	0.80%
10	Tillmann 2007 (1) [84]	10	100	9	100	1.111	0.469	2.631	7.89%
11	Tillmann 2007 (2) [84]	9	100	9	100	1.000	0.412	2.427	7.52%
12	Tillmann 2007 (3) [84]	12	100	9	100	1.333	0.585	3.038	8.51%
13	Tillmann 2007 (4) [84]	5	100	14	100	0.357	0.133	0.960	6.23%
14	Tillmann 2007 (5) [84]	7	100	14	100	0.500	0.210	1.193	7.78%
15	Tillmann 2007 (6) [84]	8	100	14	100	0.571	0.250	1.308	8.43%
	**Combined Effect Size**					**1.007**	**0.754**	**1.345**	
	**Heterogeneity**	**Tau = 0.188**	**I^2 = 13.35%**						
	**Overall effect**	**z = 0.051**	**(p = 0.959)**						

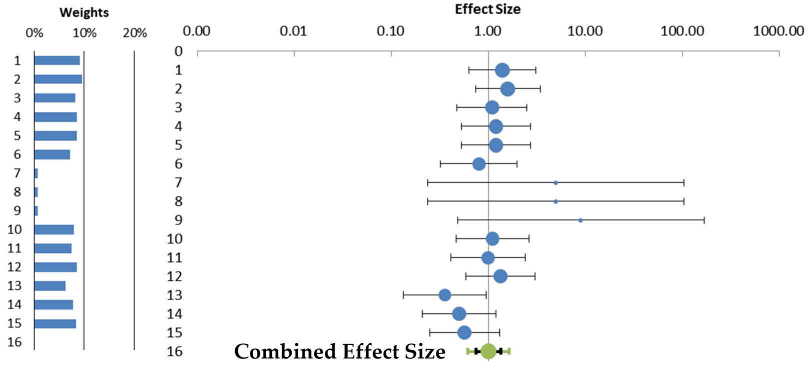

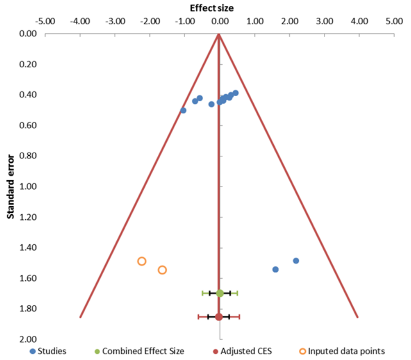

NTP 2018^ [32] indicates NTP mice study

Figure S2.29 Female Uro-Genital System Benign

ID Study	study name	exposed events	exposed total number	sham events	sham total number	RR	lower limit	upper limit	weight %
1	Frei et al 1998 b [66]	14	100	15	99	0.924	0.469	1.819	5.37%
2	Frei et al 1998a [65]	12	97	9	92	1.265	0.556	2.875	3.66%
3	La Regina 2003 (1) [76]	12	80	12	80	1.000	0.475	2.103	4.47%
4	La Regina 2003 (2) [76]	14	80	12	80	1.167	0.573	2.376	4.88%
5	NTP 2018 (1)F [31]	22	90	19	90	1.158	0.672	1.994	8.35%
6	NTP 2018 (2)F [31]	13	90	19	90	0.684	0.358	1.306	5.90%
7	NTP 2018 (3)F [31]	19	90	19	90	1.000	0.566	1.766	7.63%
8	NTP 2018 (4)F [31]	18	90	19	90	0.947	0.531	1.690	7.37%
9	NTP 2018 (5)F [31]	18	90	19	90	0.947	0.531	1.690	7.37%
10	NTP 2018 (6)F [31]	19	90	19	90	1.000	0.566	1.766	7.63%
11	NTP 2018^ (1)F [32]	6	90	5	89	1.187	0.373	3.778	1.84%
12	NTP 2018^ (2)F [32]	8	90	5	89	1.582	0.534	4.686	2.09%
13	NTP 2018^ (3)F [32]	10	89	5	89	2.000	0.707	5.657	2.28%
14	NTP 2018^ (4)F [32]	2	89	5	89	0.400	0.079	2.030	0.94%
15	NTP 2018^ (5)F [32]	11	88	5	89	2.225	0.800	6.185	2.36%
16	NTP 2018^ (6)F [32]	8	90	5	89	1.582	0.534	4.686	2.09%
17	Smith 2007 (1) [83]	7	50	10	50	0.736	0.200	2.708	1.44%
18	Smith 2007 (2) [83]	6	49	10	50	0.700	0.286	1.711	3.12%
19	Smith 2007 (3) [83]	12	50	10	50	0.612	0.238	1.574	2.80%
20	Smith 2007 (4) [83]	5	50	8	50	1.200	0.566	2.544	4.42%
21	Smith 2007 (5) [83]	6	50	8	50	0.625	0.217	1.803	2.22%
22	Smith 2007 (6) [83]	7	50	8	50	0.750	0.277	2.030	2.52%
23	Toler 1997 [74]	4	187	5	172	0.875	0.339	2.256	2.78%
24	Tillmann 2007 (1) [84]	9	50	1	50	9.000	1.154	70.178	0.59%
25	Tillmann 2007 (2) [84]	4	50	1	50	4.000	0.451	35.488	0.52%
26	Tillmann 2007 (3) [84]	4	50	1	50	4.000	0.451	35.488	0.52%
27	Tillmann 2007 (4) [84]	5	50	5	50	1.000	0.304	3.289	1.76%
28	Tillmann 2007 (5) [84]	3	50	5	50	0.600	0.149	2.418	1.28%
29	Tillmann 2007 (6) [84]	5	50	5	50	1.000	0.304	3.289	1.76%
	Combined Effect Size					1.021	0.888	1.174	
	Heterogeneity	Tau = 0	$I^2 = 0$						
	Overall effect	z = 0.301	(p =0.763)						

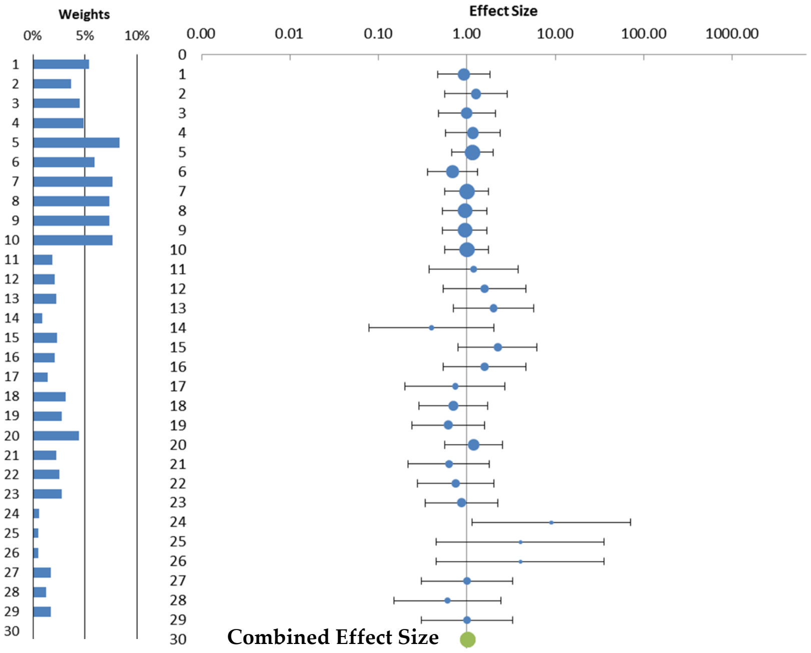

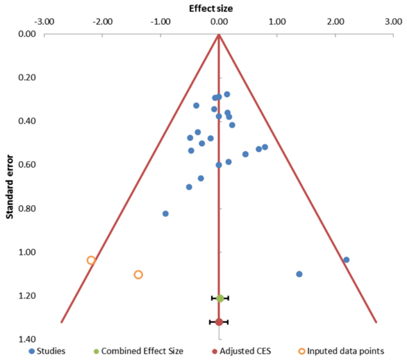

NTP 2018 [31] indicates NTP rat study, NTP 2018^ [32] indicates NTP mice study

Figure S2.30 Male Uro-Genital System Benign

		exposed		sham					
ID Study	study name	events	total number	events	total number	RR	lower limit	upper limit	weight %
1	Anderson 2004 (1) [75]	79	90	79	90	1.000	0.896	1.116	12.51%
2	Anderson 2004 (1) [75]	80	90	79	90	1.013	0.910	1.127	13.19%
3	Chou 1992 [63]	2	101	1	101	2.000	0.182	22.026	0.03%
4	La Regina 2003 (1) [76]	76	80	80	80	0.950	0.899	1.005	48.22%
5	La Regina 2003 (2) [76]	72	80	80	80	0.901	0.834	0.973	25.40%
6	NTP 2018 (1)M [31]	4	90	4	90	1.000	0.256	3.912	0.08%
7	NTP 2018 (2)M [31]	9	90	4	90	2.250	0.713	7.097	0.11%
8	NTP 2018 (3)M [31]	5	90	4	90	1.250	0.344	4.544	0.09%
9	NTP 2018 (4)M [31]	2	90	4	90	0.500	0.093	2.692	0.05%
10	NTP 2018 (5)M [31]	4	90	4	90	1.000	0.256	3.912	0.08%
11	NTP 2018 (6)M [31]	2	90	4	90	0.500	0.093	2.692	0.05%
12	NTP 2018^ (1)M [32]	2	90	3	90	0.667	0.113	3.942	0.05%
13	NTP 2018^ (2)M [32]	0	90	3	90	0.143	0.007	2.782	0.02%
14	NTP 2018^ (3)M [32]	0	90	3	90	0.143	0.007	2.782	0.02%
15	NTP 2018^ (4)M [32]	2	90	3	90	0.667	0.113	3.942	0.05%
16	NTP 2018^ (5)M [32]	1	91	3	90	0.330	0.034	3.158	0.03%
17	NTP 2018^ (6)M [32]	1	91	3	90	0.330	0.034	3.158	0.03%
	**Combined Effect Size**					**0.951**	**0.915**	**0.987**	
	**Heterogeneity**	**Tau = 0**	**$I^2 = 0$**						
	**Overall effect**	**z = -2.835**	**(p = 0.005)**						

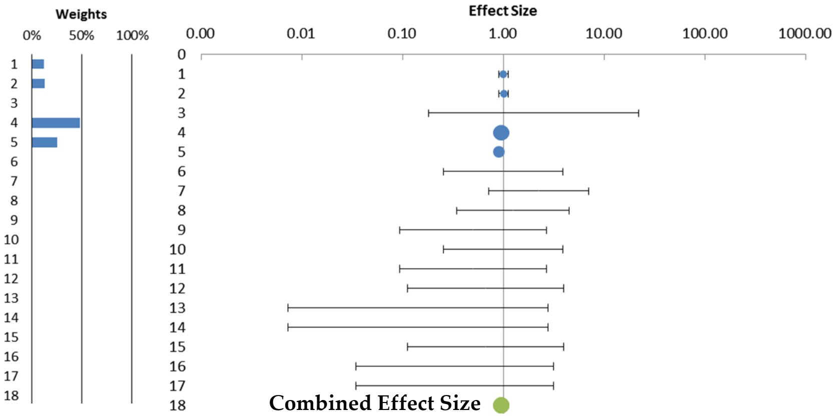

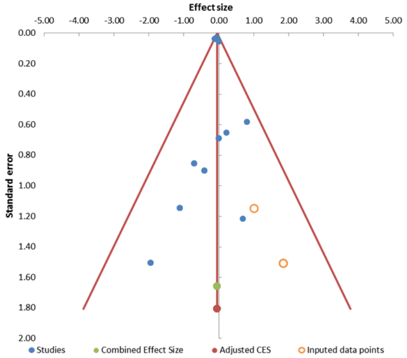

NTP 2018 [31] indicates NTP rat study, NTP 2018^ [32] indicates NTP mice study

Figure S2.31 Intestine Benign

ID Study	study name	exposed events	exposed total number	sham events	sham total number	RR	lower limit	upper limit	weight %
1	NTP 2018 (1) [31]	0	171	1	173	0.337	0.014	8.314	8.29%
2	NTP 2018 (2) [31]	0	174	1	173	0.331	0.013	8.171	8.29%
3	NTP 2018 (3) [31]	0	176	1	173	0.328	0.013	8.079	8.29%
4	NTP 2018 (4) [31]	0	173	1	173	0.333	0.014	8.218	8.29%
5	NTP 2018 (5) [31]	0	171	1	173	0.337	0.014	8.314	8.29%
6	NTP 2018 (6) [31]	0	164	1	173	0.352	0.014	8.668	8.30%
7	NTP 2018^ (1) [32]	1	171	0	172	3.017	0.122	74.398	8.29%
8	NTP 2018^ (2) [32]	2	174	0	172	4.943	0.236	103.309	9.22%
9	NTP 2018^ (3) [32]	0	170	0	172	1.012	0.020	51.410	5.52%
10	NTP 2018^ (4) [32]	1	174	0	172	2.966	0.120	73.120	8.29%
11	NTP 2018^ (5) [32]	2	166	0	172	5.180	0.248	108.266	9.22%
12	NTP 2018^ (6) [32]	3	171	0	172	7.041	0.363	136.715	9.68%
	Combined Effect Size					1.137	0.487	2.651	
	Heterogeneity	Tau = 0	$I^2 = 0$						
	Overall effect	z = 0.333	(p = 0.739)						

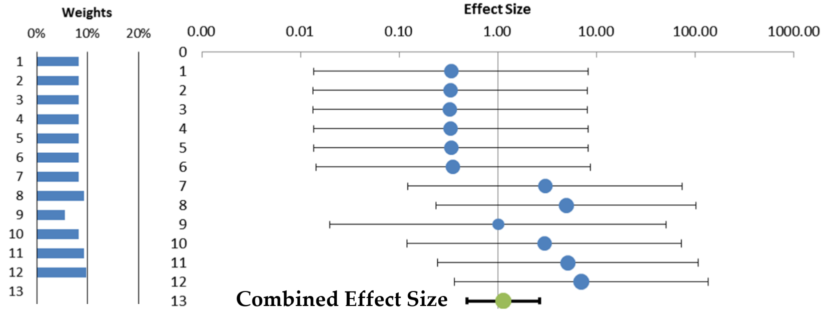

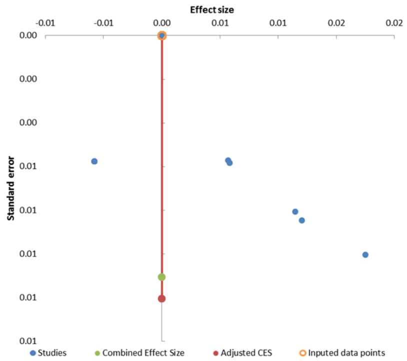

NTP 2018 [31] indicates NTP rat study, NTP 2018^ [32] indicates NTP mice study

Figure S2.32 Kidney Benign

		exposed		sham					
**ID Study**	**study name**	**events**	**total number**	**events**	**total number**	**RR**	**lower limit**	**upper limit**	**weight %**
1	Chou 1992 [63]	1	100	1	100	1.000	0.062	16.037	3.22%
2	NTP 2018 (1) [31]	4	180	6	180	0.667	0.191	2.332	15.71%
3	NTP 2018 (2) [31]	4	180	6	180	0.667	0.191	2.332	15.71%
4	NTP 2018 (3) [31]	3	179	6	180	0.503	0.127	1.989	13.04%
5	NTP 2018 (4) [31]	3	180	6	180	0.500	0.126	1.978	13.04%
6	NTP 2018 (5) [31]	3	180	6	180	0.500	0.126	1.978	13.04%
7	NTP 2018 (6) [31]	0	176	6	180	0.079	0.004	1.400	2.97%
8	NTP 2018^ (1) [32]	1	176	2	179	0.509	0.046	5.604	4.28%
9	NTP 2018^ (2) [32]	1	179	2	179	0.500	0.045	5.510	4.28%
10	NTP 2018^ (3) [32]	0	177	2	179	0.202	0.010	4.227	2.67%
11	NTP 2018^ (4) [32]	0	178	2	179	0.201	0.010	4.203	2.67%
12	NTP 2018^ (5) [32]	0	178	2	179	0.201	0.010	4.203	2.67%
13	NTP 2018^ (6) [32]	1	177	2	179	0.506	0.046	5.572	4.28%
14	Tillmann 2010 [73]	1	56	0	54	2.895	0.116	72.097	2.42%
	**Combined Effect Size**					**0.515**	**0.374**	**0.708**	
	**Heterogeneity**	**Tau = 0**	**$I^2 = 0$**						
	**Overall effect**	**z = -4.497**	**(p << 0.01)**						

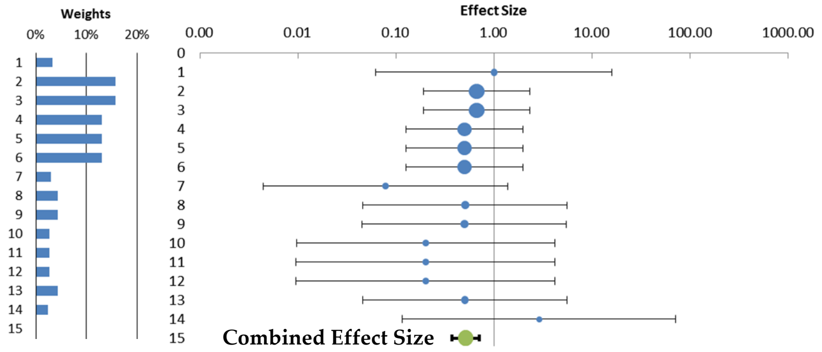

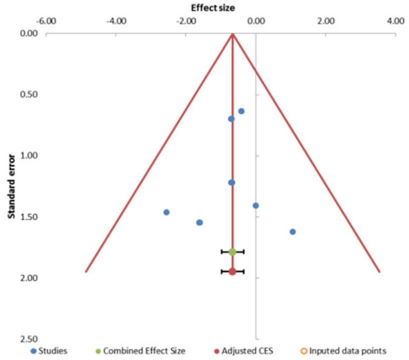

NTP 2018 [31] indicates NTP rat study, NTP 2018^ [32] indicates NTP mice study

Figure S2.33 Liver Benign

ID Study	study name	exposed events	exposed total number	sham events	sham total number	RR	lower limit	upper limit	weight %
1	Chou 1992 [63]	3	100	0	100	7.000	0.360	136.235	0.10%
2	Frei et al 1998 b [66]	12	100	13	100	0.923	0.441	1.932	1.52%
3	Frei et al 1998a [65]	10	99	9	93	1.044	0.442	2.468	1.13%
4	La Regina 2003 (1) [76]	0	160	1	160	0.333	0.014	8.221	0.08%
5	La Regina 2003 (2) [76]	3	160	1	160	3.000	0.313	28.782	0.17%
6	NTP 2018 (1) [31]	4	180	7	180	0.571	0.170	1.926	0.57%
7	NTP 2018 (2) [31]	1	180	7	180	0.143	0.018	1.157	0.20%
8	NTP 2018 (3) [31]	5	180	7	180	0.714	0.230	2.217	0.66%
9	NTP 2018 (4) [31]	4	180	7	180	0.571	0.170	1.926	0.57%
10	NTP 2018 (5) [31]	6	179	7	180	0.862	0.294	2.524	0.73%
11	NTP 2018 (6) [31]	1	178	7	180	0.144	0.018	1.170	0.20%
12	NTP 2018^ (1) [32]	79	179	71	179	1.113	0.871	1.422	9.91%
13	NTP 2018^ (2) [32]	80	180	71	179	1.121	0.878	1.431	9.97%
14	NTP 2018^ (3) [32]	65	179	71	179	0.915	0.702	1.194	8.84%
15	NTP 2018^ (4) [32]	90	177	71	179	1.282	1.016	1.617	10.67%
16	NTP 2018^ (5) [32]	77	180	71	179	1.078	0.841	1.382	9.76%
17	NTP 2018^ (6) [32]	82	180	71	179	1.149	0.902	1.463	10.11%
18	Oberto 2007 (1)	2	100	0	100	5.000	0.239	104.778	0.09%
19	Oberto 2007 (2)	0	100	0	100	1.000	0.020	51.124	0.06%
20	Oberto 2007 (3)	0	100	0	100	1.000	0.020	51.124	0.06%
21	Tillmann 2007 (1)	11	100	17	100	0.647	0.318	1.316	1.63%
22	Tillmann 2007 (2)	12	100	17	100	0.706	0.354	1.406	1.73%
23	Tillmann 2007 (3)	9	100	17	100	0.529	0.247	1.136	1.42%
24	Tillmann 2007 (4)	13	100	13	100	1.000	0.486	2.057	1.59%
25	Tillmann 2007 (5)	11	100	13	100	0.846	0.396	1.806	1.44%
26	Tillmann 2007 (6)	5	100	13	100	0.385	0.142	1.045	0.85%
27	Tillmann 2010	48	56	38	54	1.218	0.992	1.496	12.59%
28	Toler 1997	100	188	94	180	1.019	0.839	1.237	13.35%
	**Combined Effect Size**					**1.044**	**0.945**	**1.153**	
	**Heterogeneity**	Tau = 0.08	I^2 = 13.02%						
	**Overall effect**	z = 0.888	(p = 0.375)						

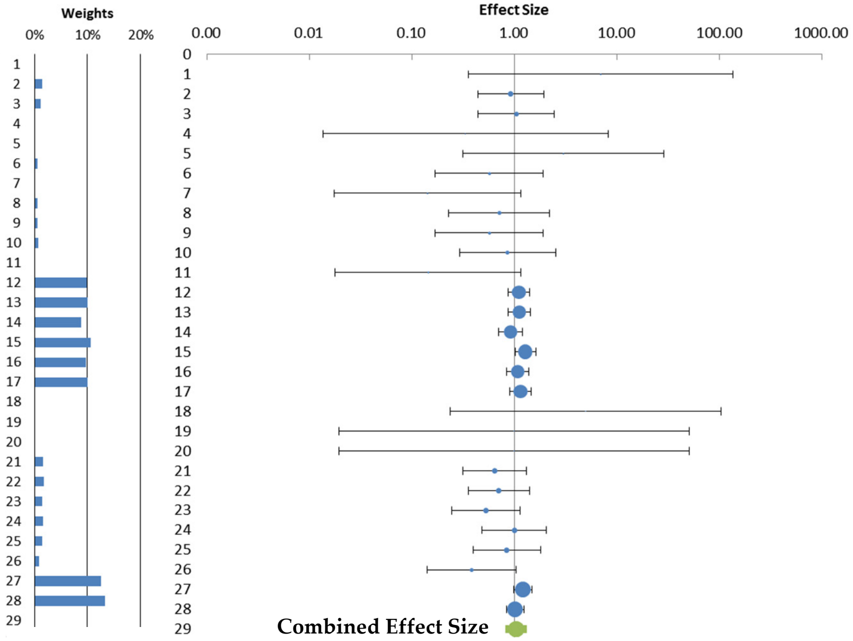

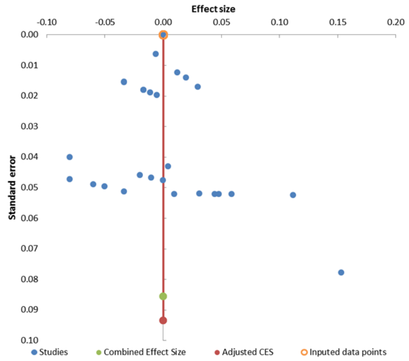

NTP 2018 [31] indicates NTP rat study, NTP 2018^ [32] indicates NTP mice study

Figure S2.34 Lung Benign

ID Study	study name	exposed events	exposed total number	sham events	sham total number	RR	lower limit	upper limit	weight %
1	Frei et al 1998 b [66]	3	100	4	100	0.750	0.171	3.295	1.65%
2	Frei et al 1998a [65]	0	99	4	97	0.109	0.006	2.032	0.42%
3	NTP 2018 (1) [31]	1	180	1	180	1.000	0.062	16.014	0.47%
4	NTP 2018 (2) [31]	0	180	1	180	0.333	0.014	8.217	0.35%
5	NTP 2018 (3) [31]	0	180	1	180	0.333	0.014	8.217	0.35%
6	NTP 2018 (4) [31]	2	180	1	180	2.000	0.181	22.039	0.62%
7	NTP 2018 (5) [31]	1	180	1	180	1.000	0.062	16.014	0.47%
8	NTP 2018 (6) [31]	0	180	1	180	0.333	0.014	8.217	0.35%
9	NTP 2018^ (1) [32]	19	180	16	180	1.188	0.630	2.239	8.92%
10	NTP 2018^ (2) [32]	25	180	16	180	1.563	0.862	2.831	10.15%
11	NTP 2018^ (3) [32]	18	180	16	180	1.125	0.591	2.140	8.67%
12	NTP 2018^ (4) [32]	14	179	16	180	0.880	0.442	1.753	7.56%
13	NTP 2018^ (5) [32]	20	180	16	180	1.250	0.668	2.338	9.15%
14	NTP 2018^ (6) [32]	14	180	16	180	0.875	0.439	1.743	7.55%
15	Oberto 2007 (1) [81]	2	100	3	100	0.667	0.113	3.947	1.14%
16	Oberto 2007 (2) [81]	1	100	3	100	0.333	0.035	3.194	0.71%
17	Oberto 2007 (3) [81]	4	100	3	100	1.333	0.303	5.858	1.65%
18	Tillmann 2007 (1) [84]	14	100	17	100	0.824	0.428	1.585	8.41%
19	Tillmann 2007 (2) [84]	15	100	17	100	0.882	0.465	1.674	8.79%
20	Tillmann 2007 (3) [84]	13	100	17	100	0.765	0.391	1.496	8.01%
21	Tillmann 2007 (4) [84]	9	100	9	100	1.000	0.412	2.427	4.59%
22	Tillmann 2007 (5) [84]	7	100	9	100	0.778	0.300	2.019	3.96%
23	Tillmann 2007 (6) [84]	11	100	9	100	1.222	0.527	2.835	5.10%
24	Tillmann 2010 [73]	2	56	2	54	0.964	0.138	6.749	0.96%
	**Combined Effect Size**					**0.994**	**0.870**	**1.135**	
	**Heterogeneity**	**Tau = 0**	**I^2= 0**						
	**Overall effect**	**z = -0.098**	**(p = 0.922)**						

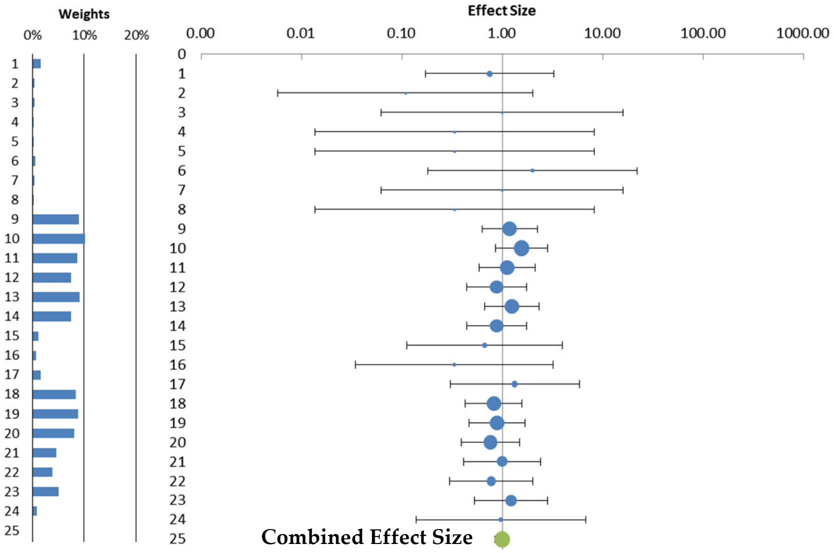

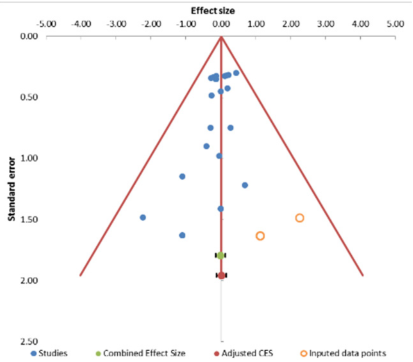

NTP 2018 [31] indicates NTP rat study, NTP 2018^ [32] indicates NTP mice study

Figure S2.35 Mammary Benign

ID Study	study name	exposed events	exposed total number	sham events	sham total number	RR	lower limit	upper limit	weight %
1	Anderson 2004 (1) [75]	40	180	26	180	1.538	0.981	2.413	4.16%
2	Anderson 2004 (2) [75]	29	180	26	180	1.115	0.684	1.819	3.52%
3	Frei et al 1998a [65]	2	96	1	89	1.854	0.168	20.417	0.15%
4	Jin 2010 [68]	2	41	1	41	2.000	0.182	21.988	0.15%
5	La Regina 2003 (1) [76]	9	160	8	160	1.125	0.444	2.852	0.97%
6	La Regina 2003 (2) [76]	12	160	8	160	1.500	0.628	3.583	1.11%
7	NTP 2018 (1) [31]	76	165	73	172	1.085	0.853	1.381	14.52%
8	NTP 2018 (2) [31]	66	171	73	172	0.909	0.703	1.177	12.66%
9	NTP 2018 (3) [31]	67	172	73	172	0.918	0.710	1.186	12.80%
10	NTP 2018 (4) [31]	70	167	73	172	0.988	0.769	1.268	13.45%
11	NTP 2018 (5) [31]	66	170	73	172	0.915	0.707	1.184	12.68%
12	NTP 2018 (6) [31]	65	170	73	172	0.901	0.695	1.168	12.51%
13	NTP 2018^ (1) [32]	0	93	0	87	0.936	0.018	47.939	0.05%
14	NTP 2018^ (2) [32]	0	90	0	87	0.967	0.019	49.534	0.05%
15	NTP 2018^ (3) [32]	0	92	0	87	0.946	0.018	48.459	0.05%
16	NTP 2018^ (4) [32]	1	88	0	87	2.966	0.120	73.463	0.08%
17	NTP 2018^ (5) [32]	2	90	0	87	4.835	0.231	101.408	0.09%
18	NTP 2018^ (6) [32]	0	91	0	87	0.957	0.019	48.991	0.05%
19	Smith 2007 (1) [83]	16	100	11	100	1.455	0.708	2.989	1.63%
20	Smith 2007 (2) [83]	12	100	11	100	1.091	0.503	2.367	1.41%
21	Smith 2007 (3) [83]	12	100	11	100	1.091	0.503	2.367	1.41%
22	Smith 2007 (4) [83]	14	100	18	100	0.778	0.408	1.482	2.03%
23	Smith 2007 (5) [83]	15	100	18	100	0.833	0.444	1.565	2.13%
24	Smith 2007 (6) [83]	17	99	18	100	0.954	0.521	1.748	2.31%
	**Combined Effect Size**					**0.993**	**0.928**	**1.062**	
	**Heterogeneity**	**Tau = 0**	**I^2= 0**						
	**Overall effect**	**z = -0.217**	**(p = 0.828)**						

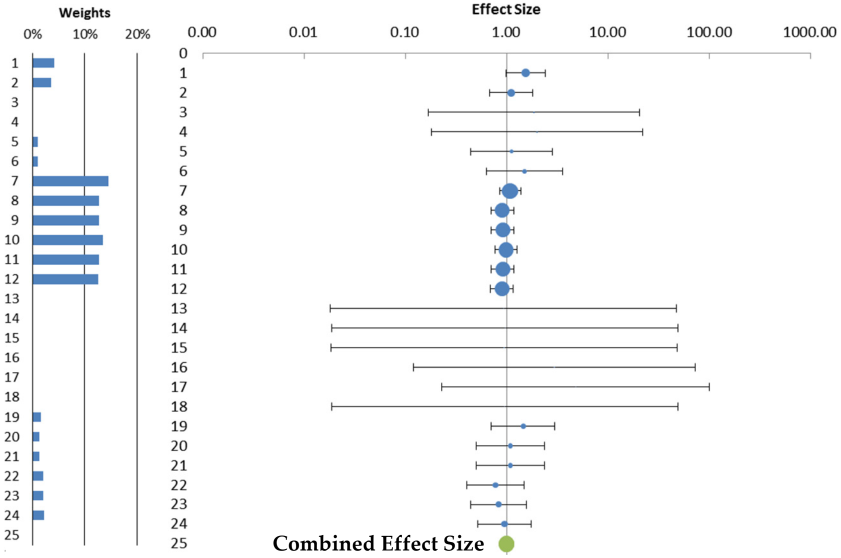

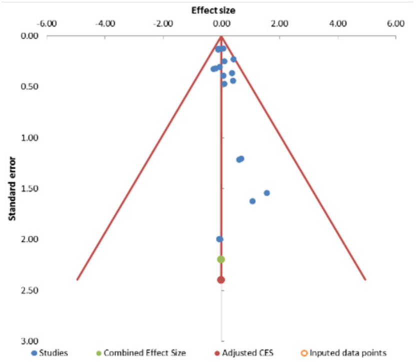

NTP 2018 [31] indicates NTP rat study, NTP 2018^ [32] indicates NTP mice study

Figure S2.36 Pancreas Benign

ID Study	study name	exposed events	exposed total number	sham events	sham total number	RR	lower limit	upper limit	weight %
1	Chou 1992 [63]	1	100	2	100	0.500	0.045	5.506	0.56%
2	Frei et al 1998 b [66]	2	100	1	99	1.980	0.180	21.805	0.56%
3	Frei et al 1998a [65]	1	95	0	93	2.938	0.119	72.703	0.31%
4	La Regina 2003 (1) [76]	4	160	7	160	0.571	0.170	1.923	2.18%
5	La Regina 2003 (2) [76]	2	160	7	160	0.286	0.060	1.362	1.31%
6	NTP 2018 (1) [31]	39	178	28	180	1.409	0.907	2.188	16.50%
7	NTP 2018 (2) [31]	31	178	28	180	1.120	0.701	1.789	14.58%
8	NTP 2018 (3) [31]	21	173	28	180	0.780	0.460	1.323	11.50%
9	NTP 2018 (4) [31]	37	178	28	180	1.336	0.855	2.089	16.05%
10	NTP 2018 (5) [31]	39	177	28	180	1.416	0.912	2.200	16.51%
11	NTP 2018 (6) [31]	18	167	28	180	0.693	0.398	1.207	10.38%
12	NTP 2018^ (1) [32]	0	178	0	175	0.983	0.019	49.951	0.21%
13	NTP 2018^ (2) [32]	1	180	0	175	2.917	0.118	71.913	0.31%
14	NTP 2018^ (3) [32]	3	177	0	175	6.921	0.356	134.387	0.36%
15	NTP 2018^ (4) [32]	1	178	0	175	2.950	0.120	72.719	0.31%
16	NTP 2018^ (5) [32]	1	178	0	175	2.950	0.120	72.719	0.31%
17	NTP 2018^ (6) [32]	1	176	0	175	2.983	0.121	73.542	0.31%
18	Smith 2007 (1) [83]	4	100	5	100	0.800	0.220	2.915	1.93%
19	Smith 2007 (2) [83]	4	100	5	100	0.800	0.220	2.915	1.93%
20	Smith 2007 (3) [83]	1	100	5	100	0.200	0.023	1.704	0.70%
21	Smith 2007 (4) [83]	3	100	3	100	1.000	0.205	4.883	1.28%
22	Smith 2007 (5) [83]	1	100	3	100	0.333	0.035	3.194	0.63%
23	Smith 2007 (6) [83]	3	100	3	100	1.000	0.205	4.883	1.28%
	Combined Effect Size					1.082	0.906	1.293	

Heterogeneity Tau = 0 $I^2 = 0$

Overall effect z = 0.918 (p = 0.359)

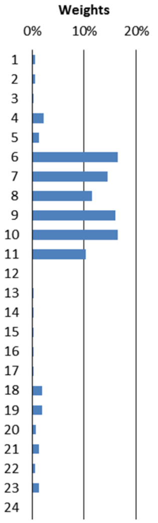

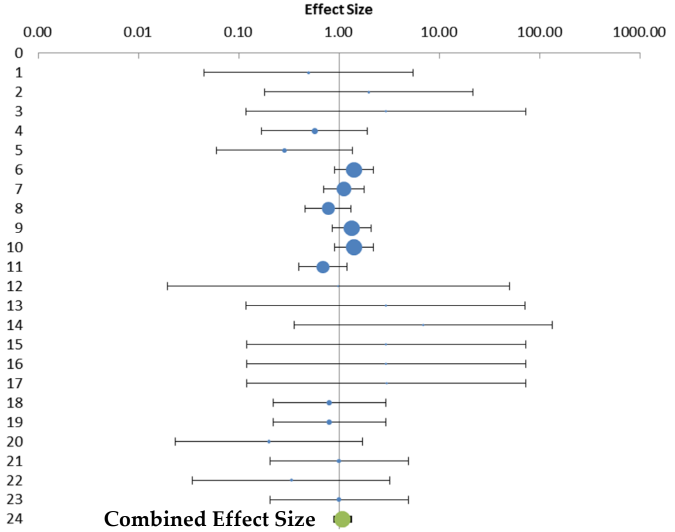

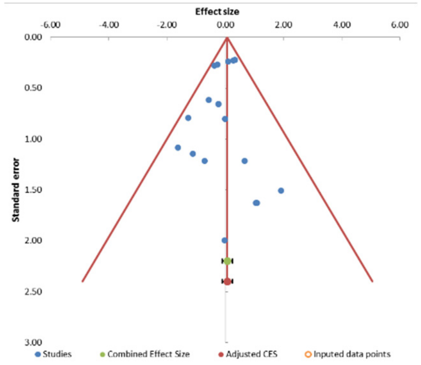

NTP 2018 [31] indicates NTP rat study, NTP 2018^ [32] indicates NTP mice study

Figure S2.37 Pituitary Benign

ID Study	study name	exposed events	exposed total number	sham events	sham total number	RR	lower limit	upper limit	weight %
1	Chou 1992 [63]	17	100	21	100	0.810	0.453	1.446	1.88%
2	Frei et al 1998a [65]	1	95	0	90	2.844	0.115	70.392	0.06%
3	La Regina 2003 (1) [76]	21	160	26	160	0.808	0.474	1.377	2.21%
4	La Regina 2003 (2) [76]	25	160	26	160	0.962	0.580	1.594	2.47%
5	NTP 2018 (1) [31]	61	180	60	179	1.011	0.756	1.353	7.43%
6	NTP 2018 (2) [31]	64	180	60	179	1.061	0.797	1.412	7.70%
7	NTP 2018 (3) [31]	58	180	60	179	0.961	0.714	1.293	7.15%
8	NTP 2018 (4) [31]	66	179	60	179	1.100	0.829	1.459	7.89%
9	NTP 2018 (5) [31]	64	179	60	179	1.067	0.802	1.420	7.71%
10	NTP 2018 (6) [31]	53	180	60	179	0.878	0.646	1.194	6.68%
11	NTP 2018^ (1) [32]	5	165	6	166	0.838	0.260	2.705	0.46%
12	NTP 2018^ (2) [32]	7	171	6	166	1.133	0.387	3.312	0.55%
13	NTP 2018^ (3) [32]	5	169	6	166	0.819	0.254	2.642	0.46%
14	NTP 2018^ (4) [32]	8	163	6	166	1.358	0.480	3.842	0.58%
15	NTP 2018^ (5) [32]	10	177	6	166	1.563	0.579	4.220	0.64%
16	NTP 2018^ (6) [32]	1	169	6	166	0.164	0.020	1.355	0.14%
17	Oberto 2007 (1) [81]	11	100	7	100	1.571	0.631	3.911	0.76%
18	Oberto 2007 (2) [81]	12	100	7	100	1.714	0.700	4.197	0.79%
19	Oberto 2007 (3) [81]	11	100	7	100	1.571	0.631	3.911	0.76%
20	Smith 2007 (1) [83]	37	100	44	100	0.841	0.598	1.182	5.47%
21	Smith 2007 (2) [83]	49	100	44	100	1.114	0.825	1.503	7.04%
22	Smith 2007 (3) [83]	47	100	44	100	1.068	0.787	1.450	6.78%
23	Smith 2007 (4) [83]	50	100	44	100	1.136	0.844	1.530	7.16%
24	Smith 2007 (5) [83]	44	100	44	100	1.000	0.730	1.370	6.40%
25	Smith 2007 (6) [83]	44	100	44	100	1.000	0.730	1.370	6.40%
26	Tillmann 2007 (1) [84]	10	100	9	100	1.111	0.469	2.631	0.85%
27	Tillmann 2007 (2) [84]	9	99	9	100	1.010	0.416	2.451	0.81%
28	Tillmann 2007 (3) [84]	10	100	9	100	1.111	0.469	2.631	0.85%
29	Tillmann 2007 (4) [84]	11	100	6	100	1.833	0.701	4.794	0.69%
30	Tillmann 2007 (5) [84]	12	99	6	99	2.000	0.777	5.148	0.71%
31	Tillmann 2007 (6) [84]	6	100	6	100	1.000	0.332	3.016	0.52%
	**Combined Effect Size**					**1.033**	**0.972**	**1.097**	
	**Heterogeneity**	Tau = 0	$I^2 = 0$						
	**Overall effect**	z = 1.086	(p = 0.278)						

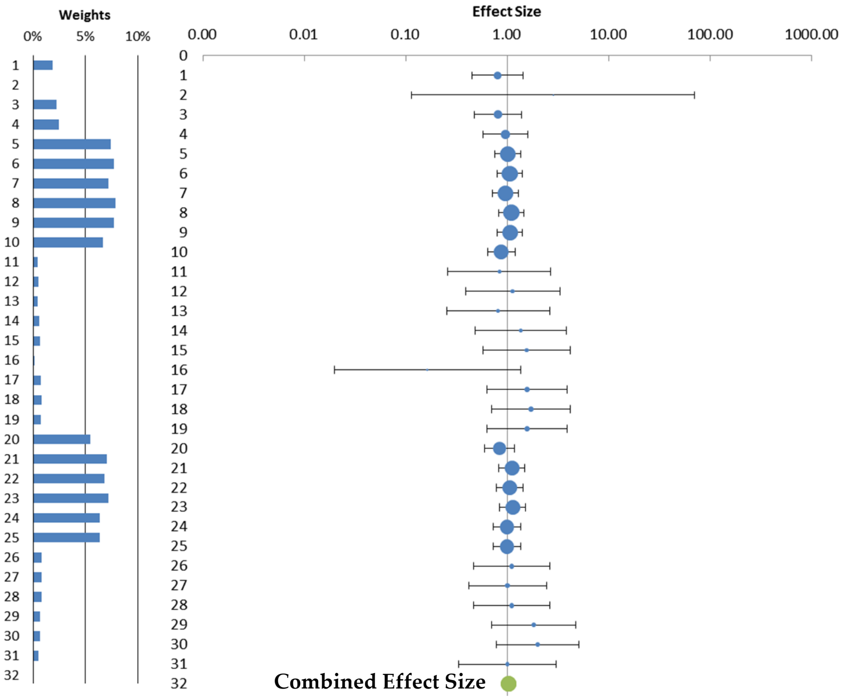

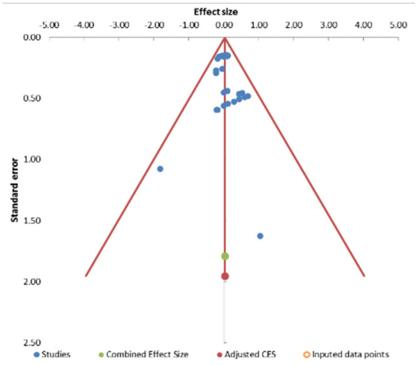

NTP 2018 [31] indicates NTP rat study, NTP 2018^ [32] indicates NTP mice study

Figure S2.38 Skin Benign

ID Study	study name	exposed events	exposed total number	sham events	sham total number	RR	lower limit	upper limit	weight %
1	Chou 1992 [63]	7	100	2	100	3.500	0.738	16.595	3.47%
2	NTP 2018 (1) [31]	15	180	15	180	1.000	0.503	1.989	13.01%
3	NTP 2018 (2) [31]	22	180	15	180	1.467	0.785	2.740	14.72%
4	NTP 2018 (3) [31]	16	180	15	180	1.067	0.543	2.096	13.31%
5	NTP 2018 (4) [31]	23	180	15	180	1.533	0.826	2.847	14.90%
6	NTP 2018 (5) [31]	24	180	15	180	1.600	0.867	2.954	15.08%
7	NTP 2018 (6) [31]	18	180	15	180	1.200	0.623	2.311	13.85%
8	NTP 2018^ (1) [32]	1	180	4	180	0.250	0.028	2.231	1.82%
9	NTP 2018^ (2) [32]	2	180	4	180	0.500	0.092	2.711	2.97%
10	NTP 2018^ (3) [32]	0	180	4	180	0.111	0.006	2.069	1.04%
11	NTP 2018^ (4) [32]	0	180	4	180	0.111	0.006	2.069	1.04%
12	NTP 2018^ (5) [32]	1	180	4	180	0.250	0.028	2.231	1.82%
13	NTP 2018^ (6) [32]	2	180	4	180	0.500	0.092	2.711	2.97%
	**Combined Effect Size**					**1.140**	**0.797**	**1.630**	

Heterogeneity **Tau = 0.245** **I^2= 21.32%**
Overall effect **z = 0.796** **(p = 0.426)**

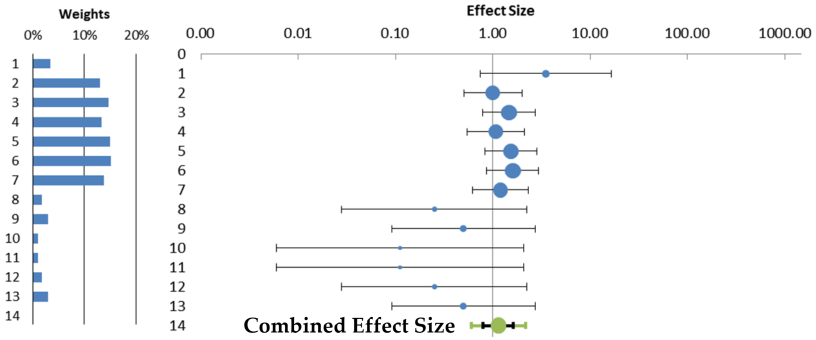

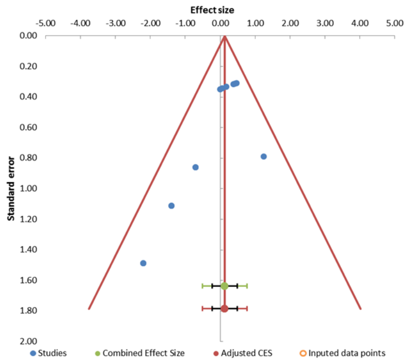

NTP 2018 [31] indicates NTP rat study, NTP 2018^ [32] indicates NTP mice study

Figure S2.39 Stomach Benign

ID Study	study name	exposed events	exposed total number	sham events	sham total number	RR	lower limit	upper limit	weight %
1	Chou 1992 [63]	3	100	4	100	0.750	0.171	3.295	22.13%
2	Frei et al 1998a [65]	1	100	2	94	0.470	0.043	5.176	8.43%
3	NTP 2018 (1) [31]	0	180	1	180	0.333	0.014	8.217	4.69%
4	NTP 2018 (2) [31]	1	180	1	180	1.000	0.062	16.014	6.27%
5	NTP 2018 (3) [31]	0	180	1	180	0.333	0.014	8.217	4.69%
6	NTP 2018 (4) [31]	0	180	1	180	0.333	0.014	8.217	4.69%
7	NTP 2018 (5) [31]	0	179	1	180	0.335	0.014	8.263	4.69%
8	NTP 2018 (6) [31]	1	180	1	180	1.000	0.062	16.014	6.27%
9	NTP 2018^ (1) [32]	1	176	1	174	0.989	0.062	15.833	6.27%
10	NTP 2018^ (2) [32]	2	179	1	174	1.944	0.176	21.423	8.37%
11	NTP 2018^ (3) [32]	0	172	1	174	0.337	0.014	8.313	4.69%
12	NTP 2018^ (4) [32]	1	177	1	174	0.983	0.061	15.743	6.27%
13	NTP 2018^ (5) [32]	1	173	1	174	1.006	0.063	16.108	6.27%
14	NTP 2018^ (6) [32]	1	174	1	174	1.000	0.062	16.015	6.27%
	**Combined Effect Size**					**0.719**	**0.525**	**0.985**	
	**Heterogeneity**	**Tau = 0**	**I^2= 0**						
	**Overall effect**	**z = -2.265**	**(p = 0.023)**						

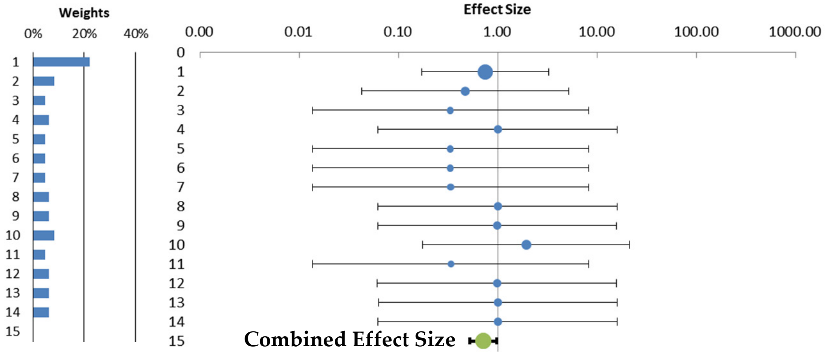

NTP 2018 [31] indicates NTP rat study, NTP 2018^ [32] indicates NTP mice study

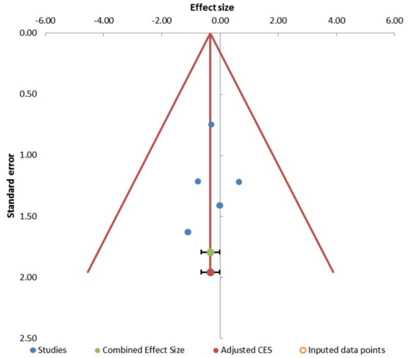

Figure S2.40 Thymus Benign

ID Study	study name	exposed events	exposed total number	sham events	sham total number	RR	lower limit	upper limit	weight %
1	NTP 2018 (1) [31]	3	172	1	175	3.052	0.318	29.289	3.23%
2	NTP 2018 (2) [31]	1	176	1	175	0.994	0.062	15.924	2.15%
3	NTP 2018 (3) [31]	0	172	1	175	0.339	0.014	8.361	1.61%
4	NTP 2018 (4) [31]	0	168	1	175	0.347	0.014	8.559	1.61%
5	NTP 2018 (5) [31]	1	174	1	175	1.006	0.063	16.107	2.15%
6	NTP 2018 (6) [31]	0	169	1	175	0.345	0.014	8.509	1.61%
7	NTP 2018^ (1) [32]	0	163	0	160	0.982	0.019	49.911	1.07%
8	NTP 2018^ (2) [32]	1	165	0	160	2.910	0.118	71.758	1.61%
9	NTP 2018^ (3) [32]	0	158	0	160	1.013	0.020	51.488	1.07%
10	Smith 2007 (1) [83]	6	97	8	98	0.758	0.271	2.116	15.76%
11	Smith 2007 (2) [83]	6	99	8	98	0.742	0.266	2.074	15.74%
12	Smith 2007 (3) [83]	5	99	8	98	0.619	0.208	1.838	14.02%
13	Smith 2007 (4) [83]	8	100	6	94	1.253	0.449	3.500	15.76%
14	Smith 2007 (5) [83]	3	97	6	94	0.485	0.124	1.898	8.92%
15	Smith 2007 (6) [83]	6	95	6	94	0.989	0.329	2.979	13.69%
	**Combined Effect Size**					**0.830**	**0.645**	**1.067**	
	**Heterogeneity**	**Tau = 0**	**I^2 = 0**						
	**Overall effect**	**z = -1.594**	**(p = 0.111)**						

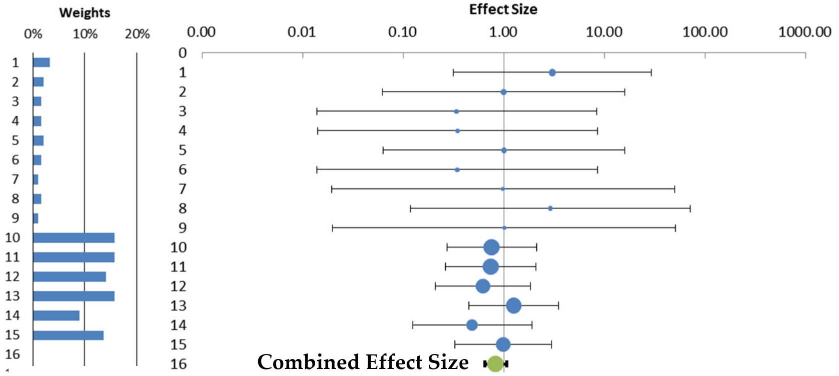

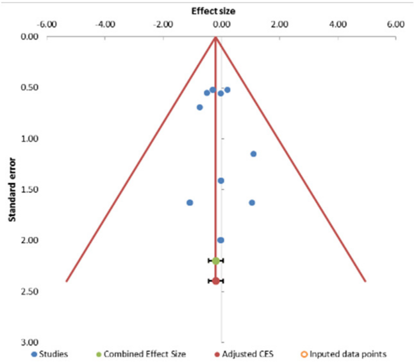

NTP 2018 [31] indicates NTP rat study, NTP 2018^ [32] indicates NTP mice study

Figure S2.41 Thyroid Benign

		exposed		sham					
ID Study	study name	events	total number	events	total number	RR	lower limit	upper limit	weight %
1	Anderson 2004 (1) [75]	17	180	25	180	0.680	0.380	1.217	8.09%
2	Anderson 2004 (2) [75]	17	180	25	180	0.680	0.380	1.217	8.09%
3	Chou 1992 [63]	10	100	9	100	1.111	0.469	2.631	3.71%
4	Frei et al 1998a [65]	1	84	0	86	3.071	0.124	76.067	0.27%
5	La Regina 2003 (1) [76]	10	160	8	160	1.250	0.505	3.096	3.34%
6	La Regina 2003 (2) [76]	13	160	8	160	1.625	0.690	3.826	3.75%
7	NTP 2018 (1) [31]	22	177	14	179	1.589	0.839	3.012	6.72%
8	NTP 2018 (2) [31]	17	179	14	179	1.214	0.616	2.394	5.96%
9	NTP 2018 (3) [31]	21	175	14	179	1.534	0.805	2.926	6.59%
10	NTP 2018 (4) [31]	23	177	14	179	1.661	0.882	3.130	6.85%
11	NTP 2018 (5) [31]	18	176	14	179	1.308	0.670	2.553	6.13%
12	NTP 2018 (6) [31]	20	174	14	179	1.470	0.765	2.822	6.45%
13	NTP 2018^ (1) [32]	0	177	0	175	0.989	0.019	50.233	0.18%
14	NTP 2018^ (2) [32]	0	174	0	175	1.006	0.020	51.098	0.18%
15	NTP 2018^ (3) [32]	0	174	0	175	1.006	0.020	51.098	0.18%
16	NTP 2018^ (4) [32]	3	176	0	175	6.960	0.358	135.147	0.31%
17	NTP 2018^ (5) [32]	2	176	0	175	4.972	0.238	103.907	0.30%
18	NTP 2018^ (6) [32]	2	175	0	175	5.000	0.239	104.499	0.30%
19	Smith 2007 (1) [83]	14	100	14	100	1.000	0.501	1.996	5.78%
20	Smith 2007 (2) [83]	12	100	14	100	0.857	0.416	1.768	5.27%
21	Smith 2007 (3) [83]	18	100	14	100	1.286	0.675	2.451	6.63%
22	Smith 2007 (4) [83]	8	100	13	100	0.615	0.265	1.427	3.90%
23	Smith 2007 (5) [83]	13	100	13	100	1.000	0.486	2.057	5.30%
24	Smith 2007 (6) [83]	15	100	13	100	1.154	0.577	2.308	5.74%
	Combined Effect Size					1.139	0.978	1.327	
	Heterogeneity	Tau = 0	$I^2 = 0$						
	Overall effect	z = 1.773	(p = 0.076)						

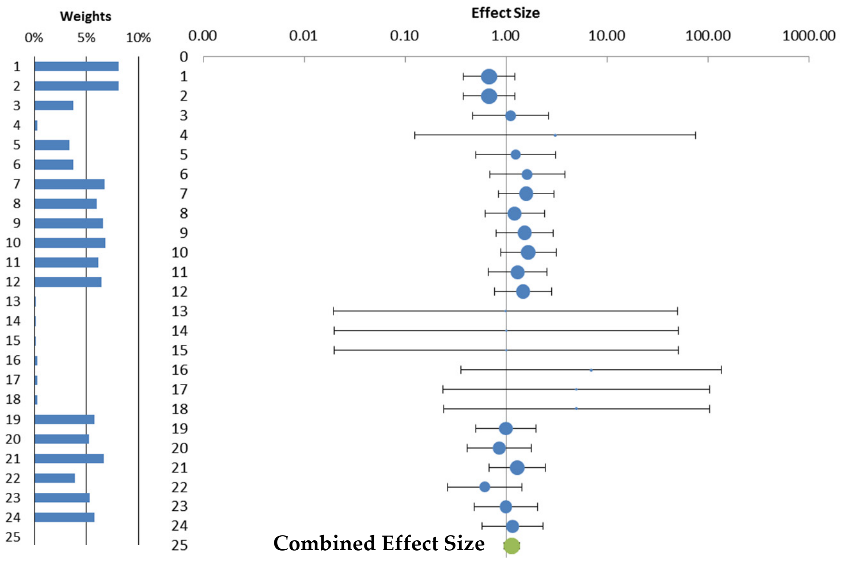

NTP 2018 [31] indicates NTP rat study, NTP 2018^ [32] indicates NTP mice study
